# Mental Health, Burnout, and Turnover Intention Among Nurses in Conflict-Affected Yemen: A Multicenter Study Across Eight Accessible Governorates

**DOI:** 10.3390/nursrep16100353

**Published:** 2026-10-07

**Authors:** Khalil A. Saleh, Naif Taleb Ali, Abdulhafith Alharbi

**Affiliations:** 1 Department of Medical Surgery, College of Nursing, University of Hail, Hail 81422, Saudi Arabia; 2 Department of Health Sciences, Faculty of Medicine and Health Sciences, University of Science and Technology, Aden, Yemen; 3 Department of Laboratory Sciences, Radfan College University, University of Lahej, Al-Houta, Yemen; 4 Department of Psychiatric and Mental Health Nursing, College of Nursing, University of Hail, Hail 81422, Saudi Arabia

**Keywords:** armed conflict, burnout, depression, anxiety, nurses, mental health, nursing leadership, turnover intention, workplace violence, Yemen

## Abstract

**Background**: Yemen’s long-standing armed conflict has decimated the health system and subjected nurses to unprecedented levels of occupational and conflict-related stress. The role of nurses is pivotal, yet little is known about their mental health in Yemen, limiting the evidence available to inform nursing leadership and workforce protection strategies. **Methods**: A multicenter cross-sectional study was carried out between January and December 2025, involving 800 registered nurses from 24 health facilities across eight conflict-affected Yemeni governorates using a multistage sampling strategy with probability-based selection of facilities and nurses within accessible governorates. Validated Arabic PHQ-9 and GAD-7 questionnaires (cut-off ≥ 10) were used to assess depression and anxiety. Burnout was assessed using a single-item self-rated measure with pilot validation against the Maslach Burnout Inventory emotional exhaustion subscale (r = 0.82), selected for pragmatic feasibility in a highly stressed population. Mixed-effects logistic regression was used to account for clustering at the facility level. Exploratory supplementary analyses examined associations involving self-rated burnout and reported availability of psychological support. **Results**: 52.0% (95% CI: 48.5–55.5) of nurses experienced moderate-to-severe depressive symptoms, while 47.0% (95% CI: 43.5–50.5) experienced moderate-to-severe anxiety symptoms. High self-rated burnout was prevalent in 34.0% (95% CI: 30.7–37.4), and 54.8% (95% CI: 51.2–58.3) reported their intention to leave within 12 months. Psychological distress was independently associated with displacement (AOR = 2.31, 95% CI: 1.68–3.17), workplace violence (AOR = 2.84, 95% CI: 2.03–3.97), inadequate income (AOR = 2.53, 95% CI: 1.80–3.56), nurse-to-patient ratios ≥ 1:20 (AOR = 1.68, 95% CI: 1.07–2.63), and frequent night shifts (AOR = 1.26, 95% CI: 1.17–1.36 per additional shift). Not all expected associations were significant; nurse-to-patient ratios of 1:15, facility type, and type of employment showed no consistent associations. Sampling-weighted prevalence estimates were very similar to the unweighted estimates (absolute differences < 1 percentage point), supporting the robustness of the findings against differential selection probabilities across governorates. **Conclusions**: Armed conflict and structural workforce stressors were associated with a substantial mental health burden among nurses in southern, central, and eastern governorates in Yemen. Because the most heavily conflict-affected northern governorates were inaccessible for security reasons, the findings are generalizable only to accessible areas, and the direction of difference in burden in inaccessible areas cannot be determined from these data. While the cross-sectional design precludes causal inference, the findings identify potential targets for intervention. Mental health support, workplace violence prevention, and safe staffing in nursing leadership and humanitarian workforce strategies may be relevant to workforce resilience strategies in fragile health systems, but these are implications consistent with observed associations rather than evidence of intervention effectiveness. Findings should be interpreted given the cross-sectional design, limited geographical access, and the use of a single-item self-rated burnout measure that does not capture the multidimensional nature of burnout. Future longitudinal studies should include multidimensional burnout instruments and trauma-related assessment.

## 1. Introduction

Nurses are the backbone of health systems, particularly in fragile and conflict-affected settings where human resources have become the main driver of service continuity. Emerging evidence indicates that psychological distress in nurses—depression, anxiety, and burnout—is consistently associated with poorer quality of care, more errors, and attrition of the workforce; therefore, it poses a major threat to health system performance [1,2]. Thus, investment in the health workforce, particularly nurses, is critical for strengthening health systems and resilience [3].

Globally, clinically significant depressive symptoms affect 22–30% of nurses, and up to 40% report moderate to severe anxiety [4,5]. Burnout is an occupational phenomenon [6] that is now alarmingly high and manifests as emotional exhaustion, depersonalization, and reduced sense of accomplishment. Psychological distress is linked to increased turnover intention, reduced retention, and disrupted continuity of care [7].

These vulnerabilities are compounded in conflict-affected settings where nurses are exposed to chronic insecurity, displacement, salary disruption, excessive workloads, and violence, often with little support. Evidence from Syria, Iraq, Palestine, and Afghanistan demonstrates much higher levels of distress among health workers, resulting in a “dual burden” of occupational stress compounded with trauma exposure [8]. The shifting therapeutic geographies of conflict, as evidenced in Iraq and Syria, further illustrate how war disrupts healthcare delivery and workforce well-being [8].

Yemen is one of the world’s worst humanitarian crises, characterized by protracted armed conflict, shattered health systems, and widespread displacement. Over half of the health facilities are partially or totally closed [9]. Despite the indispensable role of nurses, their mental health is profoundly under-researched [10].

The present study is conceptually based on the Job Demands–Resources (JD-R) model that argues that high levels of job demand in combination with low levels of resources are associated with burnout and depressive symptomatology [11]. In conflict settings, the ‘demands’ side is magnified by exogenous stressors (violence, displacement), while the ‘resources’ side is often ravaged by institutional collapse and disrupted salaries. These pathways are intensified by exposure to trauma and ongoing insecurity, which independently predict anxiety and depression through activation of stress-response systems [12]. Importantly, the JD-R framework is used here as an interpretive lens to guide variable selection and contextualize findings, rather than as a formal model to be statistically tested.

From this framework, we hypothesized that conflict-related job demands and poor resources would be related to psychological distress. Within this framework, burnout was examined as an indicator of occupational strain. Hence, this study aimed to (1) estimate the prevalence of depression, anxiety, burnout, and turnover intention; (2) identify occupational and conflict-related factors associated with these outcomes; and (3) explore associations involving burnout and psychological support within the JD-R framework. These analyses are exploratory, and the cross-sectional design does not permit causal inference.

From a health systems perspective, workforce erosion through psychological attrition is not merely a human resource issue but rather a fundamental threat to service continuity. Hence, knowing the determinants of distress among nurses is crucial for humanitarian actors and policymakers who want to preserve functional health systems in active conflict zones. Despite critical insights from other conflict zones, the mental health of Yemeni nurses is a profound evidence gap. To our knowledge, this is the first multicenter probability-based study examining depression, anxiety, burnout, and turnover intention among nurses across multiple conflict-affected governorates in Yemen.

Primary and Exploratory Analyses. The primary objectives of this study were twofold: (1) to estimate the prevalence of depressive symptoms, anxiety symptoms, burnout, and turnover intention and (2) to identify occupational and conflict-related factors associated with these outcomes. These primary analyses were specified as a priori in the study protocol (Supplementary File S1). Moreover, we performed exploratory analyses to study associations between burnout and psychological support in the JD-R framework. The exploratory analyses included structural equation modelling (SEM), mediation, moderation, and interaction analyses. These exploratory analyses were defined as exploratory in the protocol but went beyond the three primary aims, including additional hypotheses. They should be considered hypothesis-generating, and the cross-sectional design prevents causal inference. The distinction between primary and exploratory analyses is maintained across the manuscript.

## 2. Materials and Methods

### 2.1. Design, Setting, and Participants

A multicenter cross-sectional study was conducted between January and December 2025 among registered nurses working in active health facilities in eight governorates in conflict-affected Yemen. Ethical approval was obtained from the Institutional Review Board (IRB) of Radfan College University, Lahej, Yemen (IRB Reference Number: YEM-IRB-2025-094, Date of Approval: 15 January 2025; Expiry Date: 14 January 2026). Pilot testing (*n* = 40) was conducted from 16–19 January 2025, after IRB approval and prior to the main data collection. Main participant recruitment and data collection began on 20 January 2025, after IRB approval. No participant was enrolled in either the pilot or the main study prior to 15 January 2025, and no data were collected before ethical approval was granted. The study was conducted in accordance with the Declaration of Helsinki and ethical guidance for research in conflict-affected settings. All participants provided written informed consent (consent forms in Arabic and English are included in Supplementary File S2).

### 2.2. Participants and Inclusion Criteria

The inclusion criteria were (1) being a licensed registered nurse and currently working in Yemen; (2) having worked in a clinical setting for at least three consecutive months prior to data collection; and (3) providing informed consent (the consent forms are included in Arabic and English in Supplementary File S2). The exclusion criteria were (1) individuals in administrative roles only and without patient contact, (2) nurses on extended leave, and (3) refusal to participate.

### 2.3. Sample Size and Sampling Method

#### 2.3.1. Sample Size Calculation

Sample size was calculated using Cochran’s formula for prevalence estimation [13], based on the following parameters: expected prevalence = 50% (conservative estimate from regional studies), margin of error = 5%, confidence level = 95%, and a design effect of 1.5 for cluster sampling (full calculations in Supplementary File S1). The design effect of 1.5 was selected based on an assumed intra-cluster correlation coefficient (ICC) of 0.05 and an average cluster size of 11 nurses per facility, yielding a design effect of 1 + (11 − 1) × 0.05 = 1.5. This assumption was conservative based on previous studies in similar settings. The initial sample size estimate was 576 nurses. This was increased to 800 participants to compensate for anticipated non-response (approximately 10%) and to permit subgroup analyses (e.g., by governorate and facility type). The final sample of 800 provided 80% power to detect odds ratios of ≥1.5 for binary outcomes and effect sizes of ≥0.2 SD for continuous outcomes.

#### 2.3.2. Sampling Strategy

A multistage sampling strategy was used. Because security and accessibility constraints prevented the inclusion of all conflict-affected governorates, probability sampling procedures were applied within a predefined accessible sampling frame as follows:

Stage 1: Selection of Eligible Governorates. Due to security constraints, only accessible conflict-affected governorates were eligible for inclusion. Yemen has 22 governorates, of which 15 were classified as conflict-affected. Eight accessible governorates (Aden, Lahj, Abyan, Shabwah, Hadramawt, Al-Mahra, Socotra, and Taiz) were included in the sampling frame. For safety reasons, seven governorates under active siege or with total health facility closure (Sa’ada, Hajjah, Amran, Sana’a, Ibb, Dhamar, and Al-Bayda) were excluded. This classification as “conflict-affected” is consistent with the WHO definition of disruption to health systems caused by armed conflict.

Stage 2: Stratification of Facilities. The sampling frame was developed using the latest lists of functioning health facilities and registered nurses, obtained from the Ministry of Public Health and Population, complemented by the records of local health authorities and mapping by the humanitarian sector (e.g., Health Cluster Yemen). Facilities were deemed accessible based on security assessments carried out with local authorities and humanitarian partners; facilities that were under siege, fully closed, or posed imminent safety risks were excluded. Facilities were stratified according to ownership (public/private) and level of care (primary, secondary, tertiary).

Stage 3: Facility Selection. From the stratified sampling frame, 24 facilities were selected among the eight governorates. Within each governorate, facilities were randomly selected to achieve approximately 70% public and 30% private representation, reflecting the sector distribution. Three facilities were selected for each governorate, yielding a total of 24 facilities. This approach ensured representation of both the public and private sectors while remaining operationally feasible under security constraints.

Stage 4: Selection of Nurses in Facilities. Within each selected facility, systematic random sampling was used to select eligible nurses (every second eligible nurse was invited). Approximately equal participant allocation (approximately 100 nurses per governorate) was implemented to ensure adequate statistical power for governorate-level comparisons. This equal allocation was a pragmatic decision to ensure balanced representation across geographic regions, given that governorate-level nursing workforce sizes vary substantially.

The within-facility sampling fraction was approximately 0.50 (every second eligible nurse was invited). The overall response rate was 84.7% (800/944). Because equal allocation (~100 nurses per governorate) was used, selection probabilities differed across governorates. The primary analyses did not incorporate sampling weight; this is a limitation, and the implications for population-level estimates are discussed in the Limitations section. Sampling-weighted prevalence estimates are presented in Supplementary Table S11 (Supplementary File S4).

Sampling Table: A detailed sampling table showing, for each of the eight governorates, the number of eligible facilities in the sampling frame, public/private status, level of care, number selected, number of eligible nurses, number invited, number participating, and response rate is provided in Supplementary Table S9 (Supplementary File S4). Findings are generalizable only to accessible conflict-affected areas of Yemen.

#### 2.3.3. Response Rate and Replacements

Of the 872 eligible nurses approached through the initial systematic random sampling sequence, 72 did not participate: 42 (4.8%) declined, 18 (2.1%) were on emergency duty, and 12 (1.4%) were administratively unavailable. These 72 non-participants were replaced by the next available eligible nurses on the systematic random sampling list, resulting in 72 additional nurses being approached. The total number of nurses approached was therefore 944 (872 + 72), of whom 800 completed the questionnaire, yielding an overall response rate of 84.7% (800/944).

When a selected nurse was unavailable or declined, the next available eligible nurse on the systematic random sampling list was invited as a replacement. Replacement was incorporated into the sampling process by continuing the systematic random sampling sequence until the required sample size was achieved. Mental health outcomes were not available for non-participants, and non-response bias related to unmeasured factors cannot be excluded. This limitation is acknowledged in the Limitations section. Data were collected by trained local nurse researchers using approved security procedures. This pragmatic approach represented a trade-off between scientific rigor and operational feasibility and researcher safety. The final analytical sample comprised 800 nurses.

#### 2.3.4. Participant Selection Flow

**Figure 1:**
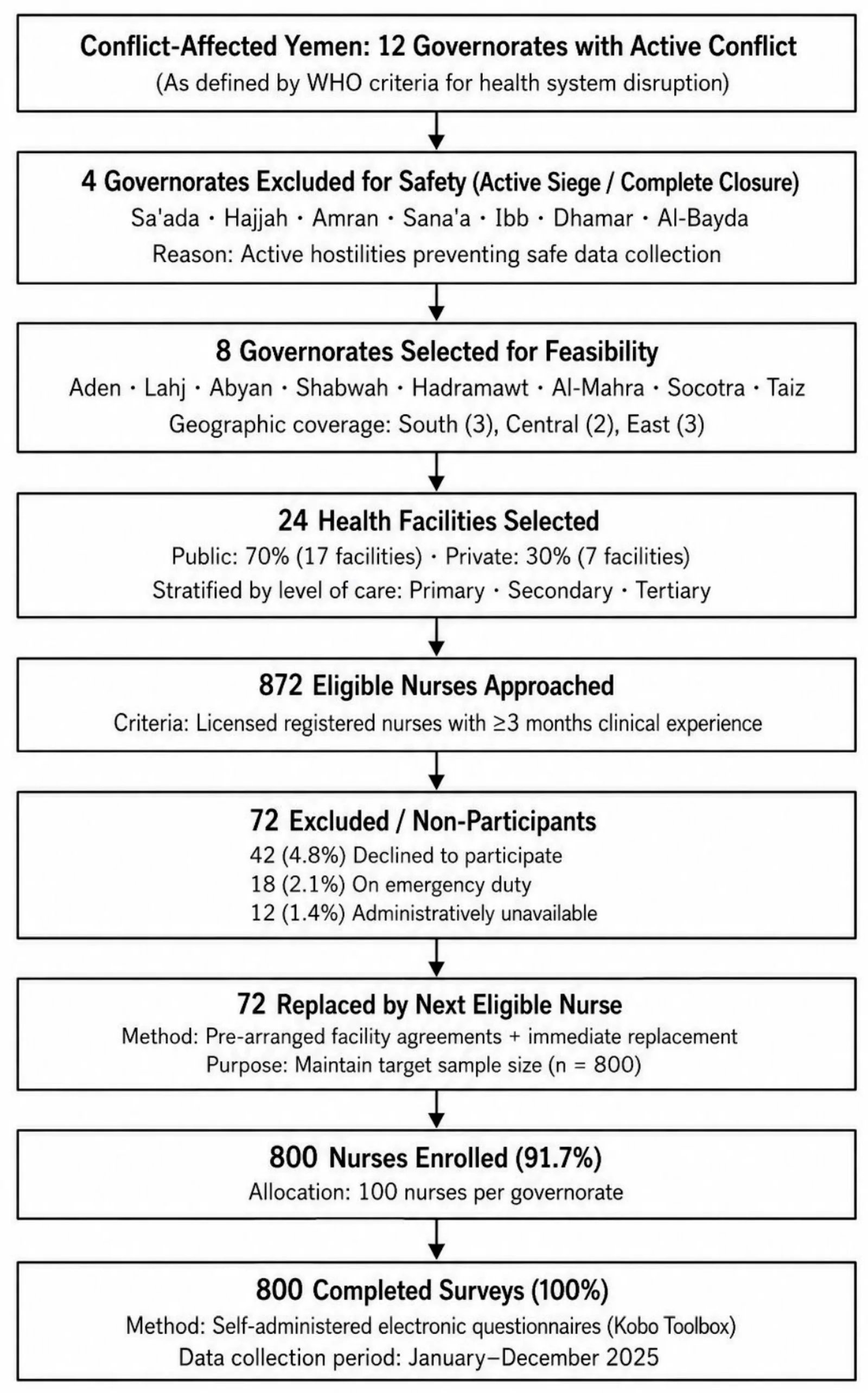
Flow Diagram of Participant Selection (STROBE).

Figure 1 displays the participant selection flow diagram, adapted from the STROBE guidelines, showing the number of nurses approached, excluded, and enrolled at each stage.

This STROBE-compliant flow diagram shows participant recruitment, eligibility screening, and final sample selection. Of 872 eligible nurses approached in the initial systematic random sampling sequence, 72 did not participate (42 [4.8%] declined, 18 [2.1%] were on emergency duty, and 12 [1.4%] were administratively unavailable) and were replaced by the next eligible nurses on the sampling list, resulting in 72 additional nurses being approached. The total number of nurses approached was therefore 944 (872 + 72), of whom 800 completed the questionnaire, yielding an overall response rate of 84.7% (800/944). The figure details the number of participants at each stage of the sampling process, including facility selection, nurse invitations, exclusions, and replacements.

## 3. Data Collection Tools and Measures

### 3.1. Main Outcome Measures

Depression symptoms: Depression symptoms were assessed using the validated Arabic version of the Patient Health Questionnaire-9 (PHQ-9), a 9-item questionnaire that assesses the presence of depressive symptoms over the past two weeks. Items are scored on a scale of 0 (not at all) to 3 (nearly every day). Total scores are categorized as minimal (0–4), mild (5–9), moderate (10–14), moderately severe (15–19), and severe (20–27). A cut-off score of ≥10 denoted clinically significant depressive symptoms. Supplementary File S2 contains the full PHQ-9 tool in Arabic and English, and scoring information is in Supplementary File S6 (Data Dictionary).

PHQ-9 Item 9 (Self-Harm) Safety Procedure: Item 9 of the PHQ-9 asks about “Thoughts of self-harm”. A particular safety procedure was established to address the endorsement of this item. A trained nurse researcher immediately performed an individual risk assessment for each non-zero response to Item 9 (e.g., “Several days”, “More than half the days”, or “Nearly every day”). The assessment systematically evaluated suicidal ideation, intent, plan, and resource availability. Individuals who were deemed to be in immediate danger were referred right away to the closest emergency room or mental health facility, or, if one was not available, to the on-site psychiatrist or mental health specialist. All participants who tested positive on Item 9 received a documented referral pathway that included emergency contacts and contact details for nearby mental health services. The study team adhered to a predetermined escalation process that was coordinated with local health authorities and humanitarian partners in conflict-affected situations when quick referral was impractical. Prior to data collection, the Institutional Review Board (IRB) examined and approved all safety protocols (IRB Reference Number: YEM-IRB-2025-094, Date: 15 January 2025). Throughout the trial period, no adverse events pertaining to the safety procedure were documented.

Anxiety symptoms: Anxiety symptoms were assessed with the Generalized Anxiety Disorder-7 (GAD-7) scale, which has been validated for Arabic. The 7-item questionnaire evaluates anxiety symptoms, with values varying from 0 to 21. Severity classifications include minimum (0–4), mild (5–9), moderate (10–14), and severe (15–21). A score of 10 or higher signifies moderate to severe anxiety. Information regarding the GAD-7 instrument and its validation can be found in Supplementary File S2.

Justification for primary outcomes: We selected depressive and anxiety symptoms as primary outcomes because they are among the most commonly reported and policy-relevant measures of workforce mental health and are directly associated with workforce retention, absenteeism, and quality of care [4,5]. The PHQ-9 and GAD-7 were selected because they are short (9 and 7 items, respectively), validated in Arabic and used in humanitarian settings, and therefore could be directly compared with nursing data from around the world.

### 3.2. Secondary Outcome Variables

Burnout Assessment: The evaluation of burnout was conducted using a singular query: “How do you evaluate your degree of burnout?” The research tool (Supplementary File S2) offered five distinct answer categories: None (0), Low (1), Moderate (2), High (3), and Severe (4). This practical approach was used to alleviate the strain on respondents within a highly pressured demographic.

Burnout Validation: To validate it in our setting, we conducted a pilot study of 40 nurses recruited from two facilities in Aden between 16–19 January 2025 (after getting IRB approval on 15 January 2025; before the main data collection). In this pilot, participants completed the MBI-Human Services Survey (MBI-HSS) emotional exhaustion subscale (Arabic version) simultaneously. The correlation between the single-item measure and the MBI emotional exhaustion subscale was estimated to use Pearson’s correlation coefficient after confirming that both variables followed a normal distribution (Shapiro–Wilk *p* > 0.05). The correlation was r = 0.82 (95% CI: 0.71–0.89, *p* < 0.001). This validation was done with the five response categories. This was an original validation performed solely for the present study. This gives us confidence in its use as a proxy for overall severity of burnout, but we acknowledge that it lacks the granularity to assess the multidimensionality of burnout (emotional exhaustion, depersonalization, and reduced personal accomplishment). Therefore, the results regarding burnout, particularly the additional analyses, should be interpreted with caution and validated in future research using established multidimensional instruments.

Burnout Coding for Analyses: For analyses, the original five response categories were collapsed into three categories as follows: None and Low were combined into “Low” (coded 1); Moderate remained “Moderate” (coded 2); High and Severe were combined into “High” (coded 3). This three-category coding was prespecified in the study protocol (Supplementary File S1) before analyses. “High burnout” was defined as a response of “high” or “severe” on the original five-point scale (i.e., Burnout Level = 3 in the collapsed coding). The numerator for the reported 34.0% prevalence was the number of participants who responded “High” or “Severe” (n = 272). This same coding was used consistently in the ordinal logistic regression, the turnover intention model, and the SEM analyses. The data dictionary (Supplementary File S6), and all reported prevalence estimates use exactly this definition.

Intention to Leave: Intention to leave was measured with the question, “Do you intend to leave your current nursing position within the next 12 months?” with a dichotomous (Yes/No) response. Single-item measures of turnover are common in nursing workforce research and possess predictive validity for actual turnover [14], but we recognize that multi-item scales (e.g., the Turnover Intention Scale [TIS-6] or the Michigan Organizational Assessment Questionnaire) provide a more complex measurement of turnover cognitions, including thoughts of quitting, intention to search for alternatives, and intention to leave the profession. The decision to use a single item was pragmatic, designed to minimize survey burden in this stressed population and maximize response rates in a challenging field setting. The discussion addresses this limitation. This item and all other survey questions are available on Supplementary File S2.

### 3.3. Independent Variables

Sociodemographic and occupational domains were used to group explanatory variables. Age (continuous), sex, marital status, and education (diploma, bachelor, master) were sociodemographic variables. The occupational characteristics were years of experience (continuous), type of employment (permanent/contract), type of facility (public/private), hours worked per shift, and night shifts per month.

Nurse-to-Patient Ratio: The workload was measured by the nurse-to-patient ratio, which was categorized into four groups for regression analyses: 1:10 (reference), 1:15, 1:20, and 1:25. For descriptive purposes, these categories were collapsed into two groups: ≤1:15 and ≥1:20, to provide a parsimonious summary of workload distribution. The four-category classification was used in all regression models and supplementary analyses, whereas the two-category classification was used only for descriptive presentation. This distinction is clearly indicated throughout the manuscript and supplementary materials. The rationale for using four categories in regression models was to examine dose–response relationships across the full range of nurse-to-patient ratios observed in the sample. These variables were chosen due to evidence that employment conditions, workload, and workplace context are associated with workforce retention.

### 3.4. Conflict and Exposure at Work

Workplace violence was dichotomized (exposed/not exposed) and defined as any physical, verbal, or psychological aggression from patients, relatives, or colleagues in the previous six months. Participants could report different types of violence (physical, verbal, and psychological) and different sources (patients, relatives, and colleagues). For the primary analyses, any exposure to any type from any source was coded as ‘exposed’. The types and sources of violence are listed separately for descriptive purposes (Supplementary Table S6 in Supplementary File S4). The percentages for types and sources of workplace violence are based on the 512 nurses who reported exposure to violence (not the total sample), and the distinction is clearly indicated in Supplementary Table S6. The Limitations section addresses the limitation that dichotomous coding fails to reflect the frequency, severity, or chronicity of exposure.

Displacement Status: Displacement status was measured with one question, “Are you currently displaced because of the conflict?” (Yes/No). Those who answered “Yes” were coded as displaced participants.

Monthly income adequacy was measured using a single question: “Is your monthly income adequate to cover your living expenses?” (Enough/Not enough). This was a self-rated measure based on participants’ perception of their financial situation relative to local living costs.

Psychological Support: Researchers measured the presence of workplace psychological support services using a single item: “Are mental health support services (such as counselling, peer support, or referral pathways) available at your workplace?” (No/Yes). This broad definition was deliberately inclusive, but we acknowledge that a dichotomous assessment does not reflect type, frequency, intensity, quality, or actual use of services. We cannot determine whether the observed association is due to access to support, the use of support, or other unmeasured factors, such as organizational culture. The Limitations section acknowledges this limitation.

### 3.5. Tool Validation and Adaptation

Instruments were carefully translated and culturally adapted. The forward and back translation was performed by bilingual nursing and psychology specialists. A panel of five experts (psychiatrists, nurse researchers, and conflict health specialists) assessed the content validity, with a Content Validity Index (CVI) ≥ 0.80. The pilot test with 40 nurses (not included in the final sample) showed satisfactory internal consistency (PHQ-9 α = 0.87; GAD-7 α = 0.85). Cognitive interviews verified cultural appropriateness and understanding. Full validation documentation and translated instruments are available in Supplementary File S2.

### 3.6. Procedures of Data Collection

Data were collected through structured, self-administered electronic questionnaires on encrypted tablets with offline functionality (Kobo Toolbox), which minimized data entry errors and facilitated real-time quality checks of the data. Bilingual, master’s-level nurse researchers, trained and familiar with the local context and security protocols, underwent a 5-day training on ethical data collection, respondent support, and distress protocol activation. Facility administrators supported survey administration by providing access to secure locations during daytime hours.

Of the 872 eligible nurses approached through the initial systematic random sampling sequence, 72 did not participate: 42 (4.8%) refused, 18 (2.1%) were on emergency duty, and 12 (1.4%) were unavailable for administrative reasons. These 72 non-participants were replaced with the next eligible nurses on the sampling list, resulting in 72 additional nurses being approached. The total number approached was 944 (872 + 72), of whom 800 participated, yielding an overall response rate of 84.7% (800/944). The final analytical sample comprised 800 nurses.

Replacement was facilitated by (1) prearranged facility agreements, (2) data collection during non-peak hours, and (3) immediate replacement. The strategy was developed to minimize disturbance to the sampling frame.

### 3.7. Quality Assurance Measures

To guarantee data quality, several quality assurance measures were implemented, including daily random verification of 10% of completed questionnaires, automated range and consistency checks embedded in the data collection platform, weekly review meetings to address field challenges, and secure cloud-based data backup with encrypted transmission.

### 3.8. Respondent Support Protocol

All participants were provided with information about local mental health support services. If the PHQ-9 or GAD-7 scores were ≥15, the nurses were immediately offered referral to a collaborating mental health professional.

### 3.9. Ethical Consideration

The study was approved by the Institutional Review Board of Radfan College University, Yemen (YEM-IRB-2025-094) and conducted in line with the Declaration of Helsinki. All participants provided written informed consent and were informed of their right to withdraw at any time. Arabic and English versions of consent forms are provided (Supplementary File S2). To ensure confidentiality, all data were anonymized at the point of collection and stored on encrypted password-protected servers. During fieldwork, the research team was supported psychologically and in terms of safety, including training in psychological first aid, regular debriefing sessions, mental health support, and security protocols appropriate for conflict settings.

### 3.10. Statistical Analyses

Statistical analyses were performed using R version 4.3.1 (R Foundation for Statistical Computing, Vienna, Austria). Statistical significance was set at *p* < 0.05. Participant characteristics were summarized using descriptive statistics, and bivariate analyses were performed using chi-square, Fisher’s exact, *t*-tests, or Mann–Whitney tests as appropriate. Multivariable mixed-effects logistic regression models with facility-level random intercepts were used to identify factors associated with depression, anxiety, and turnover intention. Cluster-robust standard errors were applied to account for clustering at the facility level [15]. Burnout was analyzed with mixed-effects ordinal logistic regression with facility-level random intercepts. Continuous PHQ-9 and GAD-7 scores were analyzed with mixed-effects linear regression with facility-level random intercepts. Model building was based on a pre-specified JD-R framework described in the study protocol (Supplementary File S1).

Model diagnostics for multicollinearity were assessed using variance inflation factors (VIF) with values >5 indicating problematic collinearity. The discrimination was measured by the area under the receiver operating characteristic curve (AUC). Calibration was assessed by calibration plots and Brier scores. Overall model goodness-of-fit was assessed using Akaike Information Criterion (AIC) and Hosmer–Lemeshow tests. The influence diagnostics used were Cook’s distance and DFBETAS [16]. Internal validation and reduction in the optimistic bias were done by using 10-fold cross-validation to calculate the discrimination and calibration metrics.

Model Specification: Turnover Intention. For the turnover intention outcome, we present two models in Table 4 (main text). Model 1: Associations between occupational/conflict exposures and turnover intention, not adjusted for mental health variables (depression, anxiety, burnout), estimating overall associations. Conditional on psychological distress (adjusting for depression, anxiety, and burnout): Model 2 (Supplementary Table S2c, Supplementary File S4). This distinction is important because mental health variables could be on the same conceptual pathway as turnover intention, and their inclusion in the model could substantially alter the association between exposures and turnover intention. The models are presented in both cases to make their interpretation easier.

### 3.11. Additional and Sensitivity Analyses

Exploratory Structural Equation Modelling: Exploratory structural equation modelling (SEM) was conducted to investigate potential relationships among workplace stressors, burnout, and psychological distress. The model was estimated using robust maximum likelihood (MLR) for continuous outcomes and weighted least squares mean and variance adjusted (WLSMV) for binary outcomes. Model fit was evaluated using the comparative fit index (CFI), Tucker–Lewis index (TLI), root mean square error of approximation (RMSEA), and standardized root mean square residual (SRMR), with acceptable fit defined as CFI ≥ 0.90, TLI ≥ 0.90, RMSEA ≤ 0.08, and SRMR ≤ 0.08. All details are given in Supplementary File S5 (Supplementary Figure S1). These analyses should be considered hypothesis-generating due to the cross-sectional design and single-item burnout measure.

Sensitivity analyses of robustness were tested using sensitivity analyses including multiple imputation for missing data (<3%), alternative cut-offs for PHQ-9 and GAD-7, and subgroup analyses (Supplementary File S4). To supplement odds ratios, we estimated risk ratios using Poisson regression with robust variance because depressive and anxiety symptoms were common outcomes (Supplementary Table S2b in Supplementary File S4) [17,18]. All primary analyses were pre-specified (Supplementary File S1). Exploratory analyses (SEM, exploratory indirect associations, effect modification, and interaction) were prespecified as exploratory in the protocol and are presented as hypothesis-generating.

Missing Data: The extent and pattern of missing data for the main variables was as follows: PHQ-9 (1.2% missing), GAD-7 (1.5% missing), burnout (2.1% missing), turnover intention (0.9% missing), and the main exposures (displacement 1.8%, workplace violence 2.3%, income adequacy 2.0%, and nurse-to-patient ratio 1.6%). Missing data were addressed using multiple imputation by chained equations (MICE), with 20 imputations. The imputation model included all principal variables, auxiliary variables (age, gender, and type of facility), and the outcome variables. A complete-case analyses was also conducted and compared to the imputed results.

### 3.12. Standards for Reporting

Reporting checklists completed are available in Supplementary File S3 (STROBE, SAGER, Data Quality Assurance, and Conflict Research Ethics checklists).

## 4. Results

### 4.1. Study Population Characteristics

The study enrolled 800 nurses from 24 health facilities across eight conflict-affected governorates (overall response rate: 84.7%, 800/944). The mean age was 38.7 years (SD = 10.2), with 54.5% female participants. The majority were married (56.5%), and educational qualifications included diploma (42.8%), bachelor’s (42.0%), and master’s (15.3%). The mean experience was 15.3 years (SD = 9.8), with 51.5% employed on a contract basis. Over half were displaced (54.0%), reported inadequate income (56.5%), and had experienced workplace violence in the past 6 months (64.0%). Most worked in public facilities (52.0%), with 44.0% reporting nurse-to-patient ratios of ≥1:20. Complete demographic and exposure characteristics are presented in Table 1.

The characteristics and geographic distribution of participating governorates, including the number of facilities, participants, response rates, and security level assessments, are presented in Table 2.

Among the 512 nurses (64.0%) who reported exposure to workplace violence, the majority experienced verbal aggression (55.5%), followed by psychological aggression (25.0%). Patients were the most common source of violence (71.9%), and notably, only 12.5% of affected nurses reported incidents to authorities (Supplementary Table S6, Supplementary File S4). This alarmingly low reporting rate suggests a culture of underreporting that may reflect the normalization of violence, fear of retaliation, or lack of confidential reporting mechanisms in conflict-affected health settings.

Of the 72 nurses who did not participate in the initial sampling sequence, mental health outcomes and other key variables were not available, and therefore a formal comparison between participants and non-participants could not be performed. Non-response bias related to unmeasured factors, including mental health status, cannot be excluded. This limitation is acknowledged in the Limitations section.

Several predictors showed no statistically significant associations across adjusted models. For example, nurse–patient ratios of 1:15 did not reach significance for any outcome (depression AOR = 1.30, *p* = 0.226; anxiety AOR = 1.28, *p* = 0.247; turnover AOR = 1.22, *p* = 0.358), and age was borderline across outcomes (*p* ≈ 0.051). Employment type and facility type showed no consistent associations. These non-significant findings are addressed in the discussion, and sensitivity analyses and are summarized in Supplementary Table S10 (Supplementary File S4).

To ensure adequate statistical power for governorate-level comparisons, approximately equal participant allocation was implemented across selected governorates.

### 4.2. Prevalence of Psychological Distress Outcomes

**Figure 2:**
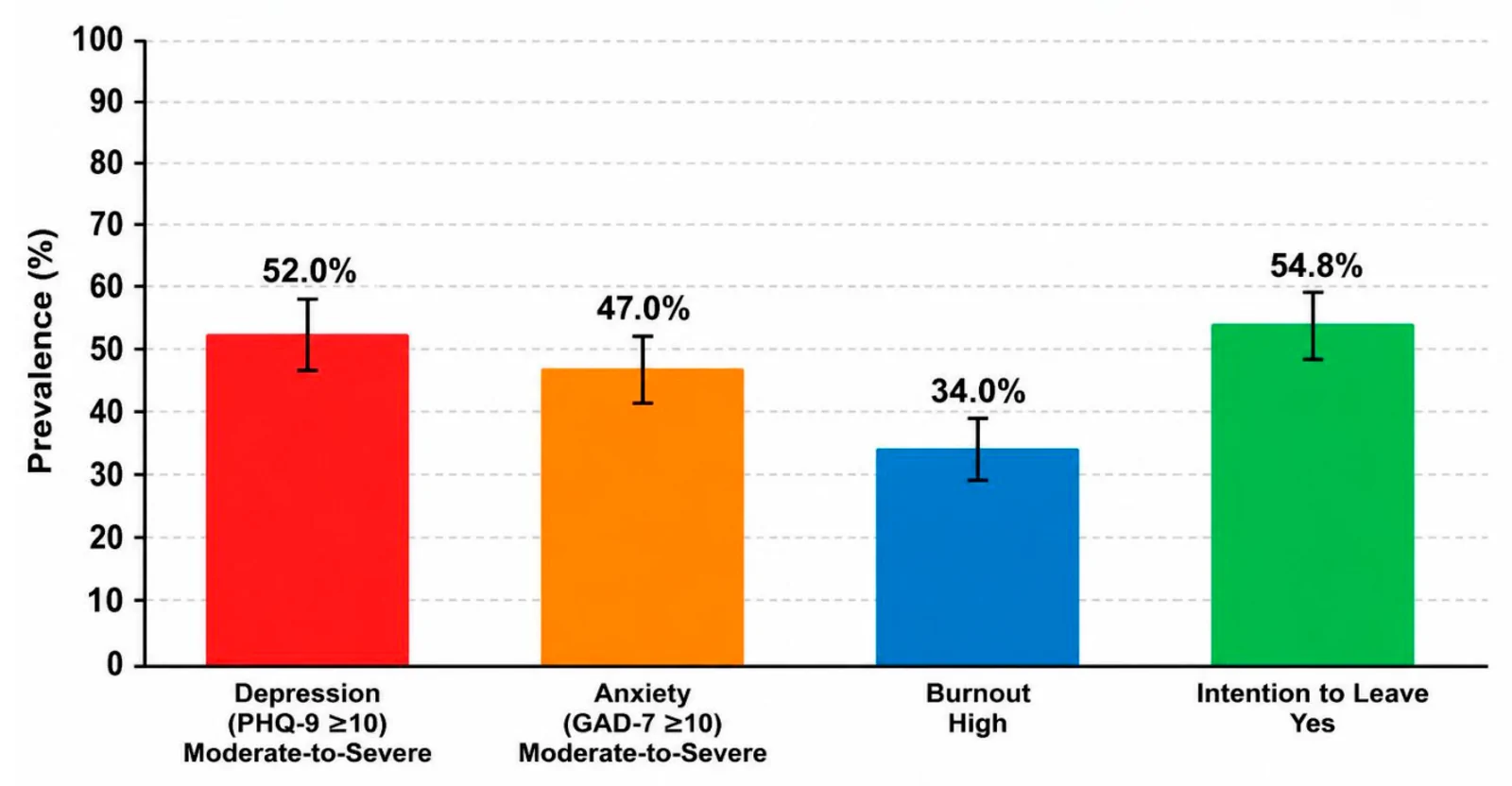
Prevalence of Psychological Distress Outcomes. Prevalence of Moderate-to-Severe Depression, Anxiety, High Burnout, and Turnover Intention Among Nurses (N = 800). This figure displays the prevalence of moderate-to-severe depression (PHQ-9 ≥ 10), moderate-to-severe anxiety (GAD-7 ≥ 10), high burnout, and intention to leave within 12 months among 800 nurses in conflict-affected Yemen. Error bars represent 95% confidence intervals.

Table 3 presents the prevalence of psychological distress and occupational outcomes. Over half (52.0%, 95% CI: 48.5–55.5) of nurses screened positive for clinically significant depression (PHQ-9 ≥ 10), with 10.0% reporting severe symptoms (PHQ-9 ≥ 20). Nearly half (47.0%, 95% CI: 43.5–50.5) reported moderate-to-severe anxiety (GAD-7 ≥ 10). High self-rated burnout was reported by 34.0%, and more than half (54.8%, 95% CI: 51.2–58.3) expressed an intention to leave their current position within 12 months (Figure 2).

Sampling-weighted prevalence estimates were calculated using inverse-probability weights based on governorate-level nursing workforce sizes obtained from the Ministry of Public Health and Population and Health Cluster Yemen (Supplementary Table S11, Supplementary File S4). The weighted prevalence was 52.4% (95% CI: 48.9–55.9) for moderate-to-severe depressive symptoms, 47.5% (95% CI: 44.0–51.0) for moderate-to-severe anxiety symptoms, 33.6% (95% CI: 30.3–36.9) for high self-rated burnout, and 55.1% (95% CI: 51.5–58.7) for turnover intention. These weighted estimates were nearly identical to the unweighted estimates (absolute differences < 1 percentage point), indicating that differential selection probabilities across governorates did not materially affect the prevalence estimates. For population-level interpretation, the weighted estimates should be preferred; however, the close agreement between weighted and unweighted estimates strengthens confidence in the findings.

Additional details on workplace violence characteristics are provided in Supplementary Table S4.

## 5. Multivariable Regression Analyses

### 5.1. Factors Associated with Psychological Distress

Table 4 presents final multivariable models for depression, anxiety, and turnover intention, all accounting for facility-level clustering. ICC values for null models were modest (depression = 0.08, 95% CI: 0.03–0.18; anxiety = 0.07, 95% CI: 0.02–0.16; burnout = 0.09, 95% CI: 0.04–0.19; turnover = 0.06, 95% CI: 0.02–0.14), appropriately addressed through mixed-effects models.

Conflict-related exposures demonstrated the largest adjusted effect estimates in multivariable models. Displacement (AOR = 2.31, 95% CI: 1.68–3.17), inadequate income (AOR = 2.53, 95% CI: 1.80–3.56), and workplace violence (AOR = 2.84, 95% CI: 2.03–3.97) were associated with 2.3–2.8 times higher odds of depression, with similar patterns for anxiety (AOR = 2.18, 2.41, and 2.67, respectively).

Adverse working conditions showed dose–response relationships. Each additional night shift increased depression odds by 26% (AOR = 1.26, 95% CI: 1.17–1.36) and anxiety by 24% (AOR = 1.24, 95% CI: 1.16–1.33). Compared to 1:10 ratios, 1:20 ratios were associated with ~1.7 times higher odds of depression (AOR = 1.68, 95% CI: 1.07–2.63) and anxiety (AOR = 1.62, 95% CI: 1.05–2.51), while 1:25 ratios showed ~2.9 times higher odds (depression AOR = 2.94, 95% CI: 1.80–4.80; anxiety AOR = 2.88, 95% CI: 1.81–4.59). Lack of psychological support was significantly associated with increased depression (AOR = 1.82, 95% CI: 1.30–2.54) and anxiety (AOR = 1.76, 95% CI: 1.28–2.41).

### 5.2. Interpretation of Effect Sizes

As the prevalence of depressive symptoms (52.0%) and anxiety symptoms (47.0%) is high, odds ratios can overestimate associations when the outcomes are common [17,19]. To facilitate interpretation, we estimated predicted probabilities and risk ratios (RR) using Poisson regression with robust variance [18]. Poisson regression with robust variance was preferred to log-binomial regression because log-binomial models sometimes do not converge when the outcome is common, whereas Poisson with robust variance produces consistent and conservative estimates of RR [18]. Predicted probabilities were estimated from the mixed-effects logistic regression models using marginal standardization, with all other covariates fixed at their means. Risk differences were estimated as the difference in predicted probabilities between exposed and unexposed participants. Supplementary Table S2a (Supplementary File S4) presents the predicted probabilities, risk differences, and number needed to harm (NNH) for the key conflict-related exposures.

The AORs of 2.84 and 2.29 for workplace violence and displacement, respectively, correspond to an increase of 25 and 20 percentage points in the probability of depressive symptoms (from 31% to 56% and from 36% to 56%, respectively). Risk ratios (Supplementary Table S2b, Supplementary File S4) supported clinical significance with more conservative estimates (e.g., RR = 1.72 for workplace violence; RR = 1.55 for displacement). We prefer RRs for clinical and policy interpretation because they give more conservative and interpretable estimates where outcomes are common. RRs are thus reported together with AORs in the main text for important associations, with AORs kept for comparison with the existing literature. For clinical and policy interpretation, the RR or the absolute risk difference should be the main effect measure, not the AOR.

Importantly, effect sizes for some predictors, though statistically significant, were of smaller magnitude than those seen for conflict-related exposures. Each additional night shift was associated with a 26% increase in odds of depressive symptoms (AOR = 1.26). Thus, while shift scheduling is important, its impact is less than that of structural and security-related factors. This pattern is consistent with findings from other conflict and humanitarian settings in that the association of psychological distress with structural and security-related factors such as displacement, insecurity, and exposure to violence is greater than that with occupation-specific factors [8,12].

### 5.3. Turnover Intention

**Figure 3:**
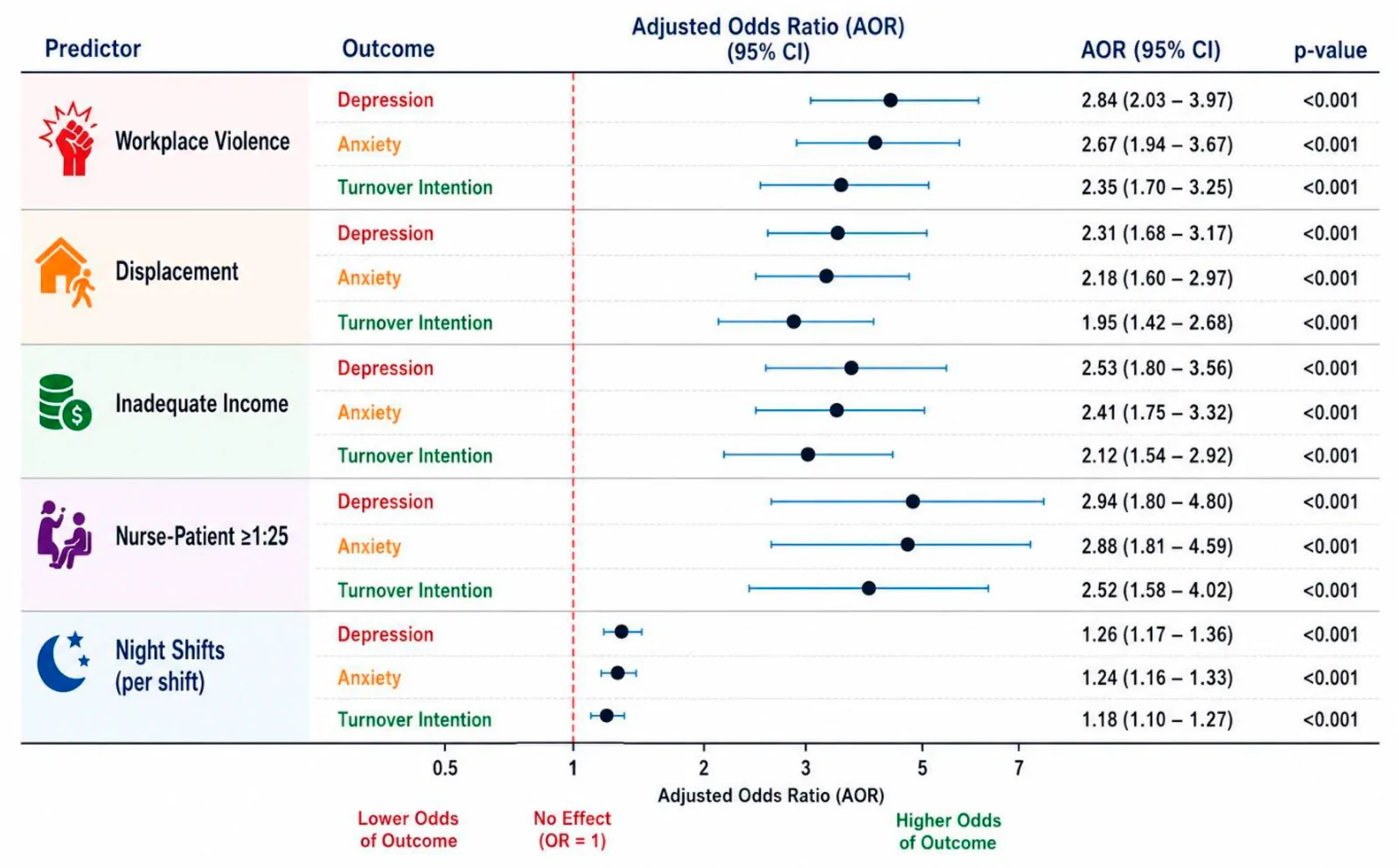
Adjusted Odds Ratios for Key Predictors of Depressive Symptoms. This forest plot displays the adjusted odds ratios (AOR) for key predictors of moderate-to-severe depressive symptoms (PHQ-9 ≥ 10) among 800 nurses in conflict-affected Yemen. All models were adjusted for age, gender, employment type, and facility-level clustering. Error bars represent 95% confidence intervals. The dashed vertical line at AOR = 1.0 indicates no effect.

For turnover intention, the strongest predictors were mental health variables: high burnout (AOR = 4.15, 95% CI: 2.97–5.80), moderate-to-severe depression (AOR = 3.42, 95% CI: 2.48–4.71), and anxiety (AOR = 2.88, 95% CI: 2.10–3.95) (Figure 3). Conflict-related exposures and working conditions remained significant but showed weaker direct associations after adjusting for mental health, consistent with strong associations between occupational exposures, psychological distress, and turnover intention (Table 4).

To further explore the determinants of burnout, we conducted an ordinal logistic regression model examining burnout severity across its three levels (low, moderate, and high). This analysis (Supplementary Table S1) demonstrated similar patterns of association as observed for depression and anxiety, with displacement (AOR = 2.15), inadequate income (AOR = 2.38), and workplace violence (AOR = 2.72) showing strong dose–response relationships with increasing burnout severity. These findings reinforce the cumulative nature of occupational and conflict-related stressors in shaping burnout among nurses in conflict-affected settings.

**Table 4:** Multivariable Associations with Depressive Symptoms, Anxiety Symptoms, and Turnover Intention (N = 800).

Predictor	Depressive Symptoms (PHQ-9 ≥ 10)			Anxiety Symptoms (GAD-7 ≥ 10)			Intention to Leave (Model 1)		
	AOR (95% CI)	*p*	RR (95% CI)	AOR (95% CI)	*p*	RR (95% CI)	AOR (95% CI)	*p*	RR (95% CI)
**Sociodemographic Factors**									
Age (per year)	0.98 (0.96–1.00)	0.051 †	0.998 (0.994–1.002)	0.99 (0.97–1.00)	0.051 †	0.998 (0.995–1.002)	0.99 (0.97–1.00)	0.051 †	0.999 (0.996–1.002)
Female gender (Ref: Male)	1.32 (0.95–1.83)	0.095	1.09 (0.93–1.28)	1.25 (0.92–1.70)	0.162	1.07 (0.91–1.25)	1.18 (0.86–1.62)	0.301	1.05 (0.91–1.22)
**Occupational Factors**									
Contract employment (Ref: Permanent)	1.29 (0.94–1.77)	0.117	1.10 (0.94–1.29)	1.18 (0.87–1.60)	0.295	1.06 (0.92–1.23)	1.22 (0.89–1.67)	0.214	1.08 (0.94–1.25)
Night shifts per month (each)	1.26 (1.17–1.36)	<0.001	1.09 (1.06–1.12)	1.24 (1.16–1.33)	<0.001	1.08 (1.05–1.11)	1.18 (1.10–1.27)	<0.001	1.06 (1.03–1.09)
**Nurse–patient ratio (Ref: 1:10)**									
1:15	1.30 (0.85–2.00)	0.226	1.13 (0.87–1.47)	1.28 (0.84–1.95)	0.247	1.12 (0.87–1.44)	1.22 (0.80–1.86)	0.358	1.10 (0.86–1.41)
1:20	1.68 (1.07–2.63)	0.024	1.32 (1.12–1.56)	1.62 (1.05–2.51)	0.029	1.28 (1.09–1.50)	1.62 (1.18–2.22)	0.003	1.35 (1.14–1.60)
1:25	2.94 (1.80–4.80)	<0.001	1.79 (1.44–2.22)	2.88 (1.81–4.59)	<0.001	1.75 (1.43–2.14)	2.52 (1.58–4.02)	<0.001	1.60 (1.32–1.94)
**Conflict & Workplace Exposures**									
Displaced (Ref: Non-displaced)	2.31 (1.68–3.17)	<0.001	1.55 (1.32–1.82)	2.18 (1.60–2.97)	<0.001	1.48 (1.27–1.73)	1.95 (1.42–2.68)	<0.001	1.42 (1.23–1.64)
Inadequate income (Ref: Adequate)	2.53 (1.80–3.56)	<0.001	1.61 (1.36–1.91)	2.41 (1.75–3.32)	<0.001	1.57 (1.33–1.85)	2.12 (1.54–2.92)	<0.001	1.48 (1.28–1.71)
Workplace violence exposure (Ref: No)	2.84 (2.03–3.97)	<0.001	1.72 (1.45–2.04)	2.67 (1.94–3.67)	<0.001	1.65 (1.40–1.95)	2.35 (1.70–3.25)	<0.001	1.55 (1.33–1.81)
No psychological support (Ref: Available)	1.82 (1.30–2.54)	0.001	1.41 (1.20–1.66)	1.76 (1.28–2.41)	0.001	1.38 (1.18–1.61)	1.58 (1.15–2.17)	0.005	1.32 (1.14–1.53)

**Notes: Abbreviations:** AOR = Adjusted Odds Ratio; CI = Confidence Interval; RR = Risk Ratio. **Model Specification:** All models are mixed-effects logistic regression models with facility-level random intercepts and cluster-robust standard errors to account for clustering. **Risk Ratios:** Risk ratios were estimated using Poisson regression with robust variance [18] because depressive and anxiety symptoms were common outcomes [17,19]. For clinical and policy interpretation, RRs are preferred, as they provide more conservative estimates when outcomes are common. **Statistical Significance:** Statistically significant associations (*p* < 0.05) are presented in bold with 95% CIs excluding 1.00. **Model Performance:** Model performance is indicated by Nagelkerke’s pseudo R^2^ and the Akaike Information Criterion (AIC). **Intention to Leave Model:** The “Intention to Leave” model presented here (Model 1) does not adjust for mental health status variables (depressive symptoms, anxiety symptoms, and burnout), estimating overall associations. A model adjusting for these variables (Model 2) is presented in Supplementary Table S2c (Supplementary File S4). This distinction allows for interpretation of overall associations (Model 1) versus associations conditional on psychological distress (Model 2). **Not Included:** “—“ indicates a variable not included in that model. **Age (per year):** † For age, *p* = 0.051 across all three models, approaching but not reaching the conventional threshold of 0.05. **Interpretation:** For example, workplace violence exposure was associated with an adjusted odds ratio of 2.84 for depressive symptoms (AOR = 2.84, 95% CI: 2.03–3.97) and a risk ratio of 1.72 (RR = 1.72, 95% CI: 1.45–2.04), corresponding to a 25 percentage-point increase in depressive symptom probability (from 31% to 56%; see Supplementary Table S2a in Supplementary File S4).

### 5.4. Model Performance and Discrimination

All multivariable models demonstrated good discriminatory ability. The AUC values were 0.82 (95% CI: 0.79–0.85) for the depression model, 0.79 (95% CI: 0.76–0.82) for the anxiety model, and 0.81 (95% CI: 0.78–0.84) for the turnover intention model. Calibration plots showed adequate fit for all models, with Brier scores of 0.18 for depression, 0.21 for anxiety, and 0.19 for turnover intention. These metrics were calculated using 10-fold cross-validation to provide internal validation and reduce optimistic bias. Apparent performance (calculated on the same data used to fit the models) was slightly higher, as expected, with AUC values of 0.84, 0.81, and 0.83 for depression, anxiety, and turnover intention, respectively. The cross-validated metrics are reported here as more conservative estimates of model performance.

### 5.5. Linear Regression for Continuous Symptom Scores

The correlation between PHQ-9 and GAD-7 scores was moderate (r = 0.58, *p* < 0.001), indicating that while depression and anxiety frequently co-occur, they represent distinct constructs. Linear regression confirmed significant associations with continuous PHQ-9 and GAD-7 scores (Supplementary Table S2). Workplace violence was associated with a 3.15-point increase in PHQ-9 (95% CI: 2.40–3.90) and a 2.88-point increase in GAD-7 (95% CI: 2.20–3.56). Nurse–patient ratios of 1:25 were associated with nearly 3-point higher depression scores compared to 1:10 (Supplementary Table S2).

### 5.6. Supplementary Analyses: Exploratory Pathway, Effect Modification, and Interaction Effects

All mediation and moderation analyses were pre-specified as exploratory and are presented in the supplementary materials (Supplementary File S5, Sections S1 and S2, and Supplementary Figure S1). In brief, further exploratory analyses revealed associations between burnout and several occupational and psychological outcomes. The SEM model was an acceptable fit (CFI = 0.92, TLI = 0.90, RMSEA = 0.06 [90% CI: 0.04–0.08], SRMR = 0.05) and met conventional thresholds for acceptable fit (CFI ≥ 0.90, TLI ≥ 0.90, RMSEA ≤ 0.08, SRMR ≤ 0.08). Given the cross-sectional design and the single-item burnout measure, these findings are strictly hypothesis-generating and require validation using multidimensional instruments in longitudinal studies.

**Figure 4:**
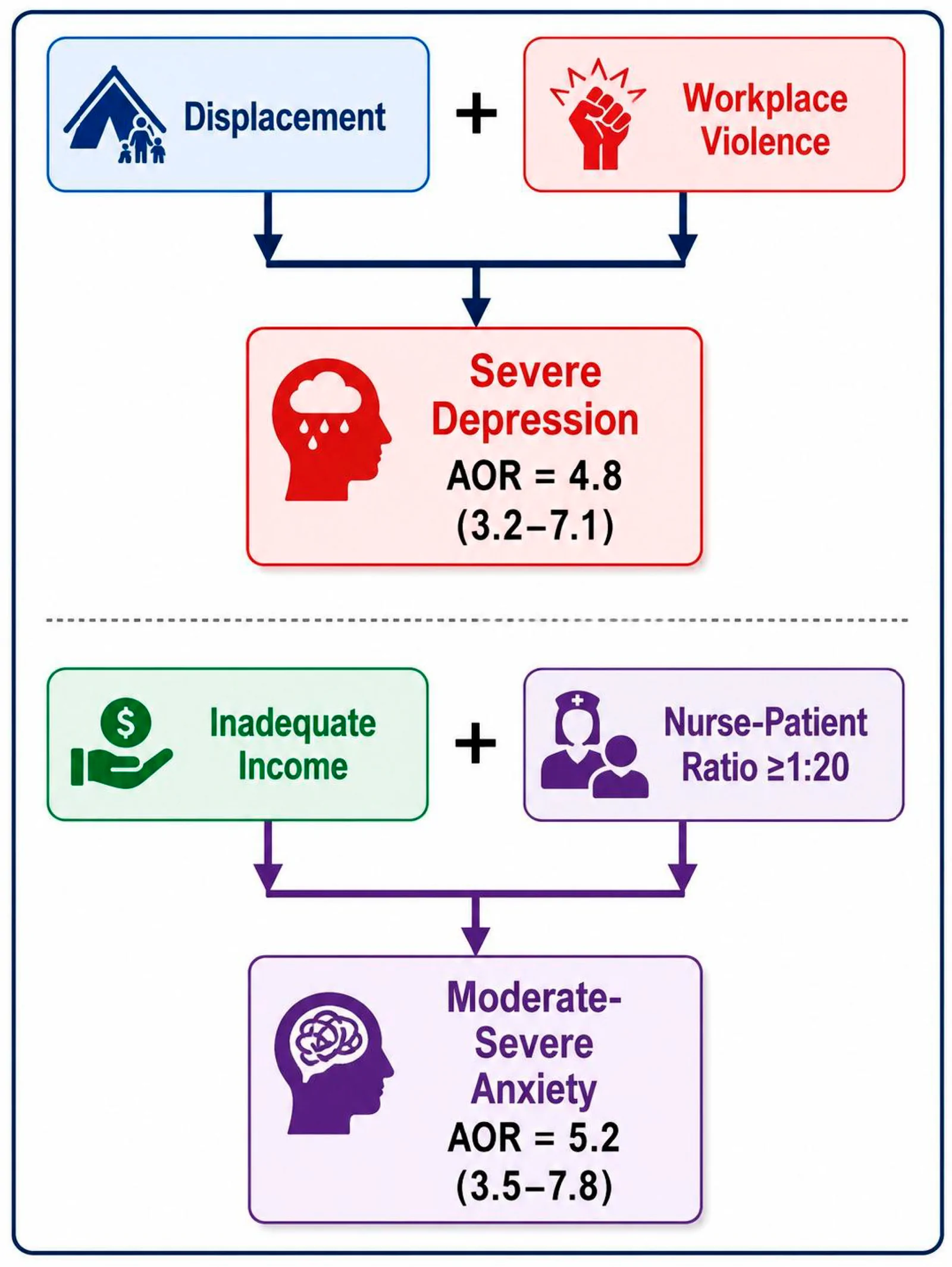
Interaction Effects of Combined Conflict-Related and Occupational Exposure on Severe Depressive Symptoms and Moderate-to-Severe Anxiety Symptoms. Nurses exposed to both displacement and workplace violence were 4.8 times more likely to have severe depressive symptoms (95% CI: 3.2–7.1) than those exposed to neither. Those who combined inadequate income and a high nurse–patient ratio (≥1:20) were 5.2 times more likely to have moderate-to-severe anxiety symptoms (95% CI: 3.5–7.8). These interactions are purely statistical and should not be interpreted as evidence of causal synergism.

Psychological support as an effect modifier: The association between exposure to conflict and depressive symptoms differed according to the reported availability of psychological support at work (β for interaction = −0.23, *p* < 0.001). Statistical indirect associations (e.g., burnout statistically accounted for 42.3% of the observed association between workplace violence and depressive symptoms and 38.7% of the association between displacement and anxiety symptoms) were compatible with the proposed model; however, these should not be interpreted as evidence of causal mediation. Displacement and workplace violence showed a statistical interaction for severe depressive symptoms (PHQ-9 ≥ 20) (AOR = 4.8, 95% CI: 3.2–7.1). Similarly, inadequate income and a nurse–patient ratio of ≥1:20 showed a statistical interaction for moderate-to-severe anxiety symptoms (GAD-7 ≥ 10) (AOR = 5.2, 95% CI: 3.5–7.8) (Figure 4). Details are presented in Supplementary File S5 (Sections S1 and S2, Supplementary Figure S1), including the full mediation model, moderation analyses, and interaction terms.

These findings are purely associational and hypothesis-generating. The cross-sectional design precludes causal interpretation, and the exploratory mediation analyses should not be interpreted as evidence of causal pathways.

### 5.7. Sensitivity Analyses

We performed several sensitivity analyses to test the robustness of our results, and the full results are shown in the supplementary materials (Supplementary File S4, Supplementary Tables S1–S11). To assess the possible impact of missing data, we conducted complete-case analyses and multiple imputations of missing data (<3%). We obtained very similar results, with absolute differences between 0.01 and 0.02 (Supplementary Table S1 in Supplementary File S4), confirming that missing data did not materially affect our results.

We also examined the robustness of our results by using alternative thresholds for depressive symptoms (PHQ-9 ≥ 5 and ≥15) and anxiety symptoms (GAD-7 ≥ 5 and ≥8). (Supplementary Table S3 in Supplementary File S4) These analyses confirmed that the key associations remained significant at all thresholds, although effect sizes were slightly attenuated at lower severity cut-offs, as expected. Checks for model specification showed no problematic multicollinearity (mean VIF = 1.26, all VIF < 5). Influence diagnostics showed that 18 observations (1.0%) had Cook’s distance values above the threshold of 0.005 and no observations had DFBETAS values above the threshold of 1.0. Leaving out these influential observations did not materially change the estimates.

To assess whether our findings were driven by extreme cases, we re-ran the main models excluding nurses with severe depressive symptoms (PHQ-9 ≥ 20) and severe anxiety symptoms (GAD-7 ≥ 15). The results (Supplementary Table S4 in Supplementary File S4) were consistent with the main analyses, indicating that outliers did not disproportionately influence our conclusions.

All continuous items (PHQ-9, GAD-7, and burnout items) were subjected to exploratory factor analyses to conduct Harman’s single factor test. The first unrotated factor explained 28.4% of variance (eigenvalue = 5.68). This result is presented in a descriptive way only and it does not rule out the possibility of common method variance, especially considering that the main exposures and outcomes were self-reported within the same questionnaire. The study design and the limits of measurement should be the primary safeguards against common method bias itself.

However, most associations were statistically significant; a few expected relationships were not universally supported. Nurse-to-patient ratios of 1:15 were not significant for any outcome (depressive symptom AOR = 1.30, *p* = 0.226; anxiety symptom AOR = 1.28, *p* = 0.247; turnover AOR = 1.22, *p* = 0.358). Age was borderline significant across outcomes (*p* = 0.051 for all three outcomes). No consistent associations were found for employment type and facility type. A full summary of all non-significant and borderline findings can be found in Supplementary Table S10 (Supplementary File S4).

Alternative specifications (excluding outliers, alternative cut-offs, multiple imputation) showed a consistent direction of associations, but the magnitude and significance varied for some predictors. In the main analyses, nurse–patient ratio ≥ 1:20 was significant for depressive symptoms (AOR = 1.68, *p* = 0.024), but borderline when nurses with severe depressive symptoms were excluded (AOR = 1.52, *p* = 0.068). Similarly, the relationship between displacement and anxiety symptoms was somewhat reduced with a lower cut-off (GAD-7 ≥ 5: AOR = 1.95, *p* < 0.001) compared with the main analyses (GAD-7 ≥ 10: AOR = 2.18, *p* < 0.001) but was still highly significant. This variation emphasizes robustness but also modest effect sizes.

The sensitivity analyses (Supplementary Tables S1 and S3–S11, Supplementary File S4) support the overall robustness of primary findings, while pointing to areas where effect sizes were more modest and larger samples may be needed to detect smaller effects in subgroup and interaction analyses.

## 6. Discussion

This study gives multicenter evidence on mental health, burnout and turnover intention among nurses working in eight accessible conflict-affected governorates of Yemen. We identified high prevalences of depression (52.0%), anxiety (47.0%), self-rated burnout (34.0%), and turnover intention (54.8%) among 800 nurses across eight accessible governorates, all of which are alarmingly higher than global estimates among nurses, demonstrating that nurses in conflict settings are under occupational stress and exposed to trauma. These results indicate that psychological morbidity is influenced by a range of cumulative occupational and conflict-related stressors including displacement, violence at work, inadequate income, high patient load, and frequent night shifts. This points out that mental health outcomes in humanitarian contexts are not only a matter of individual vulnerability but also shaped by structural factors.

### 6.1. Comparison with Previous Evidence

The rates of depression and anxiety reported are significantly higher than the global estimates for nursing staff (22–30% and 30–40%, respectively) [4,5], and similar to conflict-affected workforces in Syria and Afghanistan [20,21]. However, odds ratios can inflate the magnitude of associations because of the high prevalence of the outcome [17,22]. Absolute risk differences are more conservative estimates (e.g., workplace violence was associated with a 25 percentage-point increase in depression risk). Other studies have also reported high burnout among nurses in non-conflict settings in regions such as Nigeria, but the prevalence in our sample is much higher (34.0%), which highlights the additional burden of armed conflict [23].

Distress levels found in the present study were high and similar to those reported in some conflict-affected populations, supporting the ‘dual burden’ framework [12]. The high prevalence of displacement and workplace violence points to significant exposure to conflict-related stressors among participants. This is consistent with the hypothesis that chronic daily stressors, including financial precarity, excessive workload, and unsafe environments, are strongly associated with psychological distress, similar to associations reported for acute traumatic events.

Our results are consistent with the large body of evidence that workplace violence is associated with poor mental health [24]. In conflict-affected systems, violence is embedded in pervasive insecurity and institutional failure, normalizing threat as routine and removing protective buffers. Similarly, the association between high nurse–patient ratios and distress is consistent with evidence from high-income countries linking staffing shortages to burnout and turnover [25]. But in Yemen, these ratios are a serious staffing constraint for a decimated system, and the findings highlight workforce preservation as an important concern alongside workload management in a time of systemic collapse.

Our results are consistent with the findings of studies from Syria and Afghanistan [20,21], although some effect sizes were smaller than those found in non-conflict nursing samples. For example, in stable settings, stronger associations with depression (AOR > 4.0) have been documented [26] compared to our findings (AOR = 2.84). This discrepancy could be attributable to methodological differences (e.g., single-item versus multidimensional measures of burnout) or the possibility that in extreme conflict settings, the accumulation of multiple stressors dilutes the predictive power of any single factor. Similar patterns have been seen in other conflict-affected populations where the convergence of stressors produces a generalized distress response rather than disorder-specific patterns [12,27]. Several hypothesized associations were not supported. Despite the consistent associations between facility type and employment type and nurse wellbeing and retention in stable settings [23], it was surprising that there were no significant associations for these factors and most outcomes. In conflict-affected Yemen, this may represent a “homogenizing effect” of protracted crisis, whereby systemic conflict-related stressors such as insecurity, displacement, and salary disruption drown out institutional and contractual differences. If salary payments are irregular in both public and private sectors and safety issues are common, then protective or risk factors related to the type of facility or employment contract are of relatively little importance. This finding may indicate that in extreme humanitarian settings, interventions targeting structural conflict-related determinants may be more rewarding than those targeting facility-level or contractual variations. However, this interpretation is still speculative and needs to be investigated through qualitative research to understand the contextual mechanisms behind these non-findings. Supplementary Table S11 presents a complete summary of all non-significant findings.

Likewise, the borderline significance for age (*p* ≈ 0.051) across all outcomes was unexpected, given evidence that younger nurses may be more vulnerable to distress [28]. This may reflect a “leveling” effect of conflict, whereby universal exposure to trauma and insecurity diminishes the influence of age or experience. Alternatively, it may be a consequence of the narrow age range in our sample (22–58 years) and the relative homogeneity of exposure to conflict-related stressors.

The non-significant association between nurse–patient ratios of 1:15 versus 1:10 (AOR = 1.30, *p* = 0.226) suggests a pattern among workload categories where moderate increases in patient load may not be associated with detectable differences in distress, while more extreme ratios (≥1:20) are associated with stronger associations. This is a cross-sectional observational study, and the categories are observational rather than experimentally defined thresholds. Therefore, this finding should be described as a pattern across workload categories rather than evidence of a clinically meaningful threshold. These results are consistent with implications for staffing policy that suggest a focus on avoiding extreme ratios, but do not establish a causal threshold.

Subgroup analyses suggested generally consistent patterns across facility types, gender, and displacement status (Supplementary Table S3). Suggesting that these relationships are robust and generalizable across different segments of the nursing workforce.

### 6.2. Theoretical Implications

The remarkable similarity in the risk factor profiles for depression and anxiety is in line with evidence that these conditions often co-occur and share common etiological pathways under chronic stress [29]. The convergence of occupational and conflict-related stressors in conflict settings appears to generate a generalized distress response rather than disorder-specific patterns, in line with findings from other humanitarian contexts [12,27].

This study uses and builds on the Job Demands–Resources (JD-R) framework within the framework of a protracted armed conflict [11]. Our results are consistent with JD-R predictions: conflict-related job demands (violence, displacement) were consistently associated with psychological distress, while job resources (psychological support, adequate income) were associated with lower psychological distress. The observed dose–response relationships also support the JD-R premise that demands and resources are on a continuum.

Our findings provide context on how the JD-R model can be applied in conflict-affected health systems where conflict-related demands co-exist with broken organizational resources. The observed associations between violence, displacement, inadequate income, psychological support and mental health outcomes are generally consistent with JD-R propositions and need further evaluation using longitudinal designs [11].

These extensions have implications for adapting the JD-R framework to fragile health systems where traditional occupational resources are often lacking. Future research should test the applicability of the JD-R model in humanitarian settings using longitudinal designs that can demonstrate temporal precedence.

### 6.3. Psychological Support as a Moderator

The association between conflict exposures (displacement and workplace violence) and depressive symptoms differed according to the reported availability of workplace psychological support (β for interaction = −0.23, *p* < 0.001). Nurses who reported that psychological support was available showed weaker associations between conflict exposures and depressive symptoms than those who reported no available support.

This observation is compatible with stress-buffering and social support theories [30]; however, because psychological support was measured only in terms of availability (not utilization, frequency, intensity, or quality of support received), and because the cross-sectional design precludes causal inference, this finding should be described as an observed statistical interaction rather than as evidence that psychological support exerts a protective or statistical interaction. We cannot determine whether the observed association is attributable to access to support, actual use of support, or other unmeasured factors (e.g., organizational culture). Reverse causation and selection effects are also possible.

In the setting of Yemen, where specialized psychiatric services are few, even low-intensity or non-specialist interventions may be associated with lower distress. However, this study cannot establish that psychological support reduces distress, and longitudinal studies are needed to evaluate whether such support is associated with improved mental health outcomes over time.

### 6.4. Turnover Intention: A Threat to System Sustainability

The strong association of psychological distress and burnout with turnover intention (AOR = 3.42–4.15) is a serious threat to the sustainability of the health system. More than half of the nurses reported that they would leave, which is consistent with international evidence that psychological strain is associated with turnover [5,31]. High intention to turnover may further destabilize workforce in fragile health-systems. Nurses who leave are seldom replaced, especially in conflict-affected areas where systems of recruitment have been broken. The results suggest that retention strategies should take into account psychological wellbeing as well as contractual and financial factors, although the cross-sectional design cannot prove that reducing psychological distress will reduce turnover. These findings align with research from other low-resource settings such as Nigeria, where burnout has been identified as a significant driver of turnover intention among nurses [23].

### 6.5. Considerations in Methodology

The study represents nurses working in accessible conflict-affected governorates and hence may not be representative of inaccessible areas. Also, the cross-sectional design does not allow for causal inference.

### 6.6. Limitations

There are some limitations to be addressed. First, PTSD was not assessed, and a single-item measure of self-rated burnout was used. Both were pragmatic decisions, weighed against operational feasibility in a stressed, conflict-affected population. Lack of PTSD screening is an important limitation given the high levels of trauma exposure in our sample (displacement 54%, workplace violence 64%) and the fact that PTSD is one of the most common mental health disorders in conflict-affected populations [32,33]. The single-item measure of self-rated burnout is pragmatic but does not reflect the multidimensional nature of burnout (emotional exhaustion, depersonalization, and reduced personal accomplishment). This limitation restricts interpretation of the exploratory analyses involving burnout. Therefore, the supplementary exploratory indirect association findings are hypothesis-generating only, not evidence of causal mediation. Replication using the full Maslach Burnout Inventory (MBI) in longitudinal designs is warranted. Conceptual overlap between burnout and depressive symptoms, as reported in the literature, further complicates interpretation and calls for multidimensional assessment [34,35].

Other limitations include the cross-sectional design that precludes any causal inference; the self-report measures that may suffer from social desirability and recall bias; and the exclusion of the most contested northern governorates for safety reasons, meaning our findings are generalizable only to accessible areas.

The moderate correlation (r = 0.58) between the PHQ-9 and GAD-7 may indicate that depressive and anxiety symptoms are separate but related constructs, sharing 34% of the variance. Psychological support, workplace violence, and turnover intention were measured using either dichotomous or single-item measures, which restricts our capacity to capture the frequency, severity, type, or utilization dimensions that might have differential impacts. Future research should employ validated multi-item scales (e.g., TIS-6 for turnover) and more sophisticated measures of violence and support.

The current study focused on risk factors and did not measure protective mechanisms (resilience, coping, social support outside work) that may contribute to residual confounding. Future research should examine possible protective factors that may be associated with lower psychological distress among nurses exposed to conflict-related stressors.

Common Method Bias: Harman’s single-factor test was used as a limited descriptive diagnostic. The first unrotated factor explained 28.4% of the variance. This finding is reported only descriptively and does not rule out the possibility of common method variance [36], in particular because the main exposures and outcomes are self-reported on the same questionnaire. The study design and the limitations of the measurement themselves should be viewed as the main safeguards against common method bias. Future research should use multi-method designs and objective measures where possible.

Sampling Weights: Because equal allocation (~100 nurses per governorate) was used, selection probabilities differed across governorates, and unweighted estimates cannot be interpreted as population prevalence estimates for nurses across the accessible sampling frame. To address this, sampling-weighted prevalence estimates were calculated using inverse-probability weights based on governorate-level nursing workforce sizes obtained from the Ministry of Public Health and Population and Health Cluster Yemen (Supplementary Table S11, Supplementary File S4). The weighted estimates were nearly identical to the unweighted estimates (absolute differences < 1 percentage point), supporting the robustness of the findings to differential selection probabilities. For population-level interpretation, the weighted estimates should be preferred. However, because the most heavily conflict-affected northern governorates were inaccessible, even weighted estimates cannot be generalized to those areas.

Turnover Intention Model Specification: The turnover intention model in Table 4 did not control for mental health variables (depressive symptoms, anxiety symptoms, burnout), estimating overall associations. A model adjusted for these variables is shown in Supplementary Table S2c (Supplementary File S4). Controlling for mental health variables substantially changes the estimated associations between some exposures and turnover intention, as mental health variables could be on the same conceptual pathway as the outcome of interest. This is an important difference for interpretation.

Missing Data: Missing data were minimal (<3% for all key variables). Multiple imputation by chained equations (MICE) was used with 20 imputations, and results were compared to complete-case analyses. We obtained very similar results with differences of 0.01 to 0.02, confirming that the missing data had no substantial effect on our findings.

Model Performance: Internal validation was performed using 10-fold cross-validation, and AUC and Brier scores were obtained. As expected, the performance on the same data that was used to fit the models (apparent performance) was slightly higher. We report the cross-validated metrics as a more conservative estimate of the performance of the model.

The primary analyses were pre-specified (Supplementary File S1), but exploratory analyses of pathways, effect modification, and interaction included many tests without correction for multiple comparisons, increasing the risk of Type I error. We did not correct multiple comparisons to avoid masking any potentially important patterns for future investigations, and these findings should be considered hypothesis-generating. We are also aware of the risk of reporting bias with possible underreporting of non-significant findings. To aid balanced interpretation, we explicitly reported non-significant results (e.g., for facility type, employment type, and nurse–patient ratio of 1:15) in the main text. A detailed summary is shown in Supplementary Table S10 (Supplementary File S4). Effect size estimates (Cohen’s f^2^) are available in the Supplementary Materials.

Selection Bias: Selection bias could occur due to the replacement of non-participants (n = 72; 7.6% of the 944 nurses approached) if non-participants differ systematically from participants in unmeasured characteristics, particularly in mental health status. The overall response rate was 84.7% (800/944). Because mental health outcomes and other key variables were not available for non-participants, a formal comparison of observable characteristics between participants and non-participants could not be performed, and selection bias based on unmeasured factors cannot be excluded.

**Strengths**: The study possesses important strengths even considering the above limitations. The large, multicenter sample (n = 800) offers the most complete evidence to date on the mental health of Yemeni nurses in accessible areas. The use of validated Arabic instruments, multistage probability sampling, and mixed-effects models with facility-level random intercepts improves validity and generalizability. Strong sensitivity analyses (Supplementary File S4) provide evidence of robustness. The de-identified dataset, analyses code, and protocol are available openly through OSF (https://doi.org/10.17605/OSF.IO/M7QKG) for replication in other humanitarian settings.

### 6.7. Implications for Policy, Practice and Research

The findings have important implications for humanitarian actors, policy makers, nursing leaders and researchers in conflict-affected settings.

### 6.8. Humanitarian Policy & Practice

Dedicated funding for mental health and psychosocial support for healthcare workers must be treated by donors and Health Clusters as essential, not optional. Agencies should enhance protection mechanisms within humanitarian frameworks, including specific attention to the safety of health-care workers within ceasefire agreements and protection strategies. Workforce preservation needs to be recognized as a key part of health system strengthening, with indicators on healthcare worker wellbeing, stability and retention included in humanitarian response plans. Humanitarian actors should work with local health authorities to build confidential, non-punitive support systems, using community-based and peer-support approaches where specialist services are limited.

### 6.9. Nursing Leadership, Policy and Clinical Management

The dose–response relation between the frequency of night shifts and distress (26% increase per additional shift) may indicate that nurse managers should consider prioritizing shift scheduling (rotation limits, recovery periods and on-site rest facilities). The odds of depressive symptoms were nearly three times higher with 1:25 ratios than 1:10 ratios, highlighting the potential importance of evidence-based staffing policies. The lack of significant findings for 1:15 ratios are in line with a general pattern across workload categories where moderate increases in patient load may be associated with distress in ways that are not evident, whereas higher ratios (≥1:20) are more strongly associated. This pattern may inform discussions of staffing policy but does not define a clinically meaningful threshold.

The association between conflict exposures and depressive symptoms varied according to reported availability of workplace psychological support, suggesting that accessible mental health services need further evaluation in longitudinal and intervention studies within standard occupational health systems, such as confidential counseling, peer-support programs, and psychological first-aid training. However, because support was only measured as availability, and the cross-sectional design does not allow for causal inference, this support should be seen as a possible complement to structural reforms, not as a proven intervention.

The strong association between workplace violence and depressive symptoms, together with the finding that 87.5% of nurses exposed to violence did not report it, suggests that nurse managers should consider policies of zero tolerance, confidential reporting, and clear accountability. Organizational culture change requires leadership commitment to staff safety; however, this study cannot establish whether such interventions would reduce distress.

Strong associations with turnover intention (AOR = 2.88 for anxiety symptoms, 3.42 for depressive symptoms, and 4.15 for high burnout) imply that retention is contingent on mental health and working conditions. Retention strategies should consider psychological wellbeing and financial considerations as low income was independently associated with depressive symptoms (AOR = 2.53, 95% CI: 1.80–3.56) and turnover intention (AOR = 2.12, 95% CI: 1.54–2.92).

### 6.10. Nurse Education and Practice

Nursing education needs to improve training in self-care, resilience and trauma-informed care. Nurse managers and senior staff should also be trained to recognize early signs of burnout and to provide appropriate support. Healthcare organizations should encourage reporting and documentation of workplace violence and unsafe staffing conditions to support evidence-based policy and workforce improvements.

### 6.11. Future Research

Longitudinal and intervention studies are needed to evaluate effective strategies to support nurses in conflict situations. The high levels of displacement (54%) and workplace violence (64%) are a major limitation, as post-traumatic stress disorder (PTSD) has not been evaluated and should be explored in future studies using validated instruments to evaluate PTSD. Future research should also employ multidimensional burnout instruments (e.g., full Maslach Burnout Inventory [MBI]) and validated turnover intention scales (e.g., Turnover Intention Scale-6 [TIS-6]) [37] to obtain a more holistic picture of the workforce retention and mental health outcomes. Longitudinal designs are needed to demonstrate the time precedence and causal direction. The study protocol (Supplementary File S1) and validated Arabic instruments (Supplementary File S2) are available for replication in similar humanitarian settings.

## 7. Conclusions

Levels of depressive symptoms, anxiety symptoms, self-rated burnout and turnover intention among nurses working in the eight accessible conflict-affected governorates of Yemen were high in this study, suggesting major challenges to workforce stability and sustainability of health services. The findings are limited to the governorates involved in the study as some conflict-affected areas were not accessible during data collection. Conflict exposures were associated with depressive symptoms, and this association was different in relation to reported availability of workplace psychological support but needs to be confirmed in longitudinal studies. The findings suggest potential areas for intervention, such as the integration of mental health services, prevention of workplace violence, protection of the workforce, and the implementation of safe staffing strategies (e.g., avoiding extreme nurse-to-patient ratios [≥1:20] and limiting consecutive night shifts). Such considerations could be taken into account by nursing leadership and health system recovery initiatives in conflict settings. However, this study cannot prove the effectiveness of these interventions. Longitudinal and intervention studies are required. Future studies should include PTSD assessment and longitudinal designs to improve understanding of the long-term effects of conflict on nurses’ wellbeing and retention.

## Figures and Tables

**Table 1:** Demographic, Occupational, and Conflict-Related Characteristics (N = 800).

Characteristic	n (%) or Mean (SD)
**Demographic**	
**Age (years)** Mean (SD)	38.7 (10.2)
**Gender:** Male	364 (45.5)
Female	436 (54.5)
**Marital status**: single	348 (43.5)
Married	452 (56.5)
**Education:** Diploma	342 (42.8)
Bachelor	336 (42.0)
Master	122 (15.3)
**Experience (years):** Mean (SD)	15.3 (9.8)
**Employment type:** Permanent	388 (48.5)
Contract	412 (51.5)
**Facility type:** Public	416 (52.0)
Private	384 (48.0)
**Working hours/shift:** 8 h	264 (33.0)
10 h	232 (29.0)
12 h	304 (38.0)
**Night shifts/month**	7.8 (2.9)
**Nurse–patient ratio:** ≤1:15	448 (56.0)
≥1:20	352 (44.0)
**Conflict-Related Exposures**	
Displacement status: Displaced	432 (54.0)
Non-displaced	368 (46.0)
Monthly income: adequate	348 (43.5)
Inadequate	452 (56.5)
Workplace violence: exposed	512 (64.0)
Not exposed	288 (36.0)
Psychological support: available	480 (60.0)
Not available	320 (40.0)

Note: Nurse-to-patient ratio is presented in collapsed form (≤1:15 and ≥1:20) for descriptive purposes only. Regression analyses (Table 4, Supplementary Table S2) used the full four-category classification (1:10, 1:15, 1:20, 1:25).

**Table 2:** Characteristics of Participating Governorates and Health Facilities.

Region	Governorates Included	Facilities (n)	Participants (n)	Response Rate (%)	Security Level
South	Aden, Lahj, Abyan	9	289	83.3	Moderate
Central	Shabwah, Taiz	6	213	89.1	Moderate to High
East	Hadramawt, Al-Mahra, Socotra	9	298	83.2	Moderate
**Total**	**8 governorates**	**24**	**800**	**84.7**	**—**

**Note**: Response rates are calculated as (Nurses Participated)/(Total Approached) × 100. The total number of nurses approached was 944 (872 in the initial systematic random sampling sequence plus 72 replacements for non-participants), of whom 800 participated (overall response rate: 84.7%). Region-level aggregation is presented to protect the confidentiality of individual governorates with small nursing workforces (e.g., Socotra, Al-Mahra). Detailed facility-level characteristics and sampling details are provided in Supplementary File S10 (Supplementary Table S12a–d).

**Table 3:** Prevalence of Depressive Symptoms, Anxiety Symptoms, Burnout, and Turnover Intention (N = 800).

Outcome/Category	n (%) [95% CI]	Mean (SD)
**Depressive Symptoms (PHQ-9)**		**12.4 (7.2)**
Minimal (0–4)	136 (17.0) [14.4–19.8]	—
Mild (5–9)	248 (31.0) [27.8–34.3]	—
Moderate+ (≥10)	416 (52.0) [48.5–55.5]	—
Moderate (10–14)	192 (24.0) [21.1–27.1]	—
Moderately severe (15–19)	144 (18.0) [15.4–20.8]	—
Severe (20–27)	80 (10.0) [8.0–12.2]	—
**Anxiety Symptoms (GAD-7)**		**10.8 (6.5)**
Minimal (0–4)	184 (23.0) [20.1–26.1]	—
Mild (5–9)	240 (30.0) [26.8–33.3]	—
Moderate+ (≥10)	376 (47.0) [43.5–50.5]	—
Moderate (10–14)	232 (29.0) [25.9–32.2]	—
Severe (15–21)	144 (18.0) [15.4–20.8]	—
**Burnout Level**		**1.96 (0.84)**
Low	304 (38.0) [34.6–41.5]	—
Moderate	224 (28.0) [24.9–31.2]	—
High	272 (34.0) [30.7–37.4]	—
**Intention to Leave**		**—**
Yes	438 (54.8) [51.2–58.2]	—
No	362 (45.2) [41.7–48.8]	—

**Notes: 1.** PHQ-9 and GAD-7 are screening instruments, not diagnostic tools. A score ≥ 10 indicates clinically significant depressive symptoms or moderate-to-severe anxiety symptoms, respectively, but does not establish a clinical diagnosis. **2.** “Moderate+” for depressive symptoms (PHQ-9 ≥ 10) is the sum of “Moderate” (10–14), “Moderately severe” (15–19), and “Severe” (20–27): 192 + 144 + 80 = 416. Similarly, “Moderate+” for anxiety symptoms (GAD-7 ≥ 10) is the sum of “Moderate” (10–14) and “Severe” (15–21): 232 + 144 = 376. **3.** Burnout was assessed with a single-item measure with five original response categories (None, Low, Moderate, High, and Severe). For analyses, None and Low were combined into “Low” (coded 1); Moderate remained “Moderate” (coded 2); High and Severe were combined into “High” (coded 3). The resulting variable is ordinal (1 = Low, 2 = Moderate, 3 = High). “High burnout” prevalence (34.0%) represents the proportion of participants who responded “High” or “Severe” (n = 272). This coding was prespecified in the protocol (Supplementary File S1) and used consistently across all analyses (ordinal logistic regression, turnover intention model, SEM). **4.** Mean (SD) for Depressive Symptoms and Anxiety Symptoms represent the mean total score across all 800 participants. Mean (SD) for Burnout Level was calculated by treating the ordinal variable as continuous (1 = low, 2 = moderate, 3 = high). **5.** Intention to Leave is a binary variable (Yes/No); mean (SD) is not reported. 6. “High self-rated burnout” (34.0%, n = 272) represents the proportion of participants who responded “High” or “Severe” on the original five-point single-item measure. This terminology is used throughout to reflect the self-report nature of the measure.

## Data Availability

The study protocol (Supplementary File S1), study instruments in Arabic and English (Supplementary File S2), reporting checklists (Supplementary File S3), sensitivity analyses including sampling table and non-significant findings summary (Supplementary File S4), supplementary analyses (Supplementary File S5), data dictionary (Supplementary File S6), de-identified dataset (Supplementary File S7), analyses code in R (Supplementary File S8), ethical approval certificate (Supplementary File S9), and facility-level characteristics (Supplementary File S10) are publicly available in the Open Science Framework repository: https://doi.org/10.17605/OSF.IO/M7QKG. The dataset and code are provided to facilitate replication, secondary analyses, and adaptation in other humanitarian settings. Users are requested to cite this original study when using these materials.

## Supplementary Materials

The following supporting information can be downloaded at: https://www.mdpi.com/article/10.3390/nursrep16100353/s1, Supplementary File S1: Study Protocol; Supplementary File S2: Study Instruments (Questionnaires and Consent Forms); Supplementary File S3: Reporting Checklists; Supplementary File S4: Sensitivity Analyses; Supplementary File S5: Supplementary Analyses; Supplementary File S6: Data Dictionary; Supplementary File S7: De-identified Dataset; Supplementary File S8: Statistical Analyses Code (R); Supplementary File S9: Ethical Approval Certificate; Supplementary File S10: Facility-Level Characteristics. Refs [38,39] are cited in Supplementary Materials file.

## Public Involvement Statement

No public involvement in any aspect of this research. This study was designed, conducted, and analyzed by the research team without direct involvement of patients, nurses, or members of the public in the design, conduct, reporting, or dissemination plans. However, the study findings will be disseminated to participating health facilities, local health authorities, and humanitarian partners to inform nursing leadership and workforce protection strategies.

## Guidelines and Standards Statement

This manuscript was prepared in accordance with the STROBE and SAGER reporting guidelines. Completed checklists are available in Supplementary File S3.

## Use of Artificial Intelligence

During the preparation of this manuscript, the authors used ChatGPT (OpenAI, GPT-4) for language polishing, structural suggestions, and to enhance the clarity of certain analytical descriptions. These tools were employed after all data collection, statistical analyses, and initial interpretation were completed by the human authors. All content generated with AI assistance was rigorously reviewed, fact-checked against the original data and statistical outputs and substantially edited by the authors. The authors take full responsibility for the final content, interpretations, and conclusions presented in this published article, and confirm that no AI tools were used for data analyses, interpretation of results, or drawing scientific conclusions.
