# Supplementary material for: Mental Health, Burnout, and Turnover Intention Among Nurses in Conflict-Affected Yemen: A Multicenter Study Across Eight Accessible Governorates
Source: Nurs Rep. 2026 Oct 7;16(10):353. doi: 10.3390/nursrep16100353 (PMC13708594; doi:10.3390/nursrep16100353)
Supplement: Supplementary file 1 [file nursrep-16-00353-s001.zip › File S1 Study Protocol.pdf]

File S1: STUDY PROTOCOL

Mental Health, Burnout and Turnover Intention among Nurses in Conflict-Affected Yemen: A Multicentre Study Across Eight Accessible Governorates

Principal Investigator
Dr. Naif Taleb Ali, PhD

Department of Health Sciences, Faculty of Medicine and Health Sciences
University of Science and Technology, Aden, Yemen

January 2025

IRB Reference Number: YEM-IRB-2025-094

Table of Contents

Section	Content
1	Study Title
2	Principal Investigator
3	Study Objectives
4	Study Design
5	Sample Size Calculation
6	Sampling Strategy
7	Study Instruments
8	Data Collection Procedures
9	Safety Protocol for PHQ-9 Item 9
10	Statistical Analysis Plan
11	Primary and Exploratory Analyses
12	Ethical Considerations
13	Data Management and Availability
14	Timeline
15	References

1. Study Title

Full Title: **Mental Health, Burnout and Turnover Intention among Nurses in Conflict-Affected Yemen: A Multicentre Study Across Eight Accessible Governorates**

Short Title**: Psychological Distress Among Yemeni Nurses Study**

2. Principal Investigator

Dr. Naif Taleb Ali, PhD

Affiliation: Department of Health Sciences, Faculty of Medicine and Health Sciences, University of Science and Technology, Aden, Yemen

Email: n.taleb@ust.edu

Phone: +967 772 571 390

ORCID: 0000-0002-2266-1569

Co-Investigators:

Khalil A. Saleh - Department of Medical Surgical, College of Nursing, University of Ha'il, Ha'il, Saudi Arabia

Abdulhafith Yahya Alharbi - Department of Psychiatric and Mental Health Nursing, College of Nursing, University of Ha'il, Ha'il, Saudi Arabia

3. Study Objectives

3.1 Primary Objectives

Estimate the prevalence of depressive symptoms, anxiety symptoms, burnout, and turnover intention among nurses in conflict-affected Yemen.

Identify occupational and conflict-related factors associated with these outcomes.

Explore, using exploratory path analysis, whether the data are statistically consistent with burnout as a potential statistical indirect association compatible with the proposed model and psychological support as a possible effect modifier.

3.2 Primary Hypotheses

Based on the Job Demands-Resources (JD-R) framework [11], we hypothesise that:

H1: Conflict-related job demands (displacement, workplace violence) will be positively associated with depressive symptoms, anxiety symptoms, burnout, and turnover intention.

H2: Job resources (psychological support, adequate income) will be negatively associated with depressive symptoms, anxiety symptoms, burnout, and turnover intention.

H3: The association between conflict exposures and psychological distress will differ according to the reported availability of workplace psychological support.

3.3 Exploratory Objectives

To examine whether the data are statistically consistent with burnout as a statistical indirect association compatible with the proposed model between conflict exposures and psychological distress.

To examine whether the association between conflict exposures and psychological distress differed according to the reported availability of workplace psychological support.

To examine synergistic interactions between multiple adversities (e.g., displacement + workplace violence; inadequate income + high nurse-patient ratio).

4. Study Design

4.1 Design

Type: Multicentre cross-sectional survey

Setting: Eight accessible conflict-affected governorates in Yemen (Aden, Lahj, Abyan, Shabwah, Hadramawt, Al-Mahra, Socotra, Taiz)

Duration: 12 months (January – December 2025)

Sample: 800 registered nurses

Data Collection Period: January 20, 2025 – December 31, 2025

4.2 Rationale for Cross-Sectional Design

A cross-sectional design was selected for the following reasons:

Feasibility: Longitudinal designs are challenging in active conflict zones due to security constraints, population displacement, and disruption of health services.

Resource constraints: Longitudinal studies require substantially more resources, which are limited in humanitarian settings.

Policy relevance: Cross-sectional estimates of prevalence and associated factors are directly relevant for immediate policy and programmatic responses.

Limitation acknowledged: The cross-sectional design precludes causal inference. Findings should be interpreted as associations, not causal relationships.

5. Sample Size Calculation

5.1 Formula

Sample size was calculated using Cochran's formula for prevalence estimation [13]:

$$n_0 = \frac{Z^2 \times p \times (1-p)}{e^2}$$

Where:

$Z = 1.96$ (95% confidence level)

$p = 0.50$ (anticipated prevalence - conservative estimate)

$e = 0.05$ (margin of error)

Calculation:

$$n_0 = \frac{1.96^2 \times 0.50 \times 0.50}{0.05^2} = 384.16 \approx 385$$

5.2 Design Effect Adjustment

For cluster sampling, a design effect of 1.5 was applied. The design effect of 1.5 was selected based on:

Assumed intracluster correlation coefficient (ICC): 0.05

Average cluster size: 11 nurses per facility

Formula: Design Effect $= 1 + (m - 1) \times ICC = 1 + (11 - 1) \times 0.05 = 1.5$

Adjusted Sample Size:

$$n_1 = 385 \times 1.5 = 577.5 \approx 578$$

5.3 Non-Response Adjustment

Anticipated non-response rate: 10%

Final Sample Size:

$n_2 = \frac{578}{1 - 0.10} = 642.2 \approx 643$

5.4 Final Sample Size

The sample size was increased from the calculated 643 to 800 participants to:

Compensate for anticipated non-response (approximately 10%)

Permit subgroup analyses (e.g., by governorate, facility type)

5.5 Power Analysis

80% power to detect odds ratios of ≥1.5 for binary outcomes

80% power to detect effect sizes of ≥0.2 SD for continuous outcomes

6. Sampling Strategy

6.1 Overview

A multistage probability sampling strategy was used to ensure broad geographic and institutional representation.

6.2 Stage 1: Selection of Eligible Governorates

Eligible Governorates:

Yemen has 22 governorates, of which 15 were classified as conflict-affected. Eight accessible governorates were included in the sampling frame:

Aden (South)

Lahj (South)

Abyan (South)

Shabwah (Central)

Hadramawt (East)

Al-Mahra (East)

Socotra (East)

Taiz (Central)

Excluded Governorates (7):

For safety reasons, governorates under active siege or with total health facility closure were excluded:

Sa'ada

Hajjah

Amran

Sana'a

Ibb

Dhamar

Al-Bayda

Rationale: The classification as 'conflict-affected' is consistent with the WHO definition of disruption to health systems due to armed conflict.

Limitation: Findings are generalisable only to accessible conflict-affected areas of Yemen.

6.3 Stage 2: Stratification of Facilities

Sampling Frame Development:

The sampling frame was developed using:

Latest lists of functioning health facilities from the Ministry of Public Health and Population

Records of local health authorities

Mapping by the humanitarian sector (Health Cluster Yemen)

Accessibility Assessment:

Facilities were deemed accessible based on security assessments carried out with:

Local authorities

Humanitarian partners

Facilities that were under siege, fully closed, or posed imminent safety risks were excluded.

Stratification:

Facilities were stratified according to:

Ownership: Public / Private

Level of care: Primary / Secondary / Tertiary

6.4 Stage 3: Facility Selection

Selection:

From the stratified sampling frame, 24 facilities were selected across the eight governorates.

3 facilities per governorate (total: $8 \times 3 = 24$)

Approximately 70% public and 30% private to reflect sector distribution

Rationale: This approach ensured representation of both public and private sectors while remaining operationally feasible under security constraints.

6.5 Stage 4: Selection of Nurses within Facilities

Systematic Random Sampling:

Within each selected facility, systematic random sampling was used to select eligible nurses:

Every second eligible nurse was invited (sampling fraction ≈ 0.50)

Equal Allocation:

Approximately 100 nurses per governorate were allocated to ensure adequate statistical power for governorate-level comparisons.

Rationale: This equal allocation was a pragmatic decision to ensure balanced representation across geographic regions, given that governorate-level nursing workforce sizes vary substantially.

Limitation: Because equal allocation was used, selection probabilities differed across governorates. The primary analyses were unweighted. Sampling-weighted sensitivity analyses were conducted to assess robustness.

6.6 Inclusion Criteria

Participants were eligible if they met ALL of the following:

Licensed registered nurse

Currently working in Yemen

Worked in a clinical setting for at least 3 consecutive months prior to data collection

Provided informed consent

6.7 Exclusion Criteria

Participants were excluded if they met ANY of the following:

Individuals in administrative roles only and without patient contact

Nurses on extended leave

Refused to participate

7. Study Instruments

7.1 Overview

The study questionnaire included the following sections:

Section	Content	Items
A	Sociodemographic Information	5
B	Occupational Characteristics	6
C	Conflict and Workplace Exposures	3
D	Support and Resources	1
E	PHQ-9 (Depressive Symptoms)	9
F	GAD-7 (Anxiety Symptoms)	7
G	Burnout Single-Item Measure	1
H	Turnover Intention	1

Full instruments are available in File S2.

7.2 Primary Outcome Measures

Depressive Symptoms: PHQ-9

Instrument: Patient Health Questionnaire-9 (PHQ-9), validated Arabic version

Items: 9

Scoring: 0 (not at all) to 3 (nearly every day)

Total Score Range: 0–27

Severity Categories:

Minimal: 0–4

Mild: 5–9

Moderate: 10–14

Moderately severe: 15–19

Severe: 20–27

Cut-off: ≥10 denotes clinically significant depressive symptoms

Scoring details: File S6 (Data Dictionary)

Anxiety Symptoms: GAD-7

Instrument: Generalised Anxiety Disorder-7 (GAD-7), validated Arabic version

Items: 7

Scoring: 0 (not at all) to 3 (nearly every day)

Total Score Range: 0–21

Severity Categories:

Minimal: 0–4

Mild: 5–9

Moderate: 10–14

Severe: 15–21

Cut-off: ≥10 denotes moderate-to-severe anxiety symptoms

Scoring details: File S6 (Data Dictionary)

7.3 Safety Protocol for PHQ-9 Item 9 (Self-Harm)

Rationale: The PHQ-9 includes Item 9, which asks about "Thoughts of self-harm." A specific safety protocol was implemented to address any endorsement of this item.

Protocol:

Trigger: Any non-zero response to Item 9 (i.e., responding "Several days," "More than half the days," or "Nearly every day") triggered an immediate individual risk assessment.

Immediate Risk Assessment:

Conducted by a trained nurse researcher

Includes structured evaluation of:

Suicidal ideation

Intent

Plan

Access to means

Referral:

Participants identified as at imminent risk were immediately referred to:

On-site psychiatrist or mental health professional (where available), OR

Nearest emergency department or mental health facility

A written referral pathway was provided to all participants who screened positive on Item 9.

Escalation Protocol: In conflict-affected settings where immediate referral was not feasible, the study team followed a pre-established escalation protocol coordinated with local health authorities and humanitarian partners.

IRB Approval: All safety procedures were reviewed and approved by the Institutional Review Board (IRB) prior to data collection (IRB Reference Number: YEM-IRB-2025-094, Date of Approval: January 15, 2025).

Adverse Events: No adverse events related to the safety protocol were reported during the study period.

7.4 Secondary Outcome Measures

7.4.1 Burnout Level: Single-Item Measure

Question: "How would you rate your level of burnout?"

Original Response Options: None (0), Low (1), Moderate (2), High (3), Severe (4)

Rationale: Pragmatic measure selected to minimise respondent burden in a highly stressed population

Burnout Coding for Analysis:

For analysis, the original five response categories were collapsed into three categories:

Original Response	Collapsed Coding
None (0)	Low (1)
Low (1)	Low (1)
Moderate (2)	Moderate (2)
High (3)	High (3)
Severe (4)	High (3)

"High burnout" Definition: Burnout Level = 3 (original response High or Severe)

Prespecified: Yes

7.4.2 Burnout Validation

To ensure validity in our setting, a pilot test of 40 nurses recruited from two facilities in Aden was conducted between January 16–19, 2025 (after IRB approval on January 15, 2025; prior to the main data collection). In this pilot, participants simultaneously completed the MBI-Human Services Survey (MBI-HSS) emotional exhaustion subscale (Arabic version).

The correlation between the single-item measure and the MBI emotional exhaustion subscale was $r = 0.82$ (95% CI: 0.71–0.89, $p < 0.001$), estimated using Pearson's correlation coefficient after

confirming normal distribution of both variables (Shapiro-Wilk $p > 0.05$). The five response categories were used during this validation. This was an original validation conducted specifically for the present study.

Limitations: The single-item measure cannot capture the multidimensional nature of burnout (emotional exhaustion, depersonalisation, reduced personal accomplishment). The findings concerning burnout, particularly the supplementary analyses, need to be interpreted cautiously and validated by means of validated multidimensional instruments in future research.

7.4.3 Turnover Intention: Single-Item Measure

Question: "Do you intend to leave your current nursing position within the next 12 months?"

Response Options: Yes / No

Rationale: Single-item measures of turnover are common in nursing workforce research and possess predictive validity for actual turnover [14].

Limitation: Multi-item scales (e.g., TIS-6 [36] provide a more complex measurement of turnover cognitions

7.5 Independent Variables

Sociodemographic:

Age (continuous)

Sex (Male / Female)

Marital status (Single / Married / Divorced / Widowed)

Education (Diploma / Bachelor / Master)

Occupational:

Years of experience (continuous)

Type of employment (Permanent / Contract)

Type of facility (Public / Private)

Hours worked per shift

Night shifts per month

Nurse-to-patient ratio

Nurse-to-Patient Ratio:

Categories for regression analyses: 1:10 (reference), 1:15, 1:20, 1:25

Categories for descriptive presentation (Table 1): ≤1:15 and ≥1:20

Rationale: Four categories used in regression models to examine dose-response relationships; two categories used for descriptive purposes

7.6 Conflict and Exposure at Work

Workplace Violence:

Measurement: Dichotomous (exposed / not exposed)

Definition: Any physical, verbal, or psychological aggression from patients, relatives, or colleagues in the previous 6 months

Multiple Types: Participants could report multiple types (physical, verbal, psychological) and multiple sources (patients, relatives, colleagues)

Main Analysis Coding: Any exposure to any type from any source was coded as "exposed"

Descriptive Reporting: Types and sources are presented separately in Table S6

Displacement Status:

Measurement: Single question: "Are you currently displaced due to the conflict?" (Yes / No)

Coding: Participants who responded "Yes" were coded as displaced

Monthly Income Adequacy:

Measurement: Single question: "Is your monthly income adequate to cover your living expenses?" (Adequate / Inadequate)

Rationale: Self-rated measure based on participants' perception of their financial situation relative to local living costs

Psychological Support:

Measurement: Single question: "Are mental health support services (e.g., counselling, peer support, or referral pathways) available at your workplace?" (Yes / No)

Limitation: This dichotomous assessment does not capture type, frequency, intensity, quality, or actual utilisation of services

8. Data Collection Procedures

8.1 Data Collection Team

Trained local nurse researchers

Approved security procedures

5-day standardised training on study instruments and safety protocols

8.2 Data Collection Process

Facility Selection: 24 facilities across 8 governorates

Nurse Invitation: Systematic random sampling (every second eligible nurse)

Informed Consent: Written informed consent obtained from all participants

Questionnaire Administration: 20–30 minutes

Safety Protocol: Applied for any PHQ-9 Item 9 endorsement

Data Quality Checks: Daily validation checks (10%)

8.3 Data Quality Assurance

Real-time range/consistency checks

Weekly data review meetings

Automated data cleaning scripts

Missing data assessment (<3%)

Outlier detection and handling

Multicollinearity checks (VIF < 5)

Model assumption verification

9. Safety Protocol for PHQ-9 Item 9

Rationale: The PHQ-9 includes Item 9, which asks about "Thoughts of self-harm." A specific safety protocol was implemented to address any endorsement of this item.

Protocol:

Trigger: Any non-zero response to Item 9 (i.e., responding "Several days," "More than half the days," or "Nearly every day") triggered an immediate individual risk assessment.

Immediate Risk Assessment:

Conducted by a trained nurse researcher

Includes structured evaluation of:

Suicidal ideation

Intent

Plan

Access to means

Referral:

Participants identified as at imminent risk were immediately referred to:

On-site psychiatrist or mental health professional (where available), OR

Nearest emergency department or mental health facility

A written referral pathway was provided to all participants who screened positive on Item 9.

Escalation Protocol: In conflict-affected settings where immediate referral was not feasible, the study team followed a pre-established escalation protocol coordinated with local health authorities and humanitarian partners.

IRB Approval: All safety procedures were reviewed and approved by the Institutional Review Board (IRB) prior to data collection (IRB Reference Number: YEM-IRB-2025-094, Date of Approval: January 15, 2025).

Adverse Events: No adverse events related to the safety protocol were reported during the study period.

10. Statistical Analysis Plan

10.1 Software

Statistical analyses will be performed using:

R version 4.3.1 (R Foundation for Statistical Computing, Vienna, Austria)

Packages: lme4, ordinal, mice, sandwich, lmtest, lavaan, performance, sjPlot, gtsummary

10.2 Descriptive Statistics

Continuous variables: Mean (SD) or Median (IQR)

Categorical variables: n (%)

10.3 Bivariate Analyses

Chi-square test

Fisher's exact test

t-tests

Mann-Whitney U tests

10.4 Primary Analyses

Mixed-effects logistic regression with facility-level random intercepts for binary outcomes (depressive symptoms, anxiety symptoms, turnover intention)

Mixed-effects ordinal logistic regression with facility-level random intercepts for burnout

Mixed-effects linear regression with facility-level random intercepts for continuous PHQ-9 and GAD-7 scores

Model Building: Based on pre-specified JD-R framework

10.5 Turnover Intention Model Specification

For the turnover intention outcome, two models will be presented:

Model 1 (Table 4, main text): Associations between occupational/conflict exposures and turnover intention without adjustment for mental health variables (depression, anxiety, burnout), estimating overall associations.

Model 2 (Table S2c, File S4): Associations conditional on psychological distress, adjusting for depression, anxiety, and burnout.

Rationale: Mental health variables may lie on the same conceptual pathway as turnover intention, and their inclusion could substantially alter the estimated associations.

10.6 Model Diagnostics

Multicollinearity: Variance inflation factors (VIF), with values >5 considered problematic

Discrimination: Area under the receiver operating characteristic curve (AUC)

Calibration: Calibration plots and Brier scores

Overall fit: Akaike Information Criterion (AIC) and Hosmer-Lemeshow tests

Influence diagnostics: Cook's distance and DFBETAS [16].

10.7 Exploratory Analyses

Structural Equation Modelling (SEM):

Estimated using robust maximum likelihood (MLR) for continuous outcomes

Weighted least squares mean and variance adjusted (WLSMV) for binary outcomes

Fit indices: CFI, TLI, RMSEA, SRMR

Effect Modification:

Interaction terms between psychological support and each conflict exposure

Simple slopes analysis

Synergistic Interactions:

Interaction terms between multiple adversities

10.8 Sensitivity Analyses

Multiple Imputation: MICE with m = 20 imputations

Alternative Cut-offs: PHQ-9 ≥5 and ≥15; GAD-7 ≥5 and ≥8

Subgroup Analyses: By facility type, gender, displacement status

Outlier Sensitivity: Excluding PHQ-9 ≥20 and GAD-7 ≥15

Sampling-Weighted Models: To assess robustness to differential selection probabilities

10.9 Risk Ratios

To complement odds ratios, risk ratios will be estimated using Poisson regression with robust variance, as depressive and anxiety symptoms are common outcomes [19,17,18].

11. Primary and Exploratory Analyses

11.1 Primary Analyses (Pre-specified)

Estimation of prevalence of depressive symptoms, anxiety symptoms, burnout, and turnover intention

Identification of occupational and conflict-related factors associated with these outcomes

Mixed-effects models as described in Section 10.4

11.2 Exploratory Analyses (Pre-specified as Exploratory)

Structural equation modelling (SEM) for exploratory indirect associations

Effect modification analysis for psychological support

Synergistic interactions

Subgroup analyses

Note: Exploratory analyses are hypothesis-generating and should not be interpreted as evidence of causal pathways.

12. Ethical Considerations

12.1 Ethical Approval

IRB: Radfan College University Ethical Committee

IRB Reference Number: YEM-IRB-2025-094

Date of Approval: January 15, 2025

Expiry Date: January 14, 2026

12.2 Informed Consent

Written informed consent obtained from all participants

Consent forms in Arabic and English (File S2)

Participants informed of:

Study purpose

Procedures

Risks and benefits

Confidentiality

Voluntary participation

Right to withdraw at any time without penalty

12.3 Confidentiality

Responses are completely anonymous

No personally identifiable information collected

Data stored on encrypted servers

Published only in aggregate form

12.4 Safety Protocol

Detailed in Section 7.3 and Section 9

Approved by IRB prior to data collection

12.5 Ethical Guidance

Declaration of Helsinki

Ethical guidance for research in conflict-affected settings

13. Data Management and Availability

13.1 Data Management

De-identified dataset stored on encrypted servers

Analysis code documented and version-controlled

Data dictionary available (File S6)

13.2 Data Availability

The following materials are publicly available in the Open Science Framework repository (https://doi.org/10.17605/OSF.IO/M7QKG):

File	Content
File S1	Study Protocol
File S2	Study Instruments (Questionnaires and Consent Forms)
File S3	Reporting Checklists
File S4	Sensitivity Analyses
File S5	Supplementary Analyses
File S6	Data Dictionary
File S7	De-identified Dataset
File S8	Statistical Analysis Code (R)
File S9	Ethical Approval Certificate
File S10	Facility-Level Characteristics

13.3 Citation Request

Users are requested to cite this original study when using these materials.

14. Timeline

Phase	Activity	Timeline
1	Protocol	development September – November 2024
2	IRB	submission November – December 2024
3	IRB	approval January 15, 2025
4	Pilot	testing (n = 40) January 16–19, 2025
5	Main	data collection January 20 – December 31, 2025
6	Data	cleaning and analysis January – March 2026
7	Manuscript	preparation March – June 2026
8	Submission	June 2026

Note: All human-subjects activities (pilot testing and main data collection) were initiated after IRB approval on January 15, 2025. No participant was enrolled prior to this date.

15. References

11. Bakker, A. B., & Demerouti, E. (2017). Job demands-resources theory: Taking stock and looking forward. Journal of Occupational Health Psychology, 22(3), 273–285. https://doi.org/10.1037/ocp0000056

37. Bothma, C. F. C., & Roodt, G. (2013). The validation of the turnover intention scale. SA Journal of Human Resource Management, 11(1), Article 507. https://doi.org/10.4102/sajhrm.v11i1.507

13. Cook, W.G. (1977) Sampling Techniques. 3rd Edition, John Wiley & Sons.

16. Cook, R. D., & Weisberg, S. (1982). Residuals and influence in regression. Chapman & Hall.

17. Norton, E. C., Dowd, B. E., & Maciejewski, M. L. (2018). Odds Ratios-Current Best Practice and Use. JAMA, 320(1), 84–85. https://doi.org/10.1001/jama.2018.6971

14. Richer, S. F., Blanchard, C., & Vallerand, R. J. (2002). A motivational model of work turnover. Journal of Applied Social Psychology, 32(10), 2089–2113. https://doi.org/10.1111/j.1559-1816.2002.tb02065.x

19. Zhang, J., & Yu, K. F. (1998). What's the relative risk? A method of correcting the odds ratio in cohort studies of common outcomes. JAMA, 280(19), 1690–1691. https://doi.org/10.1001/jama.280.19.1690

18. Zou, G. (2004). A modified Poisson regression approach to prospective studies with binary data. American Journal of Epidemiology, 159(7), 702–706. https://doi.org/10.1093/aje/kwh090
