# Supplementary material for: Mental Health, Burnout, and Turnover Intention Among Nurses in Conflict-Affected Yemen: A Multicenter Study Across Eight Accessible Governorates
Source: Nurs Rep. 2026 Oct 7;16(10):353. doi: 10.3390/nursrep16100353 (PMC13708594; doi:10.3390/nursrep16100353)
Supplement: Supplementary file 1 [file nursrep-16-00353-s001.zip › File S2 Study Instruments Questionnaires.pdf]

FILE S2: STUDY INSTRUMENTS (QUESTIONNAIRES)

Study Title: Mental Health, Burnout and Turnover Intention among Nurses in Conflict-Affected Yemen: A Multicentre Study Across Eight Accessible Governorates

Prepared as a bilingual study instrument and supporting documentation

FILE S2: STUDY INSTRUMENTS (QUESTIONNAIRES)

Study Title: **Mental Health, Burnout and Turnover Intention among Nurses in Conflict-Affected Yemen: A Multicentre Study Across Eight Accessible Governorates**

TABLE OF CONTENTS

1.	English Version —Complete Questionnaire
2.	Arabic Version -Complete Questionnaire
3.	Informed Consent Form -English
4.	Informed Consent Form -Arabic
5.	Burnout Measure -Detailed Description and Validation
6.	PHQ-9 Item 9 -Safety Protocol (Reminder)
7.	References

PART 1: ENGLISH VERSION -COMPLETE QUESTIONNAIRE

SECTION A: SOCIODEMOGRAPHIC INFORMATION

A1. Age: ______ years

A2. Gender:

☐ Male

☐ Female

A3. Marital Status:

☐ Single

☐ Married

☐ Divorced

☐ Widowed

A4. Education Level:

☐ Diploma

☐ Bachelor's

☐ Master's

☐ Doctorate

A5. Current Governorate: _______________

SECTION B: OCCUPATIONAL CHARACTERISTICS

B1. Years of nursing experience: ______ years

B2. Employment Type:

☐ Permanent

☐ Contract

☐ Volunteer

B3. Facility Type:

☐ Public

☐ Private

☐ NGO

B4. Working hours per shift:

☐ 8 hours

☐ 10 hours

☐ 12 hours

☐ Other: _______

B5. Night shifts per month: ______ nights

B6. Typical nurse-patient ratio:

☐1:10

☐ 1:15

☐ 1:20

☐ 1:25

☐ Other: _______

SECTION C: CONFLICT AND WORKPLACE EXPOSURES

C1. Displacement Status:

☐ Displaced (forced to relocate due to conflict)

☐ Non-displaced

C2. Monthly Income Adequacy:

☐ Adequate for living costs

☐ Inadequate for living costs

C3. Workplace Violence Exposure (past 6 months):

☐ Yes, experienced physical, verbal, or psychological aggression

☐ No

C4. Types of violence experienced (if yes to C3) -select all that apply:

☐ Physical

☐ Verbal

☐ Psychological

(Note: Participants could select multiple types. For analysis, any exposure to any type was coded as "exposed." Types are reported descriptively in Table S6.)

C5. Source(s) of violence (if yes to C3) -select all that apply:

☐ Patients

☐ Relatives

☐ Colleagues

(Note: Participants could select multiple sources. Sources are reported descriptively in Table S6.)

SECTION D: SUPPORT AND RESOURCES

D1. Access to Psychological Support:

☐ Yes, available through workplace

☐ No

(Note: This question assesses availability/accessibility of formal mental health support services, not utilisation, frequency, intensity, or quality of support received.)

SECTION E: PATIENT HEALTH QUESTIONNAIRE-9 (PHQ-9)

Instructions: Over the last 2 weeks, how often have you been bothered by any of the following problems?

**Item**	**Not at all (0)**	**Several days (1)**	**More than half the days (2)**	**Nearly every day (3)**
1. Little interest or pleasure in doing things	☐	☐	☐	☐
2. Feeling down, depressed, or hopeless	☐	☐	☐	☐
3. Trouble falling or staying asleep, or sleeping too much	☐	☐	☐	☐
4. Feeling tired or having little energy	☐	☐	☐	☐
5. Poor appetite or overeating	☐	☐	☐	☐
6. Feeling bad about yourself -or that you are a failure or have let yourself or your family down	☐	☐	☐	☐
7. Trouble concentrating on things, such as reading the newspaper or watching	☐	☐	☐	☐

television				
8. Moving or speaking so slowly that other people could have noticed? Or the opposite -being so fidgety or restless that you have been moving around a lot more than usual	☐	☐	☐	☐
9. Thoughts that you would be better off dead or of hurting yourself in some way	☐	☐	☐	☐

Total Score: ______ / 27

Severity Categories:

Minimal: 0–4

Mild: 5–9

Moderate: 10–14

Moderately severe: 15–19

Severe: 20–27

Cut-off for clinically significant depressive symptoms: ≥10

SECTION F: GENERALIZED ANXIETY DISORDER-7 (GAD-7)

Instructions: Over the last 2 weeks, how often have you been bothered by the following problems?

**Item**	**Not at all (0)**	**Several days (1)**	**More than half the days (2)**	**Nearly every day (3)**
1. Feeling nervous, anxious, or on edge	☐	☐	☐	☐
2. Not being able to stop or control worrying	☐	☐	☐	☐
3. Worrying too much about different things	☐	☐	☐	☐
4. Trouble relaxing	☐	☐	☐	☐
5. Being so restless that it's hard to sit still	☐	☐	☐	☐
6. Becoming easily annoyed or irritable	☐	☐	☐	☐
7. Feeling afraid as if something awful might happen	☐	☐	☐	☐

Total Score: ______ / 21

Severity Categories:

Minimal: 0–4

Mild: 5–9

Moderate: 10–14

Severe: 15–21

Cut-off for moderate-to-severe anxiety symptoms: ≥10

SECTION G: BURNOUT SINGLE-ITEM MEASURE

IMPORTANT NOTE: This single-item measure was selected for pragmatic reasons to minimise respondent burden in a highly stressed population. While it has demonstrated acceptable validity (r = 0.82 with the MBI-HSS emotional exhaustion subscale in pilot testing), it cannot capture the multidimensional nature of burnout (emotional exhaustion, depersonalisation, reduced personal accomplishment). Findings related to burnout -particularly the supplementary exploratory indirect association analyses -should be interpreted with caution and confirmed using validated multidimensional instruments (e.g., the full Maslach Burnout Inventory) in future research.

G1. How would you rate your level of burnout?

☐ None (0)

☐ Low (1)

☐ Moderate (2)

☐ High (3)

☐ Severe (4)

Burnout Coding for Analysis:

**Original Response**	**Collapsed Coding**
None (0)	Low (1)
Low (1)	Low (1)
Moderate (2)	Moderate (2)
High (3)	High (3)
Severe (4)	High (3)

"High Burnout" Definition: Burnout Level = 3 (original response High or Severe)

SECTION H: TURNOVER INTENTION

H1. Do you intend to leave your current nursing position within the next 12 months?

☐ Yes

☐ No

SECTION I: SAFETY PROTOCOL REMINDER (FOR RESEARCHERS ONLY)

IMPORTANT: If the participant endorses any non-zero response to PHQ-9 Item 9 (i.e., responding "Several days," "More than half the days," or "Nearly every day"), the following protocol must be implemented immediately:

1. Immediate Risk Assessment: Conduct a structured evaluation of suicidal ideation, intent, plan, and access to means.

2. Referral:

If at imminent risk: Immediately refer to the on-site psychiatrist or mental health professional (where available), or to the nearest emergency department or mental health facility.

Provide a written referral pathway with contact information for local mental health services and emergency contacts.

3. Escalation Protocol: In settings where immediate referral is not feasible, follow the pre-established escalation protocol coordinated with local health authorities and humanitarian partners.

4. Documentation: Document all actions taken.

All safety procedures have been reviewed and approved by the Institutional Review Board (IRB) prior to data collection (IRB Reference Number: YEM-IRB-2025-094).

PART 2: ARABIC VERSION -COMPLETE QUESTIONNAIRE

القسم أ: المعلومات الاجتماعية والديموغرافية

أ1. العمر: ______ سنة

أ2. الجنس:

☐ذكر

☐أنثى

أ3. الحالة الاجتماعية:

☐أعزب/عزباء

☐متزوج/ة

☐مطلق/ة

☐أرمل/ة

أ4. المستوى التعليمي:

☐دبلوم

☐بكالوريوس

☐ماجستير

☐دكتوراه

أ5. المحافظة الحالية________________ :

القسم ب: الخصائص المهنية

ب1. سنوات الخبرة في التمريض: ______ سنة

ب2. نوع التوظيف:

☐دائم

☐عقد

☐تطوع

ب3. نوع المنشأة:

☐حكومية

☐□ا□ة

☐منظمة غير حكومية

ب4. ساعات العمل في كل نوبة:

8 ☐ساعات

10 ☐ساعات

12 ☐ساعة

☐أ□رى: ....

ب5. عدد نوبات الليل الشهرية: ______ ليلة

ب6. نسبة الممرضين إلى المرضى النموذجية:

☐ **1:10**

☐ **1:15**

☐ **1:20**

☐ **1:25**

☐ أ□رى: ....

القسم ج: التعرض للصراع والعنف في مكان العمل

ج1. وضع النزوح:

☐نازح (اضطر للانتقال بسبب الصراع)

☐غير نازح

ج2. كفاية الد□ل الشهري:

☐كافية لتكاليف المعيشة

☐غير كافية لتكاليف المعيشة

ج3. التعرض للعنف في مكان العمل (□لال الستة أشهر الماضية):

☐نعم، تعرضت لعنف جسدي أو لفظي أو نفسي

☐لا

ج4. أنواع العنف الذي تعرضت له (إذا كانت الإجابة نعم على ج3) -□تر كل ما ينطبق:

☐جسدي

☐لفظي

☐نفسي

ج5. مصدر/مصادر العنف (إذا كانت الإجابة نعم على ج3) -□تر كل ما ينطبق:

☐المرضى

☐الأقارب

☐الزملاء

القسم د: الدعم والموارد

د1. الو□ول إلى الدعم النفسي**:**

☐نعم، متاح من□لال مكان العمل

☐لا

القسم هـ: استبيان □حة المريض-9(**PHQ-9**)

التعليمات: □لال الأسبوعين الماضيين، كم مرة شعرت بالانزعاج من أي من المشاكل التالية؟

**كل يوم تقريباً (3)**	**أكثر من نصف الأيام (2)**	**عدة أيام (1)**	**لا على الإطلاق (0)**	**البند**
☐	☐	☐	☐	1.قلة الاهتمام أو المتعة في فعل الأشياء
☐	☐	☐	☐	2.الشعور بالحزن أو الاكتئاب أو اليأس
☐	☐	☐	☐	3.□عوبة في النوم أو البقاء نائماً، أو النوم أكثر من اللازم
☐	☐	☐	☐	4.الشعور بالتعب أو قلة الطاقة
☐	☐	☐	☐	5.ضعف الشهية أو الأكل الزائد
☐	☐	☐	☐	6.الشعور بالسوء تجاه نفسك
☐	☐	☐	☐	7.□عوبة في التركيز على الأشياء
☐	☐	☐	☐	8.التحرك أو التحدث ببطء شديد، أو العكس
☐	☐	☐	☐	9.أفكار بأنك ستكون أفضل حالاً ميتاً أو بإيذاء نفسك بطريقة ما

المجموع الكلي: _______ / 27

فئات الشدة:

بسيط: 0–4

□فيف: 5–9

متوسط: 10–14

متوسط الشدة: 15–19

شديد: 20–27

عتبة أعراض الاكتئاب ذات الأهمية السريرية: ≥10

القسم و: مقياس اضطراب القلق العام-7(GAD-7)

التعليمات: □لال الأسبوعين الماضيين، كم مرة شعرت بالانزعاج من المشاكل التالية؟

**لا على الإطلاق (0)**	**عدة أيام (1)**	**أكثر من نصف الأيام (2)**	**كل يوم تقريباً (3)**	**البند**
☐	☐	☐	☐	1.الشعور بالعصبية أو القلق أو التوتر
☐	☐	☐	☐	2.عدم القدرة على التوقف أو التحكم في القلق
☐	☐	☐	☐	3.القلق الزائد حول أشياء مختلفة
☐	☐	☐	☐	4.□عوبة في الاستر□اء

5.الشعور بعدم الراحة لدرجة يصعب معها الجلوس	☐	☐	☐	☐
6.سرعة الانفعال أو الغضب	☐	☐	☐	☐
7.الشعور بالخوف كما لو أن شيئاً فظيعاً قد يحدث	☐	☐	☐	☐

المجموع الكلي: ______ / 21

فئات الشدة:

بسيط: 0–4

□فيف: 5–9

متوسط: 10–14

شديد: 15–21

عتبة أعراض القلق المتوسطة إلى الشديدة: ≥10

القسم ز: مقياس الاحتراق الوظيفي ذو البند الواحد

ملاحظة مهمة: تم ا□تيار هذا المقياس ذو البند الواحد لأسباب عملية لتقليل عبء المستجيب في population شديد الضغط. بينما أظهر □حة مقبولة (r = 0.82 مع مقياس MBI-HSS للإنهاك العاطفي في الا□تبار التجريبي)، إلا أنه لا يمكنه التقاط الطبيعة متعددة الأبعاد للاحتراق الوظيفي. يجب تفسير النتائج المتعلقة بالاحتراق الوظيفي -و□ا□ة تحليلات الارتباطات غير المباشرة الاستكشافية - بحذر.

ز1. كيف تقيم مستوى الاحتراق الوظيفي لديك؟

☐لا يوجد(0)

☐منخفض(1)

☐متوسط(2)

☐مرتفع(3)

☐شديد(4)

ترميز الاحتراق الوظيفي للتحليل:

**الترميز المدمج**	**الاستجابة الأصلية**
منخفض(1)	لا يوجد(0)
منخفض(1)	منخفض(1)
متوسط(2)	متوسط(2)
مرتفع(3)	مرتفع(3)
مرتفع(3)	شديد(4)

تعريف "الاحتراق الوظيفي المرتفع": مستوى الاحتراق = 3 (الاستجابة الأ□لية مرتفع أو شديد)

القسم ح: نية ترك العمل

ح1. هل تنوي ترك وظيفتك الحالية في التمريض □لال الاثني عشر شهراً القادمة؟

☐نعم

☐لا

القسم ط: تذكير بروتوكول السلامة (للباحثين فقط)

مهم: إذا أقر المشارك بأي استجابة غير فرية للبند 9 منPHQ-9 ، يجب تنفيذ البروتوكول التالي فوراً:

1.تقييم المخاطر الفوري: إجراء تقييم منظم للأفكار الانتحارية، النية، الخطة، والو ول إلى الوسائل.

2.الإحالة: إذا كان في طر وشيك، إحالة فورية إلى الطبيب النفسي أو أ صائي الصحة النفسية في الموقع، أو إلى أقرب قسم طوارئ.

3.بروتوكول التصعيد: في البيئات التي لا تكون فيها الإحالة الفورية ممكنة، اتبع بروتوكول التصعيد المحدد مسبقاً.

4.التوثيق: توثيق جميع الإجراءات المتخذة.

تمت مراجعة جميع إجراءات السلامة والموافقة عليها من قبل لجنة المراجعة المؤسسية (IRB) قبل جمع البيانات) رقم مرجع IRB: YEM-IRB-2025-094).

PART 3: INFORMED CONSENT FORM -ENGLISH

Title: **Psychological Distress Among Nurses in Conflict-Affected Yemen**

Principal Investigator: Dr. Naif Taleb Ali, PhD

Institution: Radfan College University, Lahej, Yemen

IRB Reference Number: YEM-IRB-2025-094

INTRODUCTION

You are invited to participate in a research study about mental health among nurses working in conflict-affected areas of Yemen. This study aims to understand the prevalence of depressive symptoms, anxiety symptoms, burnout, and factors associated with these conditions.

PROCEDURES

If you agree to participate, you will complete a questionnaire that will take approximately 20–30 minutes. The questionnaire includes questions about your background, work conditions, and mental health symptoms.

RISKS AND BENEFITS

Risks are minimal but may include temporary discomfort when answering sensitive questions. One of the questions (PHQ-9 Item 9) asks about thoughts of self-harm. If you endorse this item, a trained researcher will conduct a brief risk assessment and, if needed, provide you with a referral to mental health services. Benefits include contributing to knowledge that may improve working conditions for nurses in Yemen. All participants will receive information about mental health support services, including contact information for local mental health providers and emergency contacts.

CONFIDENTIALITY

Your responses are completely anonymous. No personally identifiable information will be collected. Data will be stored on encrypted servers and published only in aggregate form.

VOLUNTARY PARTICIPATION

Your participation is voluntary. You may refuse to participate or withdraw at any time without penalty.

CONTACT INFORMATION

For questions:

Dr. Naif Taleb Ali

Email: n.taleb@ust.edu

Phone: +967 772 571 390

For ethical concerns:

Radfan College University Ethical Committee

IRB Reference Number: YEM-IRB-2025-094

CONSENT STATEMENT

- ☐ I have read and understood the above information.
- ☐ I voluntarily agree to participate.
- ☐ I understand I can withdraw at any time without penalty.

Signature: ___________________________

Date: ______ / ______ / 2025

PART 4: INFORMED CONSENT FORM -ARABIC

العنوان: **الضيق النفسي بين الممرضين في اليمن المتأثر بالصراع**

الباحث الرئيسي: د. نايف طالب علي

المؤسسة: جامعة كلية ردفان، لحج، اليمن

رقم مرجع IRB: YEM-IRB-2025-094

مقدمة

أنت مدعو للمشاركة في دراسة بحثية حول الصحة النفسية بين الممرضين العاملين في المناطق المتأثرة بالصراع في اليمن. تهدف هذه الدراسة إلى فهم انتشار أعراض الاكتئاب، وأعراض القلق، والاحتراق الوظيفي، والعوامل المرتبطة بهذه الحالات.

الإجراءات

إذا وافقت على المشاركة، ستقوم بإكمال استبيان سيستغرق حوالي 20-30 دقيقة.

المخاطر والفوائد

المخاطر طفيفة ولكنها قد تشمل عدم الراحة المؤقتة عند الإجابة على أسئلة حساسة. أحد الأسئلة) البند 9 من (PHQ-9 يسأل عن أفكار إيذاء النفس. إذا أقررت بهذا البند، سيجري باحث مدرب تقييماً موجزاً للمخاطر، وإذا لزم الأمر، سيقدم لك إحالة إلى□دمات الصحة النفسية.

السرية

إجاباتك مجهولة تماماً. لن يتم جمع أي معلومات تعريف شخصية.

المشاركة الطوعية

مشاركتك طوعية. يمكنك رفض المشاركة أو الانسحاب في أي وقت دون عقوبة.

معلومات الاتصال

للأسئلة:

د. نايف طالب علي

البريد الإلكتروني: n.taleb@ust.edu

الهاتف: +967 772 571 390

للمخاوف الأ□لاقية:

لجنة الأ□لاقيات بجامعة كلية ردفان

رقم مرجع IRB: YEM-IRB-2025-094

بيان الموافقة

☐لقد قرأت وفهمت المعلومات الواردة أعلاه.

☐أوافق طواعية على المشاركة.

☐أفهم أنه يمكنني الانسحاب في أي وقت دون عقوبة.

التوقيع:...

PART 5: BURNOUT MEASURE -DETAILED DESCRIPTION AND VALIDATION

5.1 Instrument Description

Question: "How would you rate your level of burnout?"

Original Response Options:

Response	Code
None	0
Low	1
Moderate	2
High	3
Severe	4

None 0

Low 1

Moderate 2

High 3

Severe 4

Rationale: This pragmatic single-item measure was selected to minimise respondent burden in a highly stressed, conflict-affected population. Multi-item burnout instruments (e.g., the full Maslach Burnout Inventory) are lengthy (22 items) and may increase respondent burden and non-response in this context. The single-item approach has been previously validated in healthcare settings [39].

5.2 Validation Procedure

Pilot Test:

Sample: 40 nurses

Recruitment: From two facilities in Aden

Timing: January 16–19, 2025

Procedure: Participants simultaneously completed:

1. The single-item burnout measure

2. The MBI-Human Services Survey (MBI-HSS) emotional exhaustion subscale (Arabic version)

Statistical Analysis:

Method: Pearson's correlation coefficient

Assumption Check: Normal distribution of both variables confirmed (Shapiro-Wilk $p > 0.05$)

Result: $r = 0.82$ (95% CI: 0.71–0.89, $p < 0.001$)

Interpretation: The single-item measure demonstrated strong convergent association with the MBI-HSS emotional exhaustion subscale. This provides confidence in its use as a proxy for overall severity of burnout.

5.3 Limitations

1. Multidimensionality: The single-item measure cannot capture the multidimensional nature of burnout:

Emotional exhaustion

Depersonalisation

Reduced personal accomplishment

2. Conceptual Overlap: There is conceptual overlap between burnout (particularly emotional exhaustion) and depressive symptoms, which complicates interpretation [34,35].

3. Supplementary Analyses: The supplementary exploratory indirect association and effect modification analyses involving burnout should be interpreted with extreme caution due to these limitations.

5.4 Coding for Analysis

**Original Response**	**Collapsed Coding**
None (0)	Low (1)
Low (1)	Low (1)
Moderate (2)	Moderate (2)
High (3)	High (3)
Severe (4)	High (3)

"High Burnout" Definition: Burnout Level = 3 (original response High or Severe)

Prevalence: 34.0% (n = 272)

Prespecified: Yes (File S1)

Consistency: This coding was used consistently across all analyses:

Ordinal logistic regression

Turnover intention model

SEM analyses

Data Dictionary: File S6

PART 6: PHQ-9 ITEM 9 SAFETY PROTOCOL (REMINDER)

(Detailed in Section 7.3 of File S1)

Trigger: Any non-zero response to PHQ-9 Item 9

Actions:

1. Immediate risk assessment by trained nurse researcher

2. Structured evaluation of:

Suicidal ideation

Intent

Plan

Access to means

3. Referral to:

On-site psychiatrist or mental health professional (where available), OR

Nearest emergency department or mental health facility

4. Written referral pathway provided

5. Escalation protocol followed in settings where immediate referral is not feasible

6. Documentation of all actions

IRB Approval: All safety procedures reviewed and approved by IRB prior to data collection (IRB Reference Number: YEM-IRB-2025-094)

Adverse Events: None reported during the study period

PART 7: REFERENCES

34. Bianchi, R., & Brisson, R. (2019). Burnout and depression: Causal attributions and construct overlap. Journal of Health Psychology, 24(11), 1574–1580. https://doi.org/10.1177/1359105317740415
35. Bianchi, R., Schonfeld, I. S., & Laurent, E. (2015). Burnout-depression overlap: A review. Clinical Psychology Review, 36, 28–41. https://doi.org/10.1016/j.cpr.2015.01.004
39. Dolan, E. D., Mohr, D., Lempa, M., Joos, S., Fihn, S. D., Nelson, K. M., & Helfrich, C. D. (2015). Using a single item to measure burnout in primary care staff: A psychometric evaluation. Journal of General Internal Medicine, 30(5), 582–587. https://doi.org/10.1007/s11606-014-3112-6
