# Supplementary material for: Mental Health, Burnout, and Turnover Intention Among Nurses in Conflict-Affected Yemen: A Multicenter Study Across Eight Accessible Governorates
Source: Nurs Rep. 2026 Oct 7;16(10):353. doi: 10.3390/nursrep16100353 (PMC13708594; doi:10.3390/nursrep16100353)
Supplement: Supplementary file 1 [file nursrep-16-00353-s001.zip › File S3 Reporting Checklists.pdf]

# File S3: Reporting Checklists

## S3.1 STROBE Checklist (Cross-Sectional Studies)

| Section | Item | Check | Where Reported |
|---|---|---|---|
| Title & Abstract | 1. Indicate study design in title/abstract | ✓ | Title, Abstract |
| | 2. Provide informative summary in abstract | ✓ | Abstract |
| Introduction | 3. Explain scientific background/rationale | ✓ | Introduction |
| | 4. State specific objectives/hypotheses | ✓ | Introduction |
| Methods | 5. Describe study setting, locations, dates | ✓ | Methods |
| | 6. Eligibility criteria for participants | ✓ | Methods |
| | 7. Sources and methods of participant selection | ✓ | Methods |
| | 8. Clearly define all outcomes, exposures, predictors | ✓ | Methods |
| | 9. Describe data sources/measurement methods | ✓ | Methods |
| | 10. Describe efforts to address potential bias | ✓ | Methods (QC) |
| | 11. Explain sample size determination | ✓ | Methods 2.3.1, File S4 (Table S9) |
| | 12. Describe statistical methods | ✓ | Methods 3.10, File S8 |
| Results | 13. Report participant numbers at each stage | ✓ | Results, Fig S3 |
| | 14. Characteristics of study participants | ✓ | Table 1 |
| | 15. Report numbers with missing data | ✓ | Methods 3.11, File S4 |
| | 16. Report main results with estimates, precision | ✓ | Tables 2-3 |

| | | | |
|---|---|---|---|
| | 17. Report other analyses done | ✓ | Results, Tables S1-S7 |
| Discussion | 18. Summarise key results | ✓ | Discussion |
| | 19. Discuss limitations | ✓ | Limitations |
| | 20. Provide interpretation consistent with results | ✓ | Discussion |
| | 21. Discuss generalisability | ✓ | Discussion, Limitations |
| Other | 22. Funding sources | ✓ | Declarations |
| | 23. Data availability statement | ✓ | Declarations |

## S3.2 SAGER Guidelines Checklist

| Section | Recommendation | Implemented | Location |
|---|---|---|---|
| Title & Abstract | 1. Indicate if findings apply to one sex only | ✓ | Abstract (both sexes included) |
| Introduction | 2. Report if sex/gender differences expected | ✓ | Introduction |
| Methods | 3. Report how sex/gender considered in design | ✓ | Methods |
| | 4. Provide details of sex/gender-based analyses | ✓ | Methods, File S8 |
| Results | 5. Report participant numbers by sex/gender | ✓ | Table 1 |
| | 6. Present data disaggregated by sex/gender | ✓ | Table S3, File S8 |
| | 7. Report sex-specific results if found | ✓ | Results, Discussion |
| Discussion | 8. Discuss implications of sex/gender differences | ✓ | Discussion |
| | 9. Address limitations of sex/gender analysis | ✓ | Limitations |

## S3.3 Data Quality Assurance Checklist

| Phase | Quality Control Measure | Implemented | Documentation |
|---|---|---|---|
| Design | 1. Instrument validation (CVI ≥0.80) | ✓ | Methods, File S2 |
| | 2. Pilot testing (n=40) | ✓ | Methods |
| | 3. Cognitive interviewing | ✓ | Methods |
| Collection | 4. Standardised RA training (5 days) | ✓ | Methods |
| | 5. Daily validation checks (10%) | ✓ | Methods |
| | 6. Real-time range/consistency checks | ✓ | Methods |
| | 7. Weekly data review meetings | ✓ | Methods |
| Processing | 8. Automated data cleaning scripts | ✓ | File S6 |
| | 9. Missing data assessment (<3%) | ✓ | File S8 |
| | 10. Outlier detection and handling | ✓ | File S8 |
| Analysis | 11. Multicollinearity checks (VIF<5) | ✓ | File S8 |
| | 12. Model assumption verification | ✓ | File S8 |
| | 13. Sensitivity analyses completed | ✓ | File S8 |
| | 14. Code reproducibility verified | ✓ | File S6 |

## S3.4 Conflict Research Ethics Checklist

| Principle | Recommendation | Implemented | Location |
|---|---|---|---|
| **Ethical Approval** | | | |
| | 1. IRB approval obtained from local institution | ✓ | File S9 |
| | 2. IRB approval obtained prior to data collection | ✓ | Methods 2.1, File S9 |
| | 3. Approval covers all study sites | ✓ | File S9 |
| **Informed Consent** | | | |

| Principle | Recommendation | Implemented | Location |
|---|---|---|---|
| | 4. Written informed consent obtained | ✓ | Methods 2.1, File S2 |
| | 5. Consent forms in local language (Arabic) | ✓ | File S2 |
| | 6. Consent forms in English | ✓ | File S2 |
| | 7. Right to withdraw without penalty explained | ✓ | File S2 |
| **Safety and Protection** | | | |
| | 8. Safety protocol for PHQ-9 Item 9 (self-harm) | ✓ | Methods 3.1, File S2 |
| | 9. Immediate risk assessment for any non-zero Item 9 response | ✓ | Methods 3.1, File S2 |
| | 10. Referral pathway for at-risk participants | ✓ | Methods 3.1, File S2 |
| | 11. Escalation protocol for conflict-affected settings | ✓ | Methods 3.1, File S2 |
| | 12. No adverse events reported | ✓ | Methods 3.1 |
| **Confidentiality** | | | |
| | 13. Data anonymised at point of collection | ✓ | Methods 3.9 |
| | 14. No personally identifiable information collected | ✓ | Methods 3.9, File S2 |
| | 15. Data stored on encrypted servers | ✓ | Methods 3.9 |
| | 16. Published only in aggregate form | ✓ | Methods 3.9 |
| **Researcher Safety** | | | |
| | 17. Security assessments conducted with local authorities | ✓ | Methods 2.3, File S10 |
| | 18. Facilities under siege or closed excluded | ✓ | Methods 2.3, File S10 |
| | 19. Research team trained in psychological first aid | ✓ | Methods 3.9 |
| | 20. Regular debriefing sessions for research team | ✓ | Methods 3.9 |
| **Community Engagement** | | | |
| | 21. Local health authorities consulted | ✓ | Methods 2.3 |
| | 22. Humanitarian partners (Health Cluster) consulted | ✓ | Methods 2.3 |
| | 23. Findings to be disseminated to participating facilities | ✓ | Public Involvement Statement |

| Principle | Recommendation | Implemented | Location |
|---|---|---|---|
| **Vulnerability Considerations** | | | |
| | 24. Population vulnerability acknowledged | ✓ | Methods, Discussion |
| | 25. Measures to minimise respondent burden | ✓ | Methods 3.2 |
| | 26. Psychological support information provided to all participants | ✓ | Methods 3.8 |

**Note:** This checklist was developed based on ethical guidance for research in conflict-affected settings, including the WHO Ethical and Safety Recommendations for Researching, Documenting and Monitoring Sexual Violence in Emergencies, and the Declaration of Helsinki.
