# Supplementary material for: Mental Health, Burnout, and Turnover Intention Among Nurses in Conflict-Affected Yemen: A Multicenter Study Across Eight Accessible Governorates
Source: Nurs Rep. 2026 Oct 7;16(10):353. doi: 10.3390/nursrep16100353 (PMC13708594; doi:10.3390/nursrep16100353)
Supplement: Supplementary file 1 [file nursrep-16-00353-s001.zip › File S4 Sensitivity Analyses.pdf]

FILE S4 SENSITIVITY ANALYSES

Study Title: **Mental Health, Burnout and Turnover Intention among Nurses in Conflict-Affected Yemen: A Multicentre Study Across Eight Accessible Governorates**

TABLE OF CONTENTS

Table	Content
Table S1	Multiple Imputation Analysis
Table S2a	Predicted Probabilities and Risk Differences for Key Exposures
Table S2b	Risk Ratios from Poisson Regression with Robust Variance
Table S2c	Turnover Intention Model with Mental Health Variables
Table S3	Alternative Cut-offs Sensitivity Analysis
Table S4	Outlier Sensitivity Analysis
Table S5	Harman's Single-Factor Test
Table S6	Multicollinearity Diagnostics (VIF)
Table S7	Influence Diagnostics (Cook's Distance, DFBETAS)
Table S8	Effect Size Interpretation (Cohen's f^2)
Table S9	Sampling Details by Governorate
Table S10	Non-Significant and Borderline Findings
Table S11	Sampling-Weighted vs. Unweighted Prevalence Estimates for Principal Outcomes

1. Table S1. MULTIPLE IMPUTATION ANALYSIS

Method: Multiple Imputation by Chained Equations (MICE)

- Number of imputations: m = 20
- Iterations: 10
- Algorithm: Predictive Mean Matching (PMM)
- Software: R (mice package)

Variables Imputed:

Variable	Missingness (%)	Imputation Method
PHQ-9 Score	1.2%	PMM
GAD-7 Score	1.5%	PMM
Burnout Level	2.1%	PMM
Turnover Intention	0.9%	Logistic
Displacement Status	1.8%	Logistic
Workplace Violence	2.3%	Logistic
Income Adequacy	2.0%	Logistic
Nurse-Patient Ratio	1.6%	PMM

Results:

Predictor	Complete-Case AOR (95% CI)	Imputed AOR (95% CI)	Absolute Difference
Displaced	2.31 (1.68–3.17)	2.29 (1.67–3.15)	0.02

Workplace Violence	2.84 (2.03–3.97)	2.82 (2.02–3.94)	0.02
Inadequate Income	2.53 (1.80–3.56)	2.51 (1.79–3.53)	0.02
Nurse-Patient Ratio ≥1:20	1.68 (1.07–2.63)	1.67 (1.06–2.61)	0.01
No Psychological Support	1.82 (1.30–2.54)	1.80 (1.29–2.52)	0.02
Night Shifts (per shift)	1.26 (1.17–1.36)	1.25 (1.16–1.35)	0.01

Conclusion: Results from multiple imputation were nearly identical to complete-case analysis, with absolute differences ranging from 0.01 to 0.02. Missing data did not materially influence the findings.

Table S2a. Predicted Probabilities and Risk Differences for Key Exposures

Exposure	Predicted Probability (Unexposed)	Predicted Probability (Exposed)	Risk Difference (pp)	NNH (1/RD)
Workplace Violence	31%	56%	25	4.0
Displacement	36%	56%	20	5.0
Inadequate Income	34%	58%	24	4.2
Nurse-Patient Ratio (1:25 vs 1:10)	35%	60%	25	4.0
No Psychological Support	38%	57%	19	5.3

Notes:

• Predicted probabilities were calculated from mixed-effects logistic regression models using marginal standardisation, setting all other covariates to their means.

• Risk differences were computed as the difference in predicted probabilities between exposed and unexposed participants.

• NNH = Number Needed to Harm (1 / Risk Difference).

• pp = percentage points.

Table S2b. Risk Ratios from Poisson Regression with Robust Variance

Predictor	Depressive Symptoms RR (95% CI)	p-value	Anxiety Symptoms RR (95% CI)	p-value
Displaced	1.55 (1.32–1.82)	<0.001	1.48 (1.27–1.73)	<0.001
Workplace Violence	1.72 (1.45–2.04)	<0.001	1.65 (1.40–1.95)	<0.001
Inadequate Income	1.61 (1.36–1.91)	<0.001	1.57 (1.33–1.85)	<0.001
Nurse-Patient Ratio ≥1:20	1.32 (1.12–1.56)	0.001	1.28 (1.09–1.50)	0.003
No Psychological Support	1.41 (1.20–1.66)	<0.001	1.38 (1.18–1.61)	<0.001
Night Shifts (per shift)	1.09 (1.06–1.12)	<0.001	1.08 (1.05–1.11)	<0.001

Notes:

• RR = Risk Ratio.

• Risk ratios were estimated using Poisson regression with robust variance [18].

• Poisson regression with robust variance was selected over log-binomial regression because log-binomial models frequently fail to converge when outcomes are common.

• Risk ratios are preferred over odds ratios for clinical and policy interpretation when outcomes are common [19,17].

Table S2c. Turnover Intention Model with Mental Health Variables

Predictor	Model 1 (Without Mental Health) AOR (95% CI)	p-value	Model 2 (With Mental Health) AOR (95% CI)	p-value
Displaced	1.95 (1.42–2.68)	<0.001	1.52 (1.08–2.14)	0.016
Inadequate Income	2.12 (1.54–2.92)	<0.001	1.78 (1.26–2.51)	0.001
Workplace Violence	2.35 (1.70–3.25)	<0.001	1.82 (1.28–2.59)	<0.001
No Psychological Support	1.58 (1.15–2.17)	0.005	1.32 (0.94–1.85)	0.108
Nurse-Patient Ratio ≥1:20	1.62 (1.18–2.22)	0.003	1.38 (0.98–1.94)	0.064
Mental Health Variables				
Depressive Symptoms (PHQ-9 ≥ 10)	-	-	3.42 (2.48–4.71)	<0.001
Anxiety Symptoms (GAD-7 ≥ 10)	-	-	2.88 (2.10–3.95)	<0.001
High Burnout	-	-	4.15 (2.97–5.80)	<0.001

Notes:

• Model 1 estimates overall associations between exposures and turnover intention without adjustment for mental health variables.

• Model 2 estimates associations conditional on psychological distress.

• Because mental health variables may lie on the same conceptual pathway as turnover intention, their inclusion substantially attenuates the estimated associations between exposures and turnover intention.

• AOR = Adjusted Odds Ratio; CI = Confidence Interval.

• - indicates variable not included in that model.

2. ALTERNATIVE CUT-OFFS SENSITIVITY ANALYSIS

Table S3. Sensitivity Analysis with Alternative Cut-offs for PHQ-9 and GAD-7

Cut-off	Displaced AOR (95% CI)	Workplace Violence AOR (95% CI)	Inadequate Income AOR (95% CI)
Depressive Symptoms (PHQ-9)			
PHQ-9 ≥ 5	1.82 (1.40–2.37)***	2.15 (1.65–2.80)***	2.05 (1.57–2.68)***
PHQ-9 ≥ 10 (main)	2.31 (1.68–3.17)***	2.84 (2.03–3.97)***	2.53 (1.80–3.56)***
PHQ-9 ≥ 15	2.65 (1.85–3.80)***	3.12 (2.14–4.55)***	2.78 (1.91–4.05)***

Anxiety Symptoms (GAD-7)			
GAD-7 ≥ 5	1.95 (1.45–2.62)***	2.32 (1.75–3.08)***	2.18 (1.63–2.91)***
GAD-7 ≥ 8	2.05 (1.52–2.76)***	2.48 (1.83–3.36)***	2.28 (1.68–3.09)***
GAD-7 ≥ 10 (main)	2.18 (1.60–2.97)***	2.67 (1.94–3.67)***	2.41 (1.75–3.32)***

Notes:

• *** $p < 0.001$.

• Key associations remained significant across all thresholds.

• Effect sizes were slightly attenuated at lower severity cut-offs, as expected.

• Main analysis cut-offs: PHQ-9 ≥ 10 and GAD-7 ≥ 10.

Conclusion: The direction and significance of key associations were robust across alternative cut-offs, though effect sizes were slightly attenuated at lower severity thresholds.

3. OUTLIER SENSITIVITY ANALYSIS

Table S4. Sensitivity Analysis Excluding Extreme Cases

Predictor	Main Analysis (n=800) AOR (95% CI)	Excluding PHQ-9 ≥20 (n=720) AOR (95% CI)	Excluding GAD-7 ≥15 (n=656) AOR (95% CI)
Depressive Symptoms (PHQ-9 ≥ 10)			
Displaced	2.31 (1.68–3.17)***	2.28 (1.64–3.18)***	2.25 (1.60–3.15)***
Workplace Violence	2.84 (2.03–3.97)***	2.79 (1.97–3.95)***	2.75 (1.92–3.93)***
Inadequate Income	2.53 (1.80–3.56)***	2.48 (1.74–3.53)***	2.45 (1.71–3.51)***
Nurse-Patient Ratio ≥1:20	1.68 (1.07–2.63)*	1.52 (0.97–2.38)	1.58 (1.01–2.47)*
Anxiety Symptoms (GAD-7 ≥ 10)			
Displaced	2.18 (1.60–2.97)***	2.15 (1.56–2.96)***	2.12 (1.52–2.94)***
Workplace Violence	2.67 (1.94–3.67)***	2.62 (1.88–3.65)***	2.58 (1.82–3.63)***
Inadequate Income	2.41 (1.75–3.32)***	2.36 (1.70–3.28)***	2.33 (1.67–3.25)***
Nurse-Patient Ratio ≥1:20	1.62 (1.05–2.51)*	1.48 (0.94–2.33)	1.53 (0.98–2.39)

Notes:

• * $p < 0.05$; *** $p < 0.001$.

• Results remained consistent with the main analysis.

• Nurse-patient ratio ≥1:20 was significant for depressive symptoms in the main analysis (AOR = 1.68, $p = 0.024$) but borderline when excluding nurses with severe depressive symptoms (AOR = 1.52, $p = 0.068$).

• Outliers did not disproportionately influence the main conclusions.

4. COMMON METHOD BIAS ASSESSMENT

Table S5. Harman's Single-Factor Test

Method: Harman's single-factor test was conducted using exploratory factor analysis with all continuous items (PHQ-9, GAD-7, and burnout items).

Factor	Eigenvalue	% Variance Explained	Cumulative %
1	5.68	28.4%	28.4%
2	3.24	16.2%	44.6%
3	2.87	14.4%	59.0%
4	2.15	10.8%	69.8%
5	1.89	9.5%	79.3%
6+	-	20.7%	100%

Interpretation: The first unrotated factor accounted for 28.4% of the total variance. This result is reported descriptively only; it does not rule out common method variance, particularly because the principal exposures and outcomes are self-reported within the same questionnaire. The study design and measurement limitations themselves should be considered the primary safeguards against common method bias.

Note on Benford's Law: Benford's Law analysis was not conducted because PHQ-9 and GAD-7 scores are bounded integers with structurally determined first-digit distributions, making Benford's Law an invalid test for data manipulation in this context.

5. MULTICOLLINEARITY DIAGNOSTICS

Table S6. Variance Inflation Factors (VIF)

Variable	VIF	1/VIF
Displacement Status	1.42	0.704
Monthly Income Adequacy	1.38	0.725
Workplace Violence	1.35	0.741
Nurse-Patient Ratio	1.28	0.781
Night Shifts	1.22	0.820
Age	1.18	0.847
Gender	1.12	0.893
Employment Type	1.09	0.917
Mean VIF	1.26	

Conclusion: All VIF values were well below the threshold of 5, indicating no problematic multicollinearity.

6. INFLUENCE DIAGNOSTICS

Table S7. Influence Diagnostics

Diagnostic	Maximum Value	Threshold	Influential Observations
Cook's Distance	0.042	4/(800-10) = 0.005	18 (1.0%)
DFBETAS (Displaced)	0.32	1.0	None
DFBETAS (Violence)	0.28	1.0	None
DFBETAS (Income)	0.25	1.0	None

Notes:

• Cook's distance threshold was set at 4/(n-k) = 4/790 = 0.005, as recommended by Cook & Weisberg (1982) [16].

• DFBETAS threshold was set at 1.0.

• Excluding influential observations did not materially change the estimates.

Conclusion: No observations had undue influence on the main findings.

7. EFFECT SIZE INTERPRETATION

Table S8. Effect Sizes (Cohen's f^2) for Continuous Outcomes

Given the large sample size (n = 800), even small associations may reach statistical significance. To assess practical significance, we calculated Cohen's f^2 for each predictor in the linear regression models.

Predictor	Cohen's f^2 (Depressive Symptoms)	Cohen's f^2 (Anxiety Symptoms)	Interpretation
Workplace Violence	0.12	0.11	Small to Medium
Displacement	0.09	0.08	Small
Inadequate Income	0.10	0.09	Small
Nurse-Patient Ratio (1:25)	0.08	0.07	Small
Night Shifts (per shift)	0.04	0.04	Small

Interpretation: While most predictors were statistically significant, the effect sizes (Cohen's f^2) were predominantly small. This indicates that although the associations are statistically robust, their practical importance should be interpreted cautiously. The strongest predictors (workplace violence, displacement, inadequate income) showed small-to-medium effects, suggesting that interventions targeting multiple risk factors simultaneously may yield clinically meaningful reductions in distress, even if individual effects are modest.

Note: Cohen's f^2 thresholds: 0.02 = small, 0.15 = medium, 0.35 = large.

8. SAMPLING DETAILS BY GOVERNORATE

Table S9. Sampling Details by Governorate and Health Facility (N = 800)

Governorate	Region	Facilities Selected	Initial Approached	Non-Participants	Replacements	Total Approached	Participants	Response Rate (%)
Aden	South	3	113	13	13	126	100	79.4
Lahj	South	3	109	9	9	118	100	84.7
Abyan	South	3	107	7	7	114	100	87.7
Shabwah	Central	3	113	13	13	126	100	79.4
Hadramaw	East	3	104	4	4	108	100	92.6

t								
Al-Mahra	East	3	109	9	9	118	100	84.7
Socotra	East	3	109	9	9	118	100	84.7
Taiz	Central	3	108	8	8	116	100	86.2
Total	—	24	872	72	72	944	800	84.7

Note: The initial systematic random sampling sequence approached 872 eligible nurses. Of these, 72 did not participate (42 declined, 18 were on emergency duty, and 12 were administratively unavailable) and were replaced by the next eligible nurses on the sampling list, resulting in 72 additional nurses being approached. The total number of nurses approached was therefore 944 (872 + 72), of whom 800 participated, yielding an overall response rate of 84.7% (800/944).

Because equal allocation (~100 nurses per governorate) was used, selection probabilities differed across governorates. The primary analyses did not incorporate sampling weights; this is acknowledged as a limitation. Sampling-weighted prevalence estimates are presented in Table S12 and Section 9.8 of File S8.

9. REPORTING OF NON-SIGNIFICANT FINDINGS

Table S10. Non-Significant and Borderline Findings

To address potential reporting bias, we explicitly report all non-significant findings from the main analyses.

Predictor	Outcome	AOR (95% CI)	p-value
Nurse-Patient Ratio 1:15	Depressive Symptoms	1.30 (0.85–2.00)	0.226
Nurse-Patient Ratio 1:15	Anxiety Symptoms	1.28 (0.84–1.95)	0.247
Nurse-Patient Ratio 1:15	Turnover Intention	1.22 (0.80–1.86)	0.358
Age (per year)	Depressive Symptoms	0.98 (0.96–1.00)	0.051
Age (per year)	Anxiety Symptoms	0.99 (0.97–1.00)	0.051
Age (per year)	Turnover Intention	0.99 (0.97–1.00)	0.051
Contract Employment	Depressive Symptoms	1.29 (0.94–1.77)	0.117
Contract Employment	Anxiety Symptoms	1.18 (0.87–1.60)	0.295
Contract Employment	Turnover Intention	1.22 (0.89–1.67)	0.214
Facility Type (Public)	Depressive Symptoms	Not retained	N/A
Facility Type (Public)	Anxiety Symptoms	Not retained	N/A
Facility Type (Public)	Turnover Intention	Not retained	N/A

Interpretation: The non-significant findings for facility type, employment type, and moderate nurse-patient ratios (1:15) suggest a “homogenising effect” of protracted conflict, where systemic conflict-related stressors overwhelm institutional and contractual differences. These findings are discussed in the main manuscript and should be interpreted alongside the significant findings for a balanced understanding of the determinants of psychological distress among nurses in conflict settings.

Note: AOR = Adjusted Odds Ratio; CI = Confidence Interval.

Table S11. Sampling-Weighted vs. Unweighted Prevalence Estimates for Principal Outcomes

Outcome	Unweighted % (95% CI)	Weighted % (95% CI)	Difference (pp)
Depression (PHQ-9 ≥	52.0 (48.5–55.5)	52.4 (48.9–55.9)	+0.4

Outcome	Unweighted % (95% CI)	Weighted % (95% CI)	Difference (pp)
10)			
Anxiety (GAD-7 ≥ 10)	47.0 (43.5–50.5)	47.5 (44.0–51.0)	+0.5
High Burnout	34.0 (30.7–37.4)	33.6 (30.3–36.9)	−0.4
Turnover Intention	54.8 (51.2–58.3)	55.1 (51.5–58.7)	+0.3

Notes:

Weighted estimates account for differential selection probabilities across governorates using inverse-probability weights.
Population denominators were obtained from the Ministry of Public Health and Population (MoPHP) and Health Cluster Yemen.
For population-level interpretation, weighted estimates should be preferred.
The close agreement between weighted and unweighted estimates (absolute differences < 1 percentage point) strengthens confidence in the findings.

pp = percentage points.

10. SUMMARY OF SENSITIVITY ANALYSES

Analysis	Key Finding	Interpretation
Multiple Imputation	Results nearly identical to complete-case analysis (differences 0.01–0.02)	Missing data did not materially influence findings
Alternative Cut-offs	Key associations remained significant across all thresholds	Findings robust to alternative cut-off specifications
Outlier Sensitivity	Results consistent with main analysis	Outliers did not disproportionately influence conclusions
Harman's Test	First factor accounted for 28.4% of variance	Reported descriptively only; does not rule out common method variance
Multicollinearity (VIF)	Mean VIF = 1.26 (all < 5)	No problematic multicollinearity
Influence Diagnostics	18 observations (1.0%) with high Cook’s distance	Excluding influential observations did not change estimates
Effect Sizes	Small to medium effects (Cohen’s f^2 = 0.04–0.12)	Associations statistically robust but modest in magnitude
Sampling Details	24 facilities, 8 governorates, response rate 91.7%	Findings generalisable to accessible areas only
Non-Significant Findings	Reported for facility type, employment type, 1:15 ratio	Addresses potential reporting bias

11. LIMITATIONS OF SENSITIVITY ANALYSES

1. Cross-Sectional Design: The most fundamental limitation. Temporal precedence cannot be established; the observed associations may reflect reverse causality or bidirectional relationships.

2. Single-Item Burnout Measure: The use of a single-item burnout measure cannot capture the multidimensional nature of burnout (emotional exhaustion, depersonalisation, reduced personal accomplishment).

3. Multiple Testing: The exploratory analyses involved multiple tests without adjustment for multiple comparisons, increasing the risk of Type I errors.

4. Sampling Weights: The primary analyses did not incorporate sampling weights because equal allocation meant that selection probabilities differed across governorates. Sampling-weighted sensitivity analyses showed consistent direction of associations, though magnitude varied.

5. Self-Report Measures: All exposures and outcomes were self-reported, which may introduce social desirability and recall bias.

Recommendation: These findings should be considered hypothesis-generating. Future research should employ longitudinal designs with validated multidimensional instruments to confirm the robustness of these findings.

12. REFERENCES

16. Cook, R. D., & Weisberg, S. (1982). Residuals and Influence in Regression. Chapman & Hall.
17. Norton, E. C., Dowd, B. E., & Maciejewski, M. L. (2018). Odds Ratios-Current Best Practice and Use. JAMA, 320(1), 84-85.
19. Zhang, J., & Yu, K. F. (1998). What's the relative risk? A method of correcting the odds ratio in cohort studies of common outcomes. JAMA, 280(19), 1690-1691.
18. Zou, G. (2004). A modified Poisson regression approach to prospective studies with binary data. American Journal of Epidemiology, 159(7), 702-706.
