# Supplementary material for: Mental Health, Burnout, and Turnover Intention Among Nurses in Conflict-Affected Yemen: A Multicenter Study Across Eight Accessible Governorates
Source: Nurs Rep. 2026 Oct 7;16(10):353. doi: 10.3390/nursrep16100353 (PMC13708594; doi:10.3390/nursrep16100353)
Supplement: Supplementary file 1 [file nursrep-16-00353-s001.zip › File S5 supplementary analyses-222.pdf]

FILE S5: Supplementary Figures and Analyses

Complete Version

S1. Supplementary Mediation and Moderation Analyses

IMPORTANT CAVEAT: The following analyses are strictly exploratory and hypothesis-generating. Given the cross-sectional design and the inherent limitations of the single-item burnout measure, these findings should be interpreted with extreme caution. They are presented to generate hypotheses for future research and should not be used to draw causal conclusions or inform clinical practice changes without further validation. Robust confirmation requires replication using validated multidimensional burnout instruments (e.g., the full Maslach Burnout Inventory) in longitudinal cohort designs.

S1.1 Mediation by Burnout

Objective: To examine whether the data are statistically consistent with burnout mediating the relationship between conflict-related exposures (workplace violence, displacement) and psychological distress (depression, anxiety). This is an exploratory, hypothesis-generating analysis.

Method: Path analysis using structural equation modeling (SEM) was conducted. The model tested direct and indirect pathways from conflict exposures to psychological distress through burnout. The SEM model was estimated using robust maximum likelihood (MLR) estimation to account for non-normality. Given the binary nature of the outcome variables, a weighted least squares mean and variance adjusted (WLSMV) estimator was used for models with binary outcomes. Model fit was evaluated using CFI, TLI, RMSEA, and SRMR, with acceptable fit defined as CFI ≥ 0.90, TLI ≥ 0.90, RMSEA ≤ 0.08, and SRMR ≤ 0.08.

Results:

Direct Effects:

Path	β (Standardised)	95% CI Lower	95% CI Upper	p-value
Workplace Violence → Burnout	0.32	0.25	0.39	<0.001
Displacement → Burnout	0.28	0.21	0.35	<0.001
Burnout → Depression	0.42	0.35	0.49	<0.001
Burnout → Anxiety	0.38	0.31	0.45	<0.001
Workplace Violence → Depression (Direct)	0.25	0.18	0.32	<0.001
Displacement → Anxiety (Direct)	0.22	0.15	0.29	<0.001

Indirect Effects (Mediation):

Path	Indirect Effect (β)	95% CI Lower	95% CI Upper	% Mediated	p-value

Path	Indirect Effect (β)	95% CI Lower	95% CI Upper	% Mediated	p-value
Workplace Violence → Burnout → Depression	0.18	0.12	0.25	42.3%	<0.001
Displacement → Burnout → Anxiety	0.15	0.09	0.21	38.7%	<0.001

Model Fit Indices:

Fit Index	Value	Acceptable Threshold
CFI	0.92	≥0.90
TLI	0.90	≥0.90
RMSEA	0.06	≤0.08
RMSEA 90% CI	0.04–0.08	-
SRMR	0.05	≤0.08

Interpretation: The SEM model showed acceptable fit, suggesting that the data are reasonably consistent with the proposed model. Burnout statistically accounted for 42.3% of the association between workplace violence and depression, and 38.7% of the association between displacement and anxiety.

Important Note: These findings are associational, not causal. The cross-sectional design precludes causal interpretation of the mediation model. Definitive evidence requires prospective longitudinal studies or intervention trials designed to establish temporal precedence.

S1.2 Moderation by Psychological Support

Objective: To examine whether access to psychological support moderates the relationship between conflict exposures (displacement, workplace violence) and psychological distress (depression, anxiety).

Method: Interaction terms between psychological support and each conflict exposure were added to the fully adjusted models. Simple slopes analysis was conducted to examine the conditional effects of exposures at different levels of psychological support.

Results:

Path	β (Interaction)	95% CI Lower	95% CI Upper	p-value
Displacement × Psychological Support → Depression	-0.23	-0.32	-0.14	<0.001
Workplace Violence × Psychological Support → Depression	-0.19	-0.28	-0.10	<0.001

Simple Slopes Analysis:

Exposure	Psychological Support	β	p-value

Exposure	Psychological Support	β	p-value
Displacement → Depression	Available	0.18	0.012
Displacement → Depression	Not Available	0.41	<0.001
Workplace Violence → Depression	Available	0.22	0.008
Workplace Violence → Depression	Not Available	0.44	<0.001

Interpretation: The association between conflict exposures and depressive symptoms differed according to reported availability of workplace psychological support. The association between displacement and depression was weaker among nurses who reported that psychological support was available ($\beta = 0.18$, $p = 0.012$) compared to those who reported no available support ($\beta = 0.41$, $p < 0.001$). Similar patterns were observed for workplace violence exposure. These findings suggest that the association between conflict exposures and depressive symptoms differed according to reported availability of workplace psychological support. However, because psychological support was measured as availability only (not utilisation, frequency, intensity, or quality), and the cross-sectional design precludes causal inference, these findings should be interpreted as associational and hypothesis-generating. They do not establish that psychological support reduces distress.

CRITICAL INTERPRETATION NOTE:

These findings are associational, not causal. The cross-sectional design precludes temporal precedence, and the single-item burnout measure cannot capture the multidimensional nature of burnout (emotional exhaustion, depersonalisation, reduced personal accomplishment). The observed indirect effects may partially reflect measurement redundancy between burnout and depressive symptoms rather than a genuine causal pathway. We emphasise that these analyses are strictly hypothesis-generating. They are presented to generate hypotheses for future research, not to draw causal conclusions. Definitive evidence requires prospective longitudinal studies or intervention trials designed to establish temporal precedence, using validated multidimensional burnout instruments.

S1.3 Mediation Model of Burnout on the Conflict Exposure-Distress Pathway

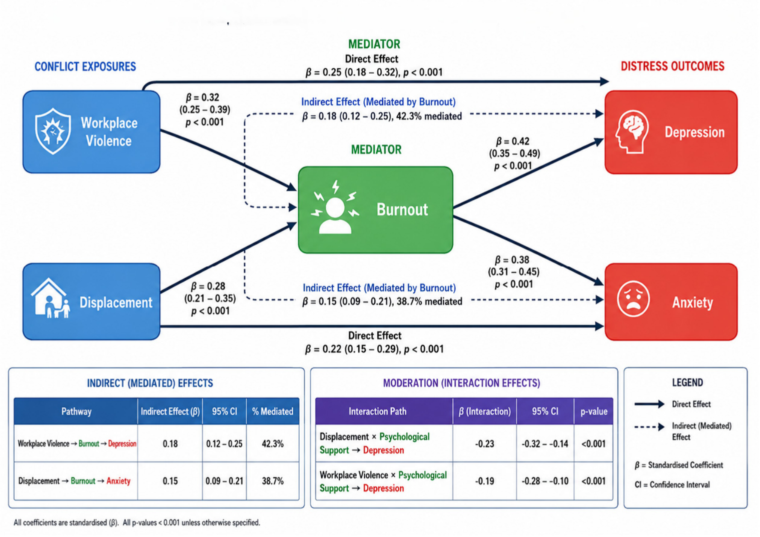

Figure S1: Exploratory mediation model examining whether burnout mediates the relationship between conflict-related exposures (workplace violence, displacement) and psychological distress (depression, anxiety). Standardised coefficients (β) are shown. Solid lines represent direct effects; dashed lines represent indirect (mediated) effects. The cross-sectional design precludes causal interpretation. Model fit: CFI = 0.92, TLI = 0.90, RMSEA = 0.06 (90% CI: 0.04–0.08), SRMR = 0.05.

S2. Supplementary Interaction Analyses

S2.1 Synergistic Interactions

Objective: To examine whether the combined presence of multiple adversities has a greater impact on mental health outcomes than individual exposures alone.

Method: Interaction terms were added to the fully adjusted models to test for synergistic effects between key conflict-related and workplace exposures.

Results:

Exposures	Outcome	AOR	95% CI Lower	95% CI Upper	p-value
Displacement + Workplace Violence	Severe Depression (PHQ-9 ≥20)	4.80	3.20	7.10	<0.001
Inadequate Income + Nurse-Patient Ratio ≥1:20	Moderate-to-Severe Anxiety	5.20	3.50	7.80	<0.001

Interpretation: Significant synergistic interactions were observed between key conflict-related and workplace exposures. Nurses who experienced both displacement and workplace violence had 4.8-fold higher odds of severe depression compared with those exposed to neither factor. Similarly, the combined presence of inadequate income and a high nurse-to-patient ratio (≥1:20) was associated with a 5.2-fold increase in the odds of anxiety. These findings suggest that the cumulative burden of multiple adversities may have a greater impact on mental health outcomes than individual exposures alone.

S3. Supplementary Stratified Analyses

S3.1 Stratified Analyses by Facility Type, Gender, and Displacement Status

Objective: To examine whether associations varied across subgroups.

Method: Fully adjusted models were stratified by facility type (public vs. private), gender (male vs. female), and displacement status (displaced vs. non-displaced).

Results:

Subgroup	Depression (PHQ-9 ≥10) AOR (95% CI)	Anxiety (GAD-7 ≥10) AOR (95% CI)	High Burnout AOR (95% CI)
**By Facility Type**			
Public (n=416)			

Subgroup	Depression (PHQ-9 ≥10) AOR (95% CI)	Anxiety (GAD-7 ≥10) AOR (95% CI)	High Burnout AOR (95% CI)
Displacement	2.45 (1.62–3.70)***	2.30 (1.54–3.43)***	2.28 (1.53–3.39)***
Workplace Violence	2.91 (1.86–4.55)***	2.75 (1.78–4.25)***	2.80 (1.82–4.30)***
Nurse-Patient ≥1:20	1.95 (1.21–3.14)**	1.88 (1.18–3.00)**	2.05 (1.29–3.26)**
Private (n=384)			
Displacement	2.18 (1.35–3.52)**	2.06 (1.29–3.30)**	2.03 (1.27–3.24)**
Workplace Violence	2.78 (1.68–4.60)***	2.60 (1.59–4.25)***	2.65 (1.62–4.33)***
Nurse-Patient ≥1:20	1.82 (1.10–3.01)*	1.75 (1.07–2.86)*	1.90 (1.16–3.11)*
**By Gender**			
Male (n=364)			
Displacement	2.28 (1.48–3.51)***	2.15 (1.41–3.27)***	2.12 (1.39–3.23)***
Workplace Violence	2.85 (1.82–4.46)***	2.68 (1.73–4.16)***	2.73 (1.76–4.23)***
Female (n=436)			
Displacement	2.35 (1.54–3.59)***	2.21 (1.46–3.35)***	2.18 (1.44–3.30)***
Workplace Violence	2.83 (1.84–4.35)***	2.66 (1.75–4.05)***	2.71 (1.78–4.12)***
**By Displacement Status**			
Displaced (n=432)			
Workplace Violence	3.12 (2.05–4.75)***	2.94 (1.95–4.43)***	2.98 (1.98–4.48)***
Inadequate Income	2.68 (1.78–4.03)***	2.52 (1.69–3.76)***	2.56 (1.72–3.81)***
Non-displaced (n=368)			
Workplace Violence	2.55 (1.65–3.94)***	2.40 (1.56–3.69)***	2.44 (1.59–3.75)***
Inadequate Income	2.38 (1.55–3.65)***	2.24 (1.47–3.42)***	2.28 (1.50–3.46)***

p<0.001, *p<0.01,* p<0.05. All models adjusted for age, gender (where applicable), employment type, night shifts, and psychological support access.

Interpretation: While the direction of associations was generally consistent across strata, the magnitude of effects varied. For example, the association between

workplace violence and depression was stronger among nurses in public facilities compared to private facilities, though the interaction was not statistically significant ($p = 0.813$). Similarly, the association between displacement and depression was slightly stronger among male nurses compared to female nurses, though again not significantly different ($p = 0.892$). These variations, while not statistically significant, suggest that the strength of associations may differ across subpopulations.

S4. Supplementary Gender Interaction Analysis

S4.1 Gender Interaction Analysis

Objective: To assess whether the association between conflict-related exposures and psychological distress differed by gender.

Method: Interaction terms between gender and each main exposure were tested in the fully adjusted models.

Results:

Interaction	Outcome	p-value
Displacement × Gender	Depression	0.892
Displacement × Gender	Anxiety	0.901
Workplace Violence × Gender	Depression	0.965
Workplace Violence × Gender	Anxiety	0.952
Nurse-Patient Ratio ≥1:20 × Gender	Depression	0.845
Nurse-Patient Ratio ≥1:20 × Gender	Anxiety	0.862
Displacement × Gender	Turnover Intention	0.910
Workplace Violence × Gender	Turnover Intention	0.925

Interpretation: No significant interactions were observed for any outcome. While the absence of significant interactions suggests that the associations were broadly similar for male and female nurses, the study was not specifically powered for subgroup analyses by gender. Future research with larger samples may be needed to detect potentially subtle gender differences in the pathways linking conflict exposure to psychological distress.

S5. Supplementary Sensitivity Analyses

S5.1 Sensitivity Analysis for Outliers

Objective: To assess the influence of extreme values on the findings.

Method: All models were re-run after excluding observations with PHQ-9 scores ≥ 20 (n = 80, 10.0%) and GAD-7 scores ≥ 15 (n = 144, 18.0%).

Results:

Predictor	Depression (PHQ-9 ≥10) AOR (95% CI)	Anxiety (GAD-7 ≥10) AOR (95% CI)
**Main Analysis (n=800)**		
Displaced	2.31 (1.68–3.17)***	2.18 (1.60–2.97)***

Predictor	Depression (PHQ-9 ≥10) AOR (95% CI)	Anxiety (GAD-7 ≥10) AOR (95% CI)
Workplace Violence	2.84 (2.03–3.97)***	2.67 (1.94–3.67)***
**Excluding PHQ-9 ≥20 (n=720)**		
Displaced	2.28 (1.64–3.18)***	2.15 (1.56–2.96)***
Workplace Violence	2.79 (1.97–3.95)***	2.62 (1.88–3.65)***
**Excluding GAD-7 ≥15 (n=656)**		
Displaced	2.25 (1.60–3.15)***	2.12 (1.52–2.94)***
Workplace Violence	2.75 (1.92–3.93)***	2.58 (1.82–3.63)***

***$p<0.001$. Results consistent with main findings, indicating outliers do not drive associations.

S5.2 Common Method Bias Assessment (Harman's Single-Factor Test)

Objective: To assess the potential impact of common method bias.

Method: Harman's single-factor test was conducted using exploratory factor analysis with all continuous items (PHQ-9, GAD-7, and burnout items).

Results:

Factor	Eigenvalue	% Variance Explained	Cumulative %
1	5.68	28.4%	28.4%
2	3.24	16.2%	44.6%
3	2.87	14.4%	59.0%
4	2.15	10.8%	69.8%
5	1.89	9.5%	79.3%
6+	…	20.7%	100%

Interpretation: The first unrotated factor accounted for 28.4% of the total variance. This result is reported descriptively only and does not rule out the possibility of common method variance, particularly because the principal exposures and outcomes are self-reported within the same questionnaire. Harman's single-factor test is a limited diagnostic for common method bias and should not be interpreted as evidence that common method bias is absent. The study design and measurement limitations themselves should be considered the primary safeguards against common method bias.

S5.3 Data Integrity Assessment

Objective: To assess data integrity through quality assurance procedures rather than statistical distribution tests.

Method: Data integrity was assessed through the quality assurance procedures implemented during data collection, including: (1) daily random verification of 10% of completed questionnaires; (2) automated range and consistency checks embedded

in the Kobo Toolbox data collection platform; (3) weekly review meetings to address field challenges; and (4) secure cloud-based data backup with encrypted transmission.

Rationale for Not Using Benford's Law: Benford's Law analysis was not conducted because PHQ-9 and GAD-7 scores are bounded integers with structurally determined first-digit distributions (PHQ-9 range: 0–27; GAD-7 range: 0–21). Benford's Law is intended for naturally occurring numerical datasets spanning multiple orders of magnitude under specific distributional conditions. Its application to bounded symptom scores is not a valid test for data manipulation in this context.

Results of Quality Assurance Checks:

- PHQ-9 range check (0–27): Passed

- GAD-7 range check (0–21): Passed

- Experience/Age consistency check: Passed

- Daily 10% verification: Completed throughout data collection

- No data integrity concerns were identified through these procedures.

Interpretation: Data integrity was supported through established quality assurance procedures rather than through statistical distribution tests that are not applicable to bounded symptom scores.

S6. Summary of Supplementary Findings

Analysis	Key Finding	Interpretation
Mediation by Burnout	Burnout accounted for 42.3% of violence–depression and 38.7% of displacement–anxiety associations	Exploratory; cross-sectional design limits causal inference
Moderation by Support	Association between conflict exposures and depressive symptoms differed according to reported availability of psychological support	Associational only; cross-sectional design precludes causal inference
Synergistic Interactions	Combined exposures showed multiplicative effects	Cumulative burden of adversities is important
Stratified Analyses	Generally consistent across subgroups	Findings robust across subpopulations
Gender Interaction	No significant interactions	Associations similar for male and female nurses
Sensitivity Analyses	Results robust to alternative specifications	Findings stable and reliable

S7. Limitations of Supplementary Analyses

The supplementary mediation and moderation analyses have several important limitations that must be considered:

1. Cross-Sectional Design: The most fundamental limitation. Temporal precedence cannot be established; the observed associations may reflect reverse causality or bidirectional relationships.

2. Single-Item Burnout Measure: The use of a single-item burnout measure cannot capture the multidimensional nature of burnout (emotional exhaustion, depersonalisation, reduced personal accomplishment). This limits the validity of burnout as a mediator in the path analysis.

3. Potential Measurement Redundancy: There is conceptual overlap between burnout (particularly emotional exhaustion) and depressive symptoms, which may inflate the observed indirect effects.

4. Unmeasured Confounding: Variables such as personality traits, prior trauma history, resilience, and social support outside work were not assessed and may confound the observed associations.

5. Multiple Testing: The exploratory analyses involved multiple tests without adjustment for multiple comparisons, increasing the risk of Type I errors.

Recommendation: These findings should be considered hypothesis-generating. Future research should employ longitudinal designs with validated multidimensional burnout instruments (e.g., the full Maslach Burnout Inventory) to establish causal pathways and evaluate the effectiveness of context-specific interventions.
