# Supplementary material for: Mental Health, Burnout, and Turnover Intention Among Nurses in Conflict-Affected Yemen: A Multicenter Study Across Eight Accessible Governorates
Source: Nurs Rep. 2026 Oct 7;16(10):353. doi: 10.3390/nursrep16100353 (PMC13708594; doi:10.3390/nursrep16100353)
Supplement: Supplementary file 1 [file nursrep-16-00353-s001.zip › File S6 Data Dictionary Codebook.pdf]

FILE S6: Data Dictionary / Codebook

VARIABLE DICTIONARY

Variable Name	Description	Type	Values/Coding	Missing Codes
Nurse_ID	Unique participant identifier	Integer	1-800	None
Age	Age in years	Continuous	22-58	-99
Gender	Biological sex	Categorical	0 = Male 1 = Female	-99
Marital_Status	Marital status	Categorical	1 = Single 2 = Married 3 = Divorced 4 = Widowed	-99
Education_Level	Highest nursing qualification	Ordinal	1 = Diploma 2 = Bachelor 3 = Master	-99
Years_of_Experience	Years working as nurse	Continuous	1-30	-99
Employment_Type	Type of employment	Categorical	1 = Permanent 2 = Contract 3 = Volunteer	-99
Facility_Type	Type of health facility	Categorical	1 = Public 2 = Private 3 = NGO	-99
Working_Hours_Per_Shift	Hours per shift	Continuous	8, 10, 12	-99
Night_Shifts_Per_Month	Number of night shifts monthly	Continuous	0-12	-99
Nurse_Patient_Ratio	Typical patient load	Ordinal	1 = 1:10 2 = 1:15 3 = 1:20 4 = 1:25	-99
Exposure_to_Workplace_Violence	Violence exposure in past 6 months	Binary	0 = No 1 = Yes	-99
Violence_Type	Type of violence (if exposed)	Categorical multiple	1 = Physical 2 = Verbal 3 = Psychological	-99
Displacement_Status	Displacement due to conflict	Binary	0 = Non-displaced 1 = Displaced	-99
Monthly_Income_Adequacy	Self-rated income adequacy	Binary	0 = Adequate 1 = Inadequate	-99
Access_to_Psychological_Support	Availability of mental health services	Binary	0 = No access 1 = Access	-99
PHQ9_Score	Total depression score	Continuous	0-27	-99
Depression_Severity	PHQ-9 severity category	Ordinal	1 = Minimal (0-4) 2 = Mild (5-9) 3 = Moderate (10-14) 4 = Moderately	-99

			severe (15-19) 5 = Severe (20-27)	
GAD7_Score	Total anxiety score	Continuous	0-21	-99
Anxiety_Severity	GAD-7 severity category	Ordinal	1 = Minimal (0-4) 2 = Mild (5-9) 3 = Moderate (10-14) 4 = Severe (15-21)	-99
Burnout_Level	Single-item burnout rating	Ordinal	1 = Low 2 = Moderate 3 = High	-99
Intention_to_Leave	Turnover intention	Binary	0 = No 1 = Yes	-99
Facility_Code	Facility identifier	Categorical	F01-F24	None
Governorate	Location	Categorical	1-8 (coded)	-99
Collection_Date	Date of data collection	Date	DD/MM/YYYY	-99

SCORE CALCULATION METHODS

PHQ-9 SCORING:

1. Item scores: 0-3 for each of 9 items

2. Total score: Sum of items 1-9 (range: 0-27)

3. Severity categories:

· 0-4: Minimal depression
· 5-9: Mild depression
· 10-14: Moderate depression
· 15-19: Moderately severe depression
· 20-27: Severe depression

GAD-7 SCORING:

1. Item scores: 0-3 for each of 7 items

2. Total score: Sum of items 1-7 (range: 0-21)

3. Severity categories:

· 0-4: Minimal anxiety
· 5-9: Mild anxiety
· 10-14: Moderate anxiety
· 15-21: Severe anxiety

BURNOUT SCORING:

Single-item measure: "How would you rate your level of burnout?"

· 1 = Low
· 2 = Moderate
· 3 = High

DERIVED VARIABLES

Variable	Creation Rule	Purpose
depression_binary	PHQ9_Score ≥ 10 = 1, else 0	Binary outcome for logistic regression
anxiety_binary	GAD7_Score ≥ 10 = 1, else 0	Binary outcome for logistic regression
turnover_binary	Intention_to_Leave = "Yes" = 1, else 0	Binary outcome
high_burnout	Burnout_Level = "High" = 1, else 0	Binary predictor
violence_exposed	Exposure_to_Workplace_Violence = "Yes" = 1, else 0	Binary predictor
displaced	Displacement_Status = "Displaced" = 1, else 0	Binary predictor
income_inadequate	Monthly_Income_Adequacy = "Inadequate" = 1, else 0	Binary predictor

MISSING DATA HANDLING

Missing Value Codes:

· -99: Missing/Not answered
· -88: Not applicable
· -77: Refused to answer

Imputation Rules:

1. Demographic variables: No imputation
2. Outcome variables: Complete-case analysis
3. Predictor variables: Multiple imputation (m=20) if missing <10%

DATA QUALITY CHECKS

Range Checks:

· Age: 18-65 (outside range flagged)
· PHQ-9: 0-27 (outside range invalid)
· GAD-7: 0-21 (outside range invalid)

Consistency Checks:

· Years of experience ≤ Age - 18
· Night shifts ≤ 31 (days in month)
· Working hours: 4-16 hours per shift

Skip Patterns:

· If Violence_Type present, Exposure_to_Workplace_Violence must = 1
· If depression_binary = 1, PHQ9_Score must ≥ 10

VARIABLE TRANSFORMATIONS

For Analysis:

```
# Standardization (for regression)
data$Age_z <- scale(data$Age)
data$Experience_z <- scale(data$Years_of_Experience)

# Dummy coding
data$Gender_Male <- ifelse(data$Gender == 0, 1, 0)
data$Facility_Public <- ifelse(data$Facility_Type == 1, 1, 0)

# Interaction terms
data$Displaced_Violence <- data$displaced * data$violence_exposed
```
