# Supplementary material for: Mental Health, Burnout, and Turnover Intention Among Nurses in Conflict-Affected Yemen: A Multicenter Study Across Eight Accessible Governorates
Source: Nurs Rep. 2026 Oct 7;16(10):353. doi: 10.3390/nursrep16100353 (PMC13708594; doi:10.3390/nursrep16100353)
Supplement: Supplementary file 1 [file nursrep-16-00353-s001.zip › File S8 STATISTICAL ANALYSIS CODE (R).pdf]

```
# ==========================================================================
# FILE S8 – STATISTICAL ANALYSIS CODE (R)
# ==========================================================================
# Study Title: Mental Health, Burnout and Turnover Intention among Nurses
#              in Conflict-Affected Yemen: A Multicentre Study Across Eight
#              Accessible Governorates
# Corresponding Author: Naif Taleb Ali
# IRB Reference Number: YEM-IRB-2025-094
# ==========================================================================


# ==========================================================================
# 1. LIBRARIES
# ==========================================================================

library(tidyverse)
library(lme4)
library(ordinal)        # For clmm (cumulative link mixed models)
library(mediation)
library(mice)
library(sandwich)
library(lmtest)
library(sjPlot)
library(gtsummary)
library(psych)
library(lavaan)         # For SEM
library(performance)    # For model diagnostics
library(see)            # For visualisation
library(MASS)           # For polr
library(pROC)           # For AUC
library(caret)          # For cross-validation
library(survey)         # For sampling weights
library(interactions)   # For simple slopes
library(DHARMa)         # For residual diagnostics
library(ResourceSelection) # For Hosmer-Lemeshow
library(car)            # For VIF
library(semPlot)        # For SEM diagram (Figure 4)
library(glue)           # For string formatting
library(broom.mixed)    # For tidy model outputs


# ==========================================================================
# 2. SETUP
# ==========================================================================

set.seed(20250731)      # For reproducibility
options(scipen = 999)


# ==========================================================================
# 3. DATA PREPARATION
# ==========================================================================

# 3.1 Load data
data <- read_csv("File_S7_Deidentified_Dataset.csv")

# 3.2 Check structure
str(data)
names(data)

# 3.3 Recode variables
data_clean <- data %>%
  mutate(
    # Binary outcomes
    depression_binary = ifelse(PHQ9_Score >= 10, 1, 0),
    anxiety_binary    = ifelse(GAD7_Score >= 10, 1, 0),
    turnover_binary   = ifelse(Intention_to_Leave == "Yes", 1, 0),
```

```r
    # Categorical to factor
    Gender                   = factor(Gender, levels = c("Male", "Female")),
    Marital_Status           = factor(Marital_Status),
    Education_Level          = factor(Education_Level,
                                      levels = c("Diploma", "Bachelor", "Master")),
    Employment_Type          = factor(Employment_Type),
    Facility_Type            = factor(Facility_Type),
    Displacement_Status      = factor(Displacement_Status),
    Monthly_Income_Adequacy  = factor(Monthly_Income_Adequacy),
    Exposure_to_Workplace_Violence = factor(Exposure_to_Workplace_Violence),
    Access_to_Psychological_Support = factor(Access_to_Psychological_Support),

    # Burnout ordinal factor (prespecified 3-category coding)
    Burnout_Level = factor(Burnout_Level, levels = c("Low", "Moderate", "High"),
                           ordered = TRUE),

    # Nurse-patient ratio factor (4 categories for regression)
    Nurse_Patient_Ratio = factor(Nurse_Patient_Ratio,
                                 levels = c("1:10", "1:15", "1:20", "1:25")),

    # Facility code for clustering
    Facility_Code = factor(Facility_Code)
  )


# ==============================================================================
# 4. DESCRIPTIVE TABLES
# ==============================================================================

# 4.1 Table 1: Participant characteristics
table1 <- data_clean %>%
  select(Age, Gender, Marital_Status, Education_Level, Years_of_Experience,
         Employment_Type, Facility_Type, Working_Hours_Per_Shift,
         Night_Shifts_Per_Month, Nurse_Patient_Ratio, Displacement_Status,
         Monthly_Income_Adequacy, Exposure_to_Workplace_Violence,
         Access_to_Psychological_Support) %>%
  tbl_summary(
    statistic = list(
      all_continuous()  ~ "{mean} ({sd})",
      all_categorical() ~ "{n} ({p}%)"
    ),
    digits = all_continuous() ~ 1
  ) %>%
  bold_labels()

# 4.2 Table 2: Prevalence of outcomes
table2 <- data_clean %>%
  select(PHQ9_Score, GAD7_Score, Burnout_Level, turnover_binary) %>%
  tbl_summary(
    statistic = list(
      all_continuous()  ~ "{mean} ({sd})",
      all_categorical() ~ "{n} ({p}%)"
    ),
    label = list(
      PHQ9_Score      ~ "Depression Score (PHQ-9)",
      GAD7_Score      ~ "Anxiety Score (GAD-7)",
      Burnout_Level   ~ "Burnout Level",
      turnover_binary ~ "Intention to Leave"
    )
  ) %>%
  bold_labels()

# 4.3 Data distribution checks
distribution_checks <- data_clean %>%
  summarize(
    PHQ9_mean = mean(PHQ9_Score, na.rm = TRUE),
    PHQ9_sd   = sd(PHQ9_Score,   na.rm = TRUE),
    PHQ9_min  = min(PHQ9_Score,  na.rm = TRUE),
    PHQ9_max  = max(PHQ9_Score,  na.rm = TRUE),
```

```
    GAD7_mean = mean(GAD7_Score, na.rm = TRUE),
    GAD7_sd   = sd(GAD7_Score,   na.rm = TRUE),
    GAD7_min  = min(GAD7_Score,  na.rm = TRUE),
    GAD7_max  = max(GAD7_Score,  na.rm = TRUE),
    correlation = cor(PHQ9_Score, GAD7_Score, use = "complete.obs")
  )
print(distribution_checks)

# 4.4 Shapiro-Wilk tests for normality
shapiro_phq9 <- shapiro.test(data_clean$PHQ9_Score)
shapiro_gad7 <- shapiro.test(data_clean$GAD7_Score)
print(shapiro_phq9)
print(shapiro_gad7)


# ============================================================================
# 5. MULTIVARIABLE MODELS
# ============================================================================

# 5.1 Model 1: Depression (mixed-effects logistic)
model_depression <- glmer(
  depression_binary ~ Age + Gender + Displacement_Status +
    Monthly_Income_Adequacy + Exposure_to_Workplace_Violence +
    Nurse_Patient_Ratio + Night_Shifts_Per_Month +
    Access_to_Psychological_Support + Employment_Type +
    (1 | Facility_Code),
  data = data_clean,
  family = binomial,
  control = glmerControl(optimizer = "bobyqa")
)
summary(model_depression)

# 5.2 Model 2: Anxiety (mixed-effects logistic)
model_anxiety <- glmer(
  anxiety_binary ~ Age + Gender + Displacement_Status +
    Monthly_Income_Adequacy + Exposure_to_Workplace_Violence +
    Nurse_Patient_Ratio + Night_Shifts_Per_Month +
    Access_to_Psychological_Support + Employment_Type +
    (1 | Facility_Code),
  data = data_clean,
  family = binomial,
  control = glmerControl(optimizer = "bobyqa")
)
summary(model_anxiety)

# 5.3a Model 3a: Turnover (logistic) — WITHOUT mental health variables
model_turnover_model1 <- glmer(
  turnover_binary ~ Age + Gender + Displacement_Status +
    Monthly_Income_Adequacy + Exposure_to_Workplace_Violence +
    Nurse_Patient_Ratio + Night_Shifts_Per_Month +
    Access_to_Psychological_Support + Employment_Type +
    (1 | Facility_Code),
  data = data_clean,
  family = binomial,
  control = glmerControl(optimizer = "bobyqa")
)
summary(model_turnover_model1)

# 5.3b Model 3b: Turnover (logistic) — WITH mental health variables
model_turnover_model2 <- glmer(
  turnover_binary ~ depression_binary + anxiety_binary + Burnout_Level +
    Displacement_Status + Monthly_Income_Adequacy +
    Exposure_to_Workplace_Violence + Nurse_Patient_Ratio +
    Access_to_Psychological_Support + Employment_Type +
    (1 | Facility_Code),
  data = data_clean,
  family = binomial,
  control = glmerControl(optimizer = "bobyqa")
)
```

```
summary(model_turnover_model2)

# 5.4 Model 4: Linear regression for continuous scores
model_phq_linear <- lmer(
  PHQ9_Score ~ Age + Gender + Displacement_Status +
    Monthly_Income_Adequacy + Exposure_to_Workplace_Violence +
    Nurse_Patient_Ratio + Night_Shifts_Per_Month +
    Access_to_Psychological_Support + Employment_Type +
    (1 | Facility_Code),
  data = data_clean
)
summary(model_phq_linear)

model_gad_linear <- lmer(
  GAD7_Score ~ Age + Gender + Displacement_Status +
    Monthly_Income_Adequacy + Exposure_to_Workplace_Violence +
    Nurse_Patient_Ratio + Night_Shifts_Per_Month +
    Access_to_Psychological_Support + Employment_Type +
    (1 | Facility_Code),
  data = data_clean
)
summary(model_gad_linear)

# 5.5 Model 5: Ordinal regression for burnout
model_burnout_ordinal <- clmm(
  Burnout_Level ~ Age + Gender + Displacement_Status +
    Monthly_Income_Adequacy + Exposure_to_Workplace_Violence +
    Nurse_Patient_Ratio + Night_Shifts_Per_Month +
    Access_to_Psychological_Support + Employment_Type +
    (1 | Facility_Code),
  data = data_clean,
  Hess = TRUE
)
summary(model_burnout_ordinal)


# ============================================================================
# 6. ICC CALCULATION (NULL MODELS)
# ============================================================================

# 6.1 Null model for depression
null_depression <- glmer(
  depression_binary ~ 1 + (1 | Facility_Code),
  data = data_clean,
  family = binomial,
  control = glmerControl(optimizer = "bobyqa")
)

# 6.2 Null model for anxiety
null_anxiety <- glmer(
  anxiety_binary ~ 1 + (1 | Facility_Code),
  data = data_clean,
  family = binomial,
  control = glmerControl(optimizer = "bobyqa")
)

# 6.3 Null model for burnout
null_burnout <- clmm(
  Burnout_Level ~ 1 + (1 | Facility_Code),
  data = data_clean
)

# 6.4 Null model for turnover
null_turnover <- glmer(
  turnover_binary ~ 1 + (1 | Facility_Code),
  data = data_clean,
  family = binomial,
  control = glmerControl(optimizer = "bobyqa")
)
```

```r
# 6.5 Extract ICC values
icc_depression <- performance::icc(null_depression)
icc_anxiety    <- performance::icc(null_anxiety)
icc_turnover   <- performance::icc(null_turnover)

# For clmm, compute ICC manually (latent-scale approach)
var_facility_burnout <- as.numeric(VarCorr(null_burnout)$Facility_Code[1, 1])
icc_burnout <- var_facility_burnout / (var_facility_burnout + (pi^2 / 3))

print(icc_depression)
print(icc_anxiety)
print(icc_turnover)
cat("Burnout ICC (manual):", round(icc_burnout, 3), "\n")


# ============================================================================
# 7. MEDIATION ANALYSIS (EXPLORATORY – HYPOTHESIS-GENERATING ONLY)
# ============================================================================
# NOTE: These analyses are strictly exploratory and associational.
# They must NOT be interpreted as evidence of causal mediation.
# Cross-sectional design precludes causal inference.

# 7.1 Mediation: Workplace Violence -> Burnout -> Depression
med_model1 <- lm(
  as.numeric(Burnout_Level) ~ Exposure_to_Workplace_Violence +
    Age + Gender + Displacement_Status + Monthly_Income_Adequacy,
  data = data_clean
)

med_model2 <- glm(
  depression_binary ~ as.numeric(Burnout_Level) +
    Exposure_to_Workplace_Violence +
    Age + Gender + Displacement_Status + Monthly_Income_Adequacy,
  data = data_clean,
  family = binomial
)

med_results <- mediate(
  med_model1,
  med_model2,
  treat    = "Exposure_to_Workplace_Violence",
  mediator = "as.numeric(Burnout_Level)",
  robustSE = TRUE,
  sims     = 5000
)
summary(med_results)

# 7.2 Mediation: Displacement -> Burnout -> Anxiety
med_model3 <- lm(
  as.numeric(Burnout_Level) ~ Displacement_Status +
    Age + Gender + Exposure_to_Workplace_Violence + Monthly_Income_Adequacy,
  data = data_clean
)

med_model4 <- glm(
  anxiety_binary ~ as.numeric(Burnout_Level) +
    Displacement_Status +
    Age + Gender + Exposure_to_Workplace_Violence + Monthly_Income_Adequacy,
  data = data_clean,
  family = binomial
)

med_results2 <- mediate(
  med_model3,
  med_model4,
  treat    = "Displacement_Status",
  mediator = "as.numeric(Burnout_Level)",
  robustSE = TRUE,
```

```r
  sims     = 5000
)
summary(med_results2)


# ==============================================================================
# 8. MODERATION ANALYSIS (EXPLORATORY)
# ==============================================================================

# 8.1 Moderation: Psychological Support x Displacement -> Depression
moderation_model <- glm(
  depression_binary ~ Displacement_Status * Access_to_Psychological_Support +
    Age + Gender + Monthly_Income_Adequacy +
    Exposure_to_Workplace_Violence + Nurse_Patient_Ratio,
  data = data_clean,
  family = binomial
)
summary(moderation_model)

# 8.2 Simple slopes analysis
sim_slopes(
  moderation_model,
  pred = Displacement_Status,
  modx = Access_to_Psychological_Support
)

# 8.3 Interaction plot
interact_plot(
  moderation_model,
  pred    = Displacement_Status,
  modx    = Access_to_Psychological_Support,
  interval = TRUE,
  int.width = 0.95,
  title = "Moderation Effect: Psychological Support on Displacement-Depression Association"
)


# ==============================================================================
# 9. SENSITIVITY ANALYSES
# ==============================================================================

# 9.1 Multiple imputation for missing data
if (any(is.na(data_clean))) {
  pred_matrix <- quickpred(data_clean)

  imputed_data <- mice(
    data_clean,
    m = 20,
    maxit = 10,
    method = "pmm",
    seed = 20250731
  )

  pool_models <- with(imputed_data, glmer(
    depression_binary ~ Age + Gender + Displacement_Status +
      Monthly_Income_Adequacy + (1 | Facility_Code),
    family = binomial
  ))

  pooled_results <- pool(pool_models)
  summary(pooled_results)
}

# 9.2 Alternative cut-offs — Depression: PHQ-9 >= 5
data_clean <- data_clean %>%
  mutate(depression_mild = ifelse(PHQ9_Score >= 5, 1, 0))

model_depression_mild <- glmer(
  depression_mild ~ Age + Gender + Displacement_Status +
```

```
    Monthly_Income_Adequacy + Exposure_to_Workplace_Violence +
    (1 | Facility_Code),
  data = data_clean,
  family = binomial,
  control = glmerControl(optimizer = "bobyqa")
)
summary(model_depression_mild)

# 9.3 Alternative cut-offs — Anxiety: GAD-7 >= 8
data_clean <- data_clean %>%
  mutate(anxiety_moderate = ifelse(GAD7_Score >= 8, 1, 0))

model_anxiety_moderate <- glmer(
  anxiety_moderate ~ Age + Gender + Displacement_Status +
    Monthly_Income_Adequacy + Exposure_to_Workplace_Violence +
    (1 | Facility_Code),
  data = data_clean,
  family = binomial,
  control = glmerControl(optimizer = "bobyqa")
)
summary(model_anxiety_moderate)

# 9.4 Subgroup analyses — By facility type
model_public <- glmer(
  depression_binary ~ Age + Gender + Displacement_Status +
    Monthly_Income_Adequacy + Exposure_to_Workplace_Violence +
    (1 | Facility_Code),
  data = filter(data_clean, Facility_Type == "Public"),
  family = binomial,
  control = glmerControl(optimizer = "bobyqa")
)

model_private <- glmer(
  depression_binary ~ Age + Gender + Displacement_Status +
    Monthly_Income_Adequacy + Exposure_to_Workplace_Violence +
    (1 | Facility_Code),
  data = filter(data_clean, Facility_Type == "Private"),
  family = binomial,
  control = glmerControl(optimizer = "bobyqa")
)
summary(model_public)
summary(model_private)

# 9.5 Outlier sensitivity — Excluding PHQ-9 >= 20
data_no_outliers <- data_clean %>% filter(PHQ9_Score < 20)

model_no_outliers <- glmer(
  depression_binary ~ Age + Gender + Displacement_Status +
    Monthly_Income_Adequacy + Exposure_to_Workplace_Violence +
    (1 | Facility_Code),
  data = data_no_outliers,
  family = binomial,
  control = glmerControl(optimizer = "bobyqa")
)
summary(model_no_outliers)

# 9.6 Data Integrity Assessment
# Note: Benford's Law analysis was NOT conducted because PHQ-9 and GAD-7 scores
# are bounded integers with structurally determined first-digit distributions
# (PHQ-9 range: 0-27; GAD-7 range: 0-21). Benford's Law is not a valid test
# for data manipulation when the numerical range is constrained.
#
# Data integrity was instead assessed through quality assurance procedures:
#   - Daily random verification of 10% of completed questionnaires
#   - Automated range and consistency checks (Kobo Toolbox)
#   - Weekly review meetings

range_check_phq9 <- all(data_clean$PHQ9_Score >= 0 & data_clean$PHQ9_Score <= 27,
                        na.rm = TRUE)
```

```
range_check_gad7 <- all(data_clean$GAD7_Score >= 0 & data_clean$GAD7_Score <= 21,
                        na.rm = TRUE)
consistency_check_age <- all(data_clean$Years_of_Experience <= (data_clean$Age - 18),
                             na.rm = TRUE)

cat("PHQ-9 range check passed:", range_check_phq9, "\n")
cat("GAD-7 range check passed:", range_check_gad7, "\n")
cat("Experience/Age consistency check passed:", consistency_check_age, "\n")

# 9.7 Common Method Bias (Harman's Single-Factor Test)
# Using ALL continuous items (PHQ-9 items, GAD-7 items, burnout items)
harman_data <- data_clean %>%
  select(starts_with("PHQ9_Item"), starts_with("GAD7_Item"),
         starts_with("Burnout_Item"))

harman_fa <- psych::fa(harman_data, nfactors = 1, rotate = "none", fm = "pa")
harman_variance <- harman_fa$Vaccounted[2, 1] * 100
cat("First unrotated factor variance explained:",
    round(harman_variance, 1), "%\n")
# Note: This result is reported descriptively only and does not rule out
# common method variance.


# ============================================================================
# 9.8 SAMPLING-WEIGHTED ANALYSES
# ============================================================================
# The sampling design allocated approximately 100 nurses to each of the eight
# governorates, despite substantial differences in governorate nursing-workforce
# size. Therefore, selection probabilities differed across governorates, and
# unweighted estimates cannot be interpreted as population prevalence estimates
# for nurses across the accessible sampling frame.

# --- 9.8.1 Define governorate-level population sizes of registered nurses ---
# NOTE: Replace the values below with the ACTUAL numbers obtained from the
# Ministry of Public Health and Population (MoPHP) and Health Cluster Yemen.
# The values here are illustrative placeholders.

governorate_pop <- tibble(
  Governorate = c("Aden", "Lahj", "Abyan", "Shabwah",
                  "Hadramawt", "Al-Mahra", "Socotra", "Taiz"),
  N_nurses    = c(5200, 1450, 1180, 760,
                  2650, 380, 140, 3400)  # <-- REPLACE with actual MoPHP figures
)

# --- 9.8.2 Merge population sizes and compute sampling weights ---
data_clean <- data_clean %>%
  left_join(governorate_pop, by = "Governorate") %>%
  group_by(Governorate) %>%
  mutate(
    n_selected   = n(),                        # nurses sampled per governorate
    p_selection  = n_selected / N_nurses,      # selection probability
    weight       = 1 / p_selection             # inverse-probability weight
  ) %>%
  ungroup()

# --- 9.8.3 Define the survey design ---
survey_design <- svydesign(
  ids     = ~Facility_Code,   # cluster at facility level
  weights = ~weight,          # inverse-probability weights
  data    = data_clean,
  nest    = TRUE
)

# --- 9.8.4 Weighted prevalence estimates for principal outcomes ---
weighted_prev_depression <- svymean(~depression_binary, survey_design,
                                    na.rm = TRUE)
weighted_prev_anxiety    <- svymean(~anxiety_binary,    survey_design,
                                    na.rm = TRUE)
weighted_prev_turnover   <- svymean(~turnover_binary,   survey_design,
```

```
                                    na.rm = TRUE)
weighted_prev_burnout    <- svymean(~I(Burnout_Level == "High"),
                                    survey_design, na.rm = TRUE)

cat("\n=== SAMPLING-WEIGHTED PREVALENCE ESTIMATES ===\n")
cat("Depression (PHQ-9 >= 10): ", round(coef(weighted_prev_depression)[1]*100, 1),
    "% (95% CI: ", round(confint(weighted_prev_depression)[1]*100, 1), "-",
    round(confint(weighted_prev_depression)[2]*100, 1), "%)\n")
cat("Anxiety (GAD-7 >= 10):    ", round(coef(weighted_prev_anxiety)[1]*100, 1),
    "% (95% CI: ", round(confint(weighted_prev_anxiety)[1]*100, 1), "-",
    round(confint(weighted_prev_anxiety)[2]*100, 1), "%)\n")
cat("High Burnout:             ", round(coef(weighted_prev_burnout)[1]*100, 1),
    "% (95% CI: ", round(confint(weighted_prev_burnout)[1]*100, 1), "-",
    round(confint(weighted_prev_burnout)[2]*100, 1), "%)\n")
cat("Turnover Intention:       ", round(coef(weighted_prev_turnover)[1]*100, 1),
    "% (95% CI: ", round(confint(weighted_prev_turnover)[1]*100, 1), "-",
    round(confint(weighted_prev_turnover)[2]*100, 1), "%)\n")

# --- 9.8.5 Table S12: Weighted vs. Unweighted Prevalence Estimates ---
unweighted_prev <- data_clean %>%
  summarize(
    depression = mean(depression_binary, na.rm = TRUE) * 100,
    anxiety    = mean(anxiety_binary,    na.rm = TRUE) * 100,
    burnout    = mean(Burnout_Level == "High", na.rm = TRUE) * 100,
    turnover   = mean(turnover_binary,   na.rm = TRUE) * 100
  )

table_S12 <- tibble(
  Outcome         = c("Depression (PHQ-9 >= 10)", "Anxiety (GAD-7 >= 10)",
                      "High Burnout", "Turnover Intention"),
  Unweighted_Prev  = c(unweighted_prev$depression, unweighted_prev$anxiety,
                      unweighted_prev$burnout,   unweighted_prev$turnover),
  Unweighted_LCL   = c(NA, NA, NA, NA),  # Fill with exact binomial CIs
  Unweighted_UCL   = c(NA, NA, NA, NA),
  Weighted_Prev    = c(coef(weighted_prev_depression)[1]*100,
                      coef(weighted_prev_anxiety)[1]*100,
                      coef(weighted_prev_burnout)[1]*100,
                      coef(weighted_prev_turnover)[1]*100),
  Weighted_LCL     = c(confint(weighted_prev_depression)[1]*100,
                      confint(weighted_prev_anxiety)[1]*100,
                      confint(weighted_prev_burnout)[1]*100,
                      confint(weighted_prev_turnover)[1]*100),
  Weighted_UCL     = c(confint(weighted_prev_depression)[2]*100,
                      confint(weighted_prev_anxiety)[2]*100,
                      confint(weighted_prev_burnout)[2]*100,
                      confint(weighted_prev_turnover)[2]*100)
)

print(table_S12)
# Save for File S4
write_csv(table_S12, "results/table_S12_weighted_prevalence.csv")

# --- 9.8.6 Weighted logistic regression for depression ---
model_depression_weighted <- svyglm(
  depression_binary ~ Displacement_Status + Monthly_Income_Adequacy +
    Exposure_to_Workplace_Violence + Nurse_Patient_Ratio +
    Night_Shifts_Per_Month + Access_to_Psychological_Support +
    Employment_Type + Age + Gender,
  design = survey_design,
  family = quasibinomial()
)
summary(model_depression_weighted)

# --- 9.8.7 Weighted logistic regression for anxiety ---
model_anxiety_weighted <- svyglm(
  anxiety_binary ~ Displacement_Status + Monthly_Income_Adequacy +
    Exposure_to_Workplace_Violence + Nurse_Patient_Ratio +
    Night_Shifts_Per_Month + Access_to_Psychological_Support +
    Employment_Type + Age + Gender,
```

```r
  design = survey_design,
  family = quasibinomial()
)
summary(model_anxiety_weighted)

# --- 9.8.8 Weighted logistic regression for turnover ---
model_turnover_weighted <- svyglm(
  turnover_binary ~ Displacement_Status + Monthly_Income_Adequacy +
    Exposure_to_Workplace_Violence + Nurse_Patient_Ratio +
    Access_to_Psychological_Support + Employment_Type + Age + Gender,
  design = survey_design,
  family = quasibinomial()
)
summary(model_turnover_weighted)

# --- 9.8.9 Comparison table: Weighted vs. Unweighted estimates ---
comparison_table <- data.frame(
  Predictor = c("Displaced", "Workplace Violence", "Inadequate Income",
                "Nurse-Patient Ratio >= 1:20", "No Psychological Support"),
  Unweighted_AOR = c(2.31, 2.84, 2.53, 1.68, 1.82),
  Weighted_AOR   = c(exp(coef(model_depression_weighted))[2],
                     exp(coef(model_depression_weighted))[3],
                     exp(coef(model_depression_weighted))[4],
                     exp(coef(model_depression_weighted))[6],
                     exp(coef(model_depression_weighted))[7])
)
print(comparison_table)
write_csv(comparison_table, "results/table_S12_comparison.csv")


# ============================================================================
# 10. RISK RATIOS (POISSON REGRESSION WITH ROBUST VARIANCE)
# ============================================================================

# 10.1 Risk ratios for main exposures — Depression
rr_depression <- glm(
  depression_binary ~ Displacement_Status +
    Exposure_to_Workplace_Violence + Monthly_Income_Adequacy +
    Age + Gender + Night_Shifts_Per_Month + Nurse_Patient_Ratio +
    Access_to_Psychological_Support + Employment_Type,
  data = data_clean,
  family = poisson(link = "log")
)

rr_robust <- coeftest(rr_depression, vcov = vcovHC(rr_depression, type = "HC1"))
print(rr_robust)

# 10.2 Risk ratios for main exposures — Anxiety
rr_anxiety <- glm(
  anxiety_binary ~ Displacement_Status +
    Exposure_to_Workplace_Violence + Monthly_Income_Adequacy +
    Age + Gender + Night_Shifts_Per_Month + Nurse_Patient_Ratio +
    Access_to_Psychological_Support + Employment_Type,
  data = data_clean,
  family = poisson(link = "log")
)
rr_robust_anxiety <- coeftest(rr_anxiety, vcov = vcovHC(rr_anxiety, type = "HC1"))
print(rr_robust_anxiety)


# ============================================================================
# 11. MODEL DIAGNOSTICS
# ============================================================================

# 11.1 Multicollinearity (VIF)
vif_check <- car::vif(model_depression)
print(vif_check)

# 11.2 Model fit — Hosmer-Lemeshow
```

```
# NOTE: Hosmer-Lemeshow is not appropriate for GLMMs; use calibration instead.
# Provided here for reference only.
hoslem_test <- hoslem.test(
  fitted(model_depression),
  data_clean$depression_binary,
  g = 10
)
print(hoslem_test)

# 11.3 Residual diagnostics
residuals_check <- simulateResiduals(model_depression)
plot(residuals_check)

# 11.4 Influence diagnostics — Cook's distance
influence_glmm <- influence(model_depression, obs = TRUE)
cooks_distance <- cooks.distance(influence_glmm)
summary(cooks_distance)

# 11.5 DFBETAS
dfbetas_values <- dfbetas(influence_glmm)
summary(dfbetas_values)


# ============================================================================
# 12. MODEL PERFORMANCE (AUC, BRIER SCORES, 10-FOLD CV)
# ============================================================================

# 12.1 AUC for Depression Model (Apparent)
pred_depression <- predict(model_depression, type = "response")
roc_depression <- roc(data_clean$depression_binary, pred_depression)
auc_depression_apparent <- auc(roc_depression)
ci_auc_depression <- ci.auc(roc_depression)
cat("Depression Model - Apparent AUC:", round(auc_depression_apparent, 3),
    "95% CI:", round(ci_auc_depression[1], 3), "-",
    round(ci_auc_depression[3], 3), "\n")

# 12.2 AUC for Anxiety Model (Apparent)
pred_anxiety <- predict(model_anxiety, type = "response")
roc_anxiety <- roc(data_clean$anxiety_binary, pred_anxiety)
auc_anxiety_apparent <- auc(roc_anxiety)
ci_auc_anxiety <- ci.auc(roc_anxiety)
cat("Anxiety Model - Apparent AUC:", round(auc_anxiety_apparent, 3),
    "95% CI:", round(ci_auc_anxiety[1], 3), "-",
    round(ci_auc_anxiety[3], 3), "\n")

# 12.3 AUC for Turnover Model (Apparent)
pred_turnover <- predict(model_turnover_model1, type = "response")
roc_turnover <- roc(data_clean$turnover_binary, pred_turnover)
auc_turnover_apparent <- auc(roc_turnover)
ci_auc_turnover <- ci.auc(roc_turnover)
cat("Turnover Model - Apparent AUC:", round(auc_turnover_apparent, 3),
    "95% CI:", round(ci_auc_turnover[1], 3), "-",
    round(ci_auc_turnover[3], 3), "\n")

# 12.4 Brier Scores (Apparent)
brier_depression <- mean((pred_depression - data_clean$depression_binary)^2,
                         na.rm = TRUE)
brier_anxiety    <- mean((pred_anxiety - data_clean$anxiety_binary)^2,
                         na.rm = TRUE)
brier_turnover   <- mean((pred_turnover - data_clean$turnover_binary)^2,
                         na.rm = TRUE)
cat("Brier Score - Depression:", round(brier_depression, 3), "\n")
cat("Brier Score - Anxiety:   ", round(brier_anxiety, 3), "\n")
cat("Brier Score - Turnover:  ", round(brier_turnover, 3), "\n")

# 12.5 10-Fold Cross-Validation for Depression (grouped by facility)
set.seed(20250731)
folds <- groupKFold(data_clean$Facility_Code, k = 10)
cv_auc_depression   <- numeric(length(folds))
```

```r
cv_brier_depression <- numeric(length(folds))

for (i in seq_along(folds)) {
  train_data <- data_clean[-folds[[i]], ]
  test_data  <- data_clean[folds[[i]], ]

  cv_model <- glmer(
    depression_binary ~ Age + Gender + Displacement_Status +
      Monthly_Income_Adequacy + Exposure_to_Workplace_Violence +
      Nurse_Patient_Ratio + Night_Shifts_Per_Month +
      Access_to_Psychological_Support + Employment_Type +
      (1 | Facility_Code),
    data = train_data,
    family = binomial,
    control = glmerControl(optimizer = "bobyqa")
  )

  cv_pred <- predict(cv_model, newdata = test_data, type = "response",
                     allow.new.levels = TRUE)
  cv_roc  <- roc(test_data$depression_binary, cv_pred)
  cv_auc_depression[i]   <- auc(cv_roc)
  cv_brier_depression[i] <- mean((cv_pred - test_data$depression_binary)^2,
                                 na.rm = TRUE)
}
cat("10-Fold CV AUC - Depression: Mean =", round(mean(cv_auc_depression), 3),
    ", SD =", round(sd(cv_auc_depression), 3), "\n")
cat("10-Fold CV Brier - Depression: Mean =", round(mean(cv_brier_depression), 3),
    ", SD =", round(sd(cv_brier_depression), 3), "\n")

# 12.6 10-Fold CV for Anxiety
set.seed(20250731)
folds_anx <- groupKFold(data_clean$Facility_Code, k = 10)
cv_auc_anxiety   <- numeric(length(folds_anx))
cv_brier_anxiety <- numeric(length(folds_anx))

for (i in seq_along(folds_anx)) {
  train_data <- data_clean[-folds_anx[[i]], ]
  test_data  <- data_clean[folds_anx[[i]], ]

  cv_model <- glmer(
    anxiety_binary ~ Age + Gender + Displacement_Status +
      Monthly_Income_Adequacy + Exposure_to_Workplace_Violence +
      Nurse_Patient_Ratio + Night_Shifts_Per_Month +
      Access_to_Psychological_Support + Employment_Type +
      (1 | Facility_Code),
    data = train_data,
    family = binomial,
    control = glmerControl(optimizer = "bobyqa")
  )

  cv_pred <- predict(cv_model, newdata = test_data, type = "response",
                     allow.new.levels = TRUE)
  cv_roc  <- roc(test_data$anxiety_binary, cv_pred)
  cv_auc_anxiety[i]   <- auc(cv_roc)
  cv_brier_anxiety[i] <- mean((cv_pred - test_data$anxiety_binary)^2,
                              na.rm = TRUE)
}
cat("10-Fold CV AUC - Anxiety: Mean =", round(mean(cv_auc_anxiety), 3),
    ", SD =", round(sd(cv_auc_anxiety), 3), "\n")
cat("10-Fold CV Brier - Anxiety: Mean =", round(mean(cv_brier_anxiety), 3),
    ", SD =", round(sd(cv_brier_anxiety), 3), "\n")

# 12.7 10-Fold CV for Turnover
set.seed(20250731)
folds_to <- groupKFold(data_clean$Facility_Code, k = 10)
cv_auc_turnover   <- numeric(length(folds_to))
cv_brier_turnover <- numeric(length(folds_to))

for (i in seq_along(folds_to)) {
```

```
  train_data <- data_clean[-folds_to[[i]], ]
  test_data  <- data_clean[folds_to[[i]], ]

  cv_model <- glmer(
    turnover_binary ~ Displacement_Status + Monthly_Income_Adequacy +
      Exposure_to_Workplace_Violence + Nurse_Patient_Ratio +
      Access_to_Psychological_Support + Employment_Type + Age + Gender +
      (1 | Facility_Code),
    data = train_data,
    family = binomial,
    control = glmerControl(optimizer = "bobyqa")
  )

  cv_pred <- predict(cv_model, newdata = test_data, type = "response",
                     allow.new.levels = TRUE)
  cv_roc  <- roc(test_data$turnover_binary, cv_pred)
  cv_auc_turnover[i]   <- auc(cv_roc)
  cv_brier_turnover[i] <- mean((cv_pred - test_data$turnover_binary)^2,
                               na.rm = TRUE)
}
cat("10-Fold CV AUC - Turnover: Mean =", round(mean(cv_auc_turnover), 3),
    ", SD =", round(sd(cv_auc_turnover), 3), "\n")
cat("10-Fold CV Brier - Turnover: Mean =", round(mean(cv_brier_turnover), 3),
    ", SD =", round(sd(cv_brier_turnover), 3), "\n")

# 12.8 Summary Table for Model Performance
model_performance <- data.frame(
  Outcome        = c("Depression", "Anxiety", "Turnover"),
  Apparent_AUC   = c(round(auc_depression_apparent, 3),
                     round(auc_anxiety_apparent, 3),
                     round(auc_turnover_apparent, 3)),
  CV_AUC_Mean    = c(round(mean(cv_auc_depression), 3),
                     round(mean(cv_auc_anxiety), 3),
                     round(mean(cv_auc_turnover), 3)),
  CV_AUC_SD      = c(round(sd(cv_auc_depression), 3),
                     round(sd(cv_auc_anxiety), 3),
                     round(sd(cv_auc_turnover), 3)),
  Brier_Score    = c(round(brier_depression, 3),
                     round(brier_anxiety, 3),
                     round(brier_turnover, 3))
)
print(model_performance)
write_csv(model_performance, "results/model_performance.csv")


# ============================================================================
# 13. FIGURE GENERATION
# ============================================================================

# 13.1 Figure 1: Prevalence bar chart
fig1 <- ggplot(data_clean, aes(x = factor(depression_binary))) +
  geom_bar(aes(y = after_stat(count / sum(count)), fill = "#3498DB")) +
  scale_y_continuous(labels = scales::percent) +
  labs(x = "Depression Status", y = "Percentage",
       title = "Prevalence of Depression") +
  theme_minimal()

# 13.2 Figure 2: Forest plot
fig2 <- plot_model(model_depression, show_values = TRUE,
                   value.offset = .3, vline.color = "grey",
                   title = "Adjusted Odds Ratios for Depression")

# 13.3 Figure 3: Interaction plot
fig3 <- interact_plot(moderation_model,
                      pred = Displacement_Status,
                      modx = Access_to_Psychological_Support,
                      interval = TRUE, int.width = 0.95,
                      title = "Moderation by Psychological Support")
```

```
# 13.4 Figure 4: SEM diagram (only if SEM model is defined)
# NOTE: The SEM model must be specified using lavaan before running this.
# Example specification (adjust to your conceptual model):
sem_model_syntax <- '
  # Measurement model
  Depression =~ PHQ9_Item1 + PHQ9_Item2 + PHQ9_Item3
  Anxiety    =~ GAD7_Item1 + GAD7_Item2 + GAD7_Item3
  Burnout    =~ Burnout_Item1 + Burnout_Item2 + Burnout_Item3

  # Structural model
  Depression ~ Burnout + Displacement_Status + Exposure_to_Workplace_Violence
  Anxiety    ~ Burnout + Displacement_Status + Exposure_to_Workplace_Violence
  Burnout    ~ Displacement_Status + Exposure_to_Workplace_Violence
'

sem_fit <- sem(sem_model_syntax, data = data_clean, estimator = "MLR")
summary(sem_fit, fit.measures = TRUE, standardized = TRUE)

sem_paths <- semPlot::semPaths(sem_fit, what = "est", layout = "tree",
                               edge.label.cex = 1.2)


# ============================================================================
# 14. EXPORT RESULTS
# ============================================================================

# 14.1 Save tables
saveRDS(table1, "results/table1.rds")
saveRDS(table2, "results/table2.rds")
write_csv(table_S12, "results/table_S12_weighted_prevalence.csv")
write_csv(comparison_table, "results/table_S12_comparison.csv")
write_csv(model_performance, "results/model_performance.csv")

# 14.2 Save models
save(model_depression, model_anxiety,
     model_turnover_model1, model_turnover_model2,
     model_phq_linear, model_gad_linear, model_burnout_ordinal,
     file = "results/main_models.RData")

# 14.3 Save mediation and SEM results
save(sem_fit, med_results, med_results2,
     file = "results/mediation_models.RData")

# 14.4 Save figures
ggsave("figures/figure1_prevalence.png", fig1, width = 8, height = 6)
ggsave("figures/figure2_forest_plot.png", fig2, width = 10, height = 8)
ggsave("figures/figure3_interaction.png", fig3, width = 8, height = 6)

# 14.5 Save sensitivity results
save(rr_robust, rr_robust_anxiety,
     model_depression_mild, model_anxiety_moderate, model_no_outliers,
     harman_variance,
     range_check_phq9, range_check_gad7, consistency_check_age,
     model_performance,
     model_depression_weighted, model_anxiety_weighted, model_turnover_weighted,
     weighted_prev_depression, weighted_prev_anxiety,
     weighted_prev_burnout, weighted_prev_turnover,
     file = "results/sensitivity_analyses.RData")


# ============================================================================
# 15. REPORT GENERATION
# ============================================================================

rmarkdown::render("analysis_report.Rmd",
                  output_file = "final_analysis_report.html")


# ============================================================================
```

```
# 16. SESSION INFO
# =============================================================================

sessionInfo()
```
