# Supplementary material for: Mental Health, Burnout, and Turnover Intention Among Nurses in Conflict-Affected Yemen: A Multicenter Study Across Eight Accessible Governorates
Source: Nurs Rep. 2026 Oct 7;16(10):353. doi: 10.3390/nursrep16100353 (PMC13708594; doi:10.3390/nursrep16100353)
Supplement: Supplementary file 1 [file nursrep-16-00353-s001.zip › File S9 Ethical approval certificate.pdf]

جامعة لحج
UNIVERSITY OF LAHEJ

Radfan College University

Ethical Committee

Ethical Approval Certificate

**Study Title:**	Psychological Distress, Burnout, and Turnover Intent Among Nurses in Conflict-Torn Yemen: A Cross-Sectional Study
**Protocol Version:**	2.0
**IRB Reference Number:**	YEM-IRB-2025-094
**Date of Approval:**	January 15, 2025
**Expiry Date:**	January 14, 2026

This is to certify that the above-named study has been reviewed and approved by the **Institutional Review Board (IRB)** of Radfan College University, Lahej, Yemen.
The approval is granted in accordance with the principles of the **Declaration of Helsinki** and applicable local ethical guidelines.

The study is approved for the recruitment of **800** registered nurses from selected health facilities across conflict-affected governorates in Yemen, using the approved protocol and informed consent documents.

CONDITIONS OF APPROVAL:

- Written informed consent must be obtained from all participants.
- Participant anonymity and confidentiality must be strictly maintained.
- Any protocol modifications require prior IRB approval.
- A final report must be submitted upon study completion.

Radfan College University
Ethical Committee

Chairperson, Institutional Review Board
Radfan College University
Lahej, Yemen

IRB Administrator
Dr. Adel Ahmed Ali
