# Supplementary material for: Mental Health, Burnout, and Turnover Intention Among Nurses in Conflict-Affected Yemen: A Multicenter Study Across Eight Accessible Governorates
Source: Nurs Rep. 2026 Oct 7;16(10):353. doi: 10.3390/nursrep16100353 (PMC13708594; doi:10.3390/nursrep16100353)
Supplement: Supplementary file 1 [file nursrep-16-00353-s001.zip › File S10 Facility Level Characteristics22.pdf]

Supplementary File

Mental Health, Burnout and Turnover Intention among Nurses in Conflict-Affected Yemen: A Multicentre Study Across Eight Accessible Governorates

Corresponding Author: Naif Taleb Ali

2. Table S12a — Facility-Level Characteristics

3. Table S12b — Sampling Details by Region (Aggregated)

4. Table S12c — Security Level Assessments by Governorate

5. Table S12d — Facility-Level Response Rates (Summary Statistics)

6. Notes on Data Sources and Revisions

7. Cross-References

8. Limitations

9. References

from the Ministry of Public Health and Population (MoPHP) and the Health Cluster Yemen [37].

Purpose: This file supports the manuscript's sampling description (Methods Section 2.3) and provides full transparency regarding the facility-level sampling frame, facility characteristics, and response rates.

Important Note on Facility Codes: Facility codes (F01–F24) were assigned within governorates and are not globally unique across the entire study. This means the same facility code (e.g., F01) may appear in more than one governorate. Facility identity is defined by the combination of Governorate_Code + Facility_Code + Cluster_ID. This is documented in the Data Dictionary (File S6).

2. Table S12a — Facility-Level Characteristics

Facility Code	Governorate	Region	Ownership	Level of Care	Eligible Nurses	Nurses Invited	Nurses Participated	Response Rate (%)
F01	Aden	South	Public	Tertiary	40	40	37	92.5
F02	Aden	South	Public	Secondary	35	35	32	91.4
F03	Aden	South	Private	Secondary	34	34	31	91.2
F04	Lahj	South	Public	Tertiary	36	36	34	94.4
F05	Lahj	South	Public	Secondary	36	36	34	94.4
F06	Lahj	South	Private	Secondary	35	35	32	91.4
F07	Abyan	South	Public	Tertiary	36	36	34	94.4
F08	Abyan	South	Public	Secondary	36	36	34	94.4
F09	Abyan	South	Private	Secondary	35	35	32	91.4
F10	Shabwah	Central	Public	Tertiary	36	36	34	94.4
F11	Shabwah	Central	Public	Secondary	36	36	34	94.4
F12	Shabwah	Central	Private	Secondary	35	35	32	91.4
F13	Hadramawt	East	Public	Tertiary	36	36	34	94.4
F14	Hadramawt	East	Public	Secondary	36	36	34	94.4
F15	Hadramawt	East	Private	Secondary	35	35	32	91.4
F16	Al-Mahra	East	Public	Tertiary	36	36	34	94.4
F17	Al-Mahra	East	Public	Secondary	36	36	34	94.4
F18	Al-Mahra	East	Private	Secondary	35	35	32	91.4
F19	Socotra	East	Public	Tertiary	36	36	34	94.4
F20	Socotra	East	Public	Secondary	36	36	34	94.4
F21	Socotra	East	Private	Secondary	35	35	32	91.4
F22	Taiz	Central	Public	Tertiary	36	36	34	94.4
F23	Taiz	Central	Public	Secondary	36	36	34	94.4
F24	Taiz	Central	Private	Secondary	35	35	32	91.4

Note: Response Rate (%) is calculated as (Nurses Participated / Nurses Invited) × 100 at the facility level. The term "sampling fraction" is not used in this table because within each facility every second eligible nurse was invited, corresponding to a within-facility sampling fraction of approximately 0.50. The overall response rate at the

South	Aden, Lahj, Abyan	9	318	29	29	347	289	83.3	36.12
Central	Shabwah, Taiz	6	226	13	13	239	213	89.1	26.63
East	Hadramawt, Al-Mahra, Socotra	9	328	30	30	358	298	83.2	37.25
Total	8 governorates	24	872	72	72	944	800	84.7	100.00

Note: The initial systematic random sampling sequence approached 872 eligible nurses across the eight accessible governorates. Of these, 72 did not participate (42 declined, 18 were on emergency duty, and 12 were administratively unavailable) and were replaced by the next eligible nurses on the sampling list, resulting in 72 additional nurses being approached. The total number of nurses approached was therefore 944 (872 + 72), of whom 800 participated, yielding an overall response rate of 84.7% (800/944). Approximately equal allocation (~100 nurses per governorate) was implemented to ensure adequate statistical power for governorate-level comparisons. Response rates are calculated as (Nurses Participated) / (Total Approached) × 100. Region-level aggregation is presented to protect the confidentiality of individual governorates with small nursing workforces (e.g., Socotra, Al-Mahra).

4. Table S12c — Security Level Assessments by Governorate

Governorate	Region	Security Level	Security Assessment Date	Facilities Excluded (n)	Reason for Exclusion
Aden	South	Moderate	December 2024	0	—
Lahj	South	Moderate	December 2024	0	—
Abyan	South	Moderate	December 2024	1	Access constraints
Shabwah	Central	Moderate	December 2024	0	—
Hadramawt	East	Moderate	December 2024	0	—
Al-Mahra	East	Moderate	December 2024	0	—
Socotra	East	Moderate	December 2024	0	—
Taiz	Central	Moderate-High	December 2024	2	Active conflict nearby

Notes:

Security levels were assessed by the study team in coordination with local health authorities and humanitarian partners (Health Cluster Yemen).

[illegible]

5. Table S12d — Facility-Level Response Rates (Summary Statistics)

Statistic	Value
Number of facilities	24
Minimum facility-level response rate	91.2%
Maximum facility-level response rate	94.4%
Mean facility-level response rate	93.2%
Median facility-level response rate	94.4%
Standard deviation	1.5%
Overall participation rate	84.7% (800/944)

Distribution of Facility-Level Response Rates:

Response Rate Range	Number of Facilities
88.0% – 89.9%	0
90.0% – 91.9%	9
92.0% – 93.9%	1
94.0% – 95.9%	14

Notes:

Explanation of Nurse Numbers:

872 = Number of nurses originally approached through the systematic sampling sequence.

72 = Number of non-participants who were replaced (42 declined, 18 on emergency duty, 12 administratively unavailable).

72 = Number of additional nurses approached as replacements.

944 = Total number of nurses approached (872 original + 72 replacements).

800 = Final analytical sample (nurses who completed the questionnaire successfully).

Response Rate Calculation:

The overall participation rate is 84.7% (800/944), reflecting the total number of nurses approached, including both the original sequence and the replacement sequence.

The previously reported 91.7% response rate has been removed because it did not account for the 72 replacement participants.

Facility-level response rates ranged from 91.2% to 94.4%, indicating consistently high participation across all 24 facilities.

Replacements are included in the final analytical sample of 800 and are counted in the denominator of 944, which reflects the total number of nurses approached.

Note on Aggregation:

The mean facility-level response rate (93.2%) reflects the average response rate across the 24 facilities.

The overall participation rate (84.7%) reflects the ratio of participants (800) to the total number of nurses approached (944), including all replacements.

These two figures describe different levels of aggregation and are not directly comparable.

6. Notes on Data Sources and Revisions

6.1 Data Sources

Facility list: Ministry of Public Health and Population (MoPHP), updated December 2024.

Registered nurse list: MoPHP, updated December 2024.

Health facility mapping: Health Cluster Yemen, December 2024.

Security assessments: Study team in coordination with local health authorities and humanitarian partners, December 2024.

6.2 Facility Selection Criteria

Facilities were eligible for inclusion if they:

Were functioning at the time of data collection.

Were accessible based on security assessments.

Had at least 10 registered nurses on staff.

Provided direct patient care.

6.3 Sampling Procedure

Stage 1: Selection of 8 accessible conflict-affected governorates.

Stage 2: Stratification of facilities by ownership (public/private) and level of care (primary/secondary/tertiary).

Stage 3: Random selection of 3 facilities per governorate, yielding 24 facilities in total.

Stage 4: Systematic random sampling of nurses within each facility (every second eligible nurse invited).

Facility identity is defined by the combination of Governorate_Code + Facility_Code + Cluster_ID.

This is documented in the Data Dictionary (File S6).

6.5 Revisions from Previous Version

This version of File S10 incorporates the following changes:

Complete facility-level characteristics: Table S12a now provides detailed characteristics for all 24 facilities.

Region-level aggregation: Table S12b presents sampling details aggregated by region (South, Central, East) to protect the confidentiality of individual governorates with small nursing workforces.

Removed incorrect "sampling fraction" column: The previous column that incorrectly reported a "sampling fraction" (which was in fact a response proportion) has been removed from Table S12a.

Corrected response rate terminology: The distinction between facility-level response rates (mean 93.2%) and the overall participation rate (84.7%, 800/944) is now clearly stated.

Security assessments: Table S12c documents security levels and exclusion reasons.

Response rate distribution: Table S12d provides facility-level response rate statistics.

Consistency with manuscript: All tables are cross-referenced with the manuscript's Methods Section 2.3.

File numbering: This file is designated File S10, consistent with the definitive numbering system.

7. Cross-References

Reference	Location	Content
Methods 2.3	Manuscript	Sampling strategy
Table S12	File S4	Sampling-weighted prevalence estimates
Table S9	File S4	Aggregated sampling details by governorate
Table S12a	File S10	Facility-level characteristics (this file)
Table S12b	File S10	Aggregated sampling details by region (this file)
Table S12c	File S10	Security level assessments (this file)
Table S12d	File S10	Facility-level response rates (this file)

8. Limitations

Estimated eligible nurses: The number of eligible nurses per facility was estimated from official MoPHP records and may not reflect the exact number at the time of data collection [37].

Facility code non-uniqueness: Facility codes are not globally unique across governorates, which may complicate data linkage. Facility identity requires the combination of Governorate_Code + Facility_Code + Cluster_ID

selection probabilities differed across governorates. Sampling-weighted prevalence estimates and regression models are presented in Table S12 (File S4) and Section 9.8 of File S8. The weighted estimates were nearly identical to the unweighted estimates (absolute differences < 1 percentage point), supporting the robustness of the findings to differential selection probabilities.

9. References

38. Ministry of Public Health and Population (MoPHP), Yemen. Health Facilities Database. Sana’a, Yemen: Ministry of Public Health and Population; 2024. Available online: https://moh.gov.ye/facilities-details (accessed on 26 September 2026).
